# Identifying Yalom’s group therapeutic factors in anonymous mental health discussions on Reddit: a mixed-methods analysis using large language models, topic modeling and human supervision

**DOI:** 10.3389/fpsyt.2025.1503427

**Published:** 2025-06-09

**Authors:** Drin Ferizaj, Christopher Lalk, Nils Lahmann, Sandra Strube-Lahmann, Julian Rubel

**Affiliations:** ^1^ Department of Geriatrics and Medical Gerontology, Charité—Universitätsmedizin Berlin, corporate member of Freie Universität Berlin and Humboldt-Universität Zu Berlin, Berlin, Germany; ^2^ Department of Psychology, Osnabrück University, Osnabrück, Germany; ^3^ Studiengang Medizinpädagogik, Fakultät Naturwissenschaften, MSB Medical School Berlin, Hochschule für Gesundheit und Medizin, Berlin, Germany; ^4^ Stabsstelle Pflegewissenschaft, Universitätsmedizin Göttingen, Georg-August-Universität, Pflege- und Pflegefunktionsdienst, Göttingen, Germany

**Keywords:** large language models, thematic analysis, topic modeling, mental health, group therapy, peer support, online communities

## Abstract

**Introduction:**

Online communities provide valuable, peer-led spaces for discussing mental health issues, offering support that can complement traditional therapy. In this study, we adopt an interpretive approach by applying Yalom's group therapeutic factors to explore how mental health–focused Reddit discussions may reflect group therapy processes.

**Methods:**

We propose a practical methodological framework for large-scale qualitative research. Using a mixed-methods approach, we integrate advanced Natural Language Processing (NLP) techniques—including Large Language Models (GPT-3.5 Turbo 16k), cosine similarity, and BERTopic—with human validation to analyze 6,745 comments from mental health–focused Subreddits.

**Results:**

The results show that a large portion of the data can be interpreted through Yalom's therapeutic factors, such as Instillation of Hope, Group Cohesion, and Altruism, suggesting a generally supportive and empathetic online environment. However, unfiltered negative dynamics, including shared suffering and maladaptive coping strategies, also appeared in the discussions.

**Discussion:**

By grounding NLP-based analyses in a well-established therapeutic framework and incorporating human expertise, we demonstrate a transparent, scalable approach to examining large-scale online mental health data. These findings underscore the potential of online communities for enhancing peer-led mental health support, while emphasizing the importance of theoretical grounding in interpreting such digital spaces.

## 1 Introduction

Internet forums like Reddit, Tumblr, Facebook, and Twitter offer users spaces to share ideas and experiences. This includes conversations on mental health, allowing researchers to explore issues like difficulties, resources, peer-support, and communication (1–3). Mental health forums offer complementary support alongside counseling or psychiatric treatment (4) and are especially valuable for younger users who frequently access important information online (5). With the widespread availability of social media and internet access (6), online forums serve as an accessible supplement to traditional mental health care for individuals who face barriers to in-person treatment. The perceived—and sometimes enforced—anonymity in these forums facilitates the sharing of private thoughts, emotions, and experiences, aligning with the ‘Online Disinhibition Effect’ (5). Moreover, these internet forums are not just diluted versions of offline interactions but constitute distinct communicative patterns and mechanisms. According to the hyperpersonal model, the absence of immediate nonverbal cues in computer-mediated communication can lead to greater intimacy and self-disclosure than face-to-face interactions (7). Users in asynchronous online settings such as Reddit may benefit from the opportunity to reflect on and edit their messages, resulting in more thoughtful and emotionally charged exchanges. Reddit, with its subcommunities (subreddits), upvoting systems, and self-moderation, aligns with the hyperpersonal model by enabling highly specialized and self-selecting interactions (8). Similarly, this structure not only facilitates personal exchanges but also supports a unique dynamic of self-disclosure, as users often choose to remain pseudonymous or use “throwaway” accounts. This kind of anonymity has been shown to reduce fear of judgment and stigma, allowing for a more open discussion of sensitive mental health issues—a finding supported by De Choudhury and De (9). Additionally, Reddit’s user base tends to be younger and more tech-savvy than that of traditional forums, and the platform attracts users with diverse motivations—from seeking peer support and practical advice to engaging in self-help discussions—thus representing a unique segment of the online sphere (8, 9).

Furthermore, research has shown that shared experiences and community identity can build trust and even strengthen long-term relationships among forum users (10). This may reduce isolation (11) and stress (12) and even improve symptoms of depression (11) and self-harm (13). In this context, the possibility to discuss experiences and receive practical advice in nontechnical, down-to-earth language further attracts members (14). Discussions cover diverse topics like coping, identity management (15), and positive coping strategies such as maintaining relationships and activities (16). Although the overall effects of online peer support remain mixed (17, 18), comparing traditional group therapy with online forums reveals similarities. In his seminal work, Yalom (19) outlines several interdependent mechanisms in group therapy—many of which are also evident in online peer support settings:

### 1.1 Instillation of hope

Online access enables connections with others who have similar, even rare, experiences. This may also lead to exchange with users who already have improved or even recovered. These individuals can provide a role model on the road to recovery and instill hope in their peers (20, 21). This is crucial during setbacks, showing they are part of recovery (21). For online depression support communities, preliminary research has shown emotional contagion for positive emotions (22). Even in the case of suicidality, there is a preventive Papageno effect that is caused by the promotion of adaptive coping in suicidal crises (23).

### 1.2 Universality (of suffering)

Suffering, especially in the context of mental health, can lead to isolation and social withdrawal (24) due to the attached stigma and psychiatric symptoms (25). While suffering may increase isolation, shared experiences can enable connections and positive relationships, often found in online forums (16). Recognizing that others share one’s experiences can normalize them and lead to better coping strategies, a process termed communal normalization (26). Research shows individuals seek advice in forums from peers “in the same boat as me” (14).

### 1.3 Imparting of information

One of the foremost purposes of the internet has been the almost universal access to information (5). Regarding mental health, help-seekers specifically search for information pertaining to practical support, and the mental health care system (12, 27). McKiernan et al. (28) show a preference for practical information focused on one’s own experience instead of general or more directive advice. Thus, online forums offer less directive, more practical information, complementing traditional healthcare. However, this has to be weighed against the risk of receiving misleading information (29), especially since personal and practical advice can be biased (30).

### 1.4 Altruism

Similar to group therapy, online communities provide help-seekers with the opportunity to take a supportive role (31). This can be meaningful and beneficial, fostering a new self-concept beyond just being a help-seeker. However, limited data exist on online altruism, but it can be effective in group therapy. For instance, in cognitive behavior group therapy for OCD, reported altruistic behavior predicted improvement (32).

### 1.5 Group cohesion

Cohesion is the most important mechanism of action in the literature on groups (33). It can be defined as a measure of the relationships in the group. Cohesion is vital in online mental health communities, as young people report a sense of belonging and connection as key reasons for forum use (34). In another study, participants stressed the importance of overcoming isolation and connecting via the forum (35). Many found it hard to talk to friends or family, fearing annoyance, and stressed the need for conversations with those who share similar issues. Generally, a shared sense of community identity can foster interpersonal trust (10). This strengthens usage intention and relationships, crucial for symptom improvement, as active participation often predicts better outcomes (11).

### 1.6 Catharsis

Catharsis refers to the process of releasing strong or repressed emotions, both positive and negative, thereby providing relief (19). Research showed that writing about a distressing or challenging topic can be therapeutic in itself (36). Writing may enhance emotion regulation by fostering insight and personal agency. Also, users of online counseling described the benefits of expressing their concerns and struggles via writing as therapeutic (37).

Yalom (19) formulated additional group therapy mechanisms, such as the simulation of primary family, interpersonal learning, imitative behavior, and development of social skills, which may be less readily observed in online settings due to the lack of immediacy, face-to-face interactions, and a therapist’s guidance. Indeed, a study of an asynchronous, peer-led support forum for caregivers found that 9 of Yalom’s 11 factors emerged, but those requiring real-time interaction (e.g. interpersonal learning, family reenactment) were virtually absent (20).

In parallel with the growth of online support communities, computational methods in the field of natural language processing (NLP) have facilitated the automated analysis of large-scale text data. NLP utilizes rule-based, statistical, and neural network techniques to perform tasks such as speech recognition and text classification, with applications ranging from sentiment analysis to machine translation and conversational agents (38). Topic modeling has become a popular approach to analyze large qualitative datasets from online discussions as it allows for the automatic clustering of semantically similar concepts (39, 40). For instance, De Choudhury and De (9) analyzed about 90,000 forum contributions and found that users engage in self-disclosure and provide emotional, informational, instrumental, and prescriptive forms of support. Furthermore, other large-scale topic modeling research has highlighted that within mental health online communities, discussions of social and psychological factors related to mental health conditions and symptoms are accentuated, while biomedical topics receive comparatively less emphasis (39, 41, 42). Other studies have combined topic modeling with qualitative discourse analysis, finding that supervised machine learning can classify large corpora into biomedical, psychological, and sociological discourses, yet struggles with sociological framing—indicating hermeneutic limitations (43). Similarly, a study analyzing 80,000 posts to classify depression framing into bio-medical, psychological, and social categories revealed that discursive complexity challenges annotation consistency and predictive accuracy. This shows the importance of human supervision when dealing with large quantities of qualitative data (44). These mixed-methods approaches have potential to deepen the understanding of how people actually think and talk about mental health issues. Still, data-based techniques generally have limited capabilities in capturing semantic context and nuance (45, 46), especially important in therapeutic processes. Therefore, when conducting qualitative big data analyses, one should consider that meaning is not a fixed attribute inherent to language but is actively co-created through consensus and intersubjectivity, emphasizing the importance of grounding big-data techniques in theoretical frameworks while also integrating human perspectives when interpreting these data (44).

Despite their utility, topic models come with inherent limitations. Their unsupervised nature and limited language understanding often necessitate extensive *post-hoc* qualitative analysis to extract meaningful insights. Although topic models can effectively identify statistical patterns, they often fail to capture semantic and pragmatic nuances in human language, particularly in sensitive contexts such as mental health discussions. This limitation can result in misinterpretations, reduced contextual awareness, and difficulties in discerning emotional tone or sentiment (45, 46).

More recently, large language models (LLMs) have emerged as promising tools for analyzing large text corpora, enabling qualitative data processing at scale (47–49). Properly prompted, LLMs can conduct large-scale thematic analyses far more quickly than traditional human coding approaches (47, 48, 50). For example, Prescott et al. (50) found that ChatGPT efficiently identified overarching themes with moderate agreement to human coders—though human expertise remained vital for capturing subtle, context-dependent insights. Meanwhile, Deiner et al. (47) highlighted that repeated LLM analyses can vary, underscoring the need for human validation to ensure consistency. In a recent study, Lee et al. (48) outlined a three-phase approach—direct coding of transcripts, generating themes from predefined codes, and preprocessing quotes for manuscripts—demonstrating how ChatGPT can expedite the thematic analysis workflow while still requiring human oversight to address nuances, cultural context, and potential misinterpretations. Overall, the literature emphasizes that LLMs may overlook cultural subtleties and rely on opaque “black box” processing; thus, strategies such as direct coding of raw data, which provides an explicit audit trail, are instrumental in enhancing explainability and supporting rigorous human validation.

Building on these insights, the present study employs a hybrid, human-in-the-loop methodology that combines the efficiency of LLM-based coding and human qualitative validation and interpretation. Specifically, we use an LLM to segment Reddit comments into clear, concise meaning units, each tagged with a respective code. These codes are then aggregated via BERTopic (51), generating semantically coherent clusters. Human experts subsequently validate and refine these clusters and perform manual thematic analysis based on Yalom’s theoretical framework (19), addressing limitations noted in previous research, wherein purely computational methods may capture concrete patterns but miss the more abstract or context-dependent dimensions that are critical to online mental health discourse.

Accordingly, this study has two main objectives: The primary objective is to apply Yalom’s framework to Reddit-based mental health discussions using an interpretive approach to map user-generated content to group therapeutic factors. A secondary objective is to advance the field by introducing a transparent, human-in-the-loop workflow that merges automated LLM coding with in-depth qualitative synthesis. This method balances computational efficiency with the contextual nuance essential for high-quality qualitative research.

## 2 Materials and methods

### 2.1 Data and ethical considerations

Ethical approval was not required for the study involving human data in accordance with the local legislation and institutional requirements. Written informed consent was not required, for either participation in the study or for the publication of potentially/indirectly identifying information, in accordance with the local legislation and institutional requirements. The social media data were accessed and analyzed in accordance with the platform’s terms of use and all relevant institutional/national regulations.

Reddit, a popular social media platform, hosts numerous user-driven communities called Subreddits, where people with shared interests discuss topics and offer support. With millions of active users globally, Reddit facilitates meaningful conversations on diverse subjects, including mental health. Its voting system, where users upvote or downvote posts and comments, generates net scores that highlight the most helpful and insightful contributions (8).

This study analyzed 6,745 comments extracted from a broad range of mental health-focused Subreddits, including ‘Depression’, ‘Anxiety’, ‘Stress’, ‘CasualConversation’, ‘AnxietyDepression’, ‘SuicideWatch’, ‘MentalHealth’, ‘PTSD’, ‘CPTSD’, ‘PanicAttack’, ‘AnxietyHelp’, ‘AnxietySuccess’, ‘Depressed’, and ‘Depression_Help’. The initial dataset comprised 7,000 top-rated comments and was collected on March 15, 2024, to retrospectively extract the top-rated comments from the selected mental health-focused subreddits. Data collection continued until a total of 7,000 entries was reached. Consequently, the resulting dataset represents a snapshot of available data up to the extraction date, rather than being limited to comments generated on that specific day. Due to budget constraints and the labor-intensive nature of human validation as well as thematic analysis, we opted to include only the top-rated comment from each post. This strategy was designed to capture the contributions that the community itself deems most helpful, also reflecting the comments that receive the most visibility. While this practical decision allowed us to balance comprehensive coverage with resource limitations, it may have introduced a bias toward more positively received viewpoints, as less popular or more contentious perspectives might be underrepresented.

Data scraping was performed using the asynchronous Python Reddit API Wrapper (PRAW; 52). To address data scarcity and minimize privacy risks, we strictly extracted the raw text of the comment bodies, intentionally omitting usernames, timestamps, and other metadata. Named entities in the raw data were anonymized with the GLiNER algorithm (53). In conducting this research, the authors carefully weighed the risks against the potential benefits, considering how the underlying resource-based approach could identify psychotherapeutic processes in self-organized communities. Despite Reddit’s open data policy allowing for research purposes (54), we took several steps to ensure ethical integrity, respecting user privacy and minimizing potential harm, as explained in detail in **Supplementary Appendix D**. In summary, our approach included the use of the GLiNER algorithm for anonymizing data (53), manual reviews to remove potentially identifying information, and paraphrasing of all quotes in the manuscript to prevent potential re-identification through reverse searching. Additionally, we considered subreddit community guidelines, excluding those with explicit anti-research policies. Additionally, we plan to disseminate our findings in a manner informed by the community. This comprehensive approach was derived from recommended practices (55, 56).

### 2.2 Natural language processing

#### 2.2.1 Large language model

LLMs are deep learning models based on the transformer architecture (57) that process human language by predicting the next token in a sequence. Trained on immense amounts of text data, they perform extremely well on various NLP tasks, such as text generation, classification, summarization, and translation (58). The transformer’s self-attention mechanism enables efficient parallel token processing, greatly enhancing its ability to capture long-range contexts, overcoming limitations of earlier architectures such as multi-layer perceptrons and recurrent neural networks (59).

LLMs often rely on a decoder-only transformer architecture, generating text token by token based on previously predicted tokens (59, 60). This design sometimes leads to coherent yet factually incorrect outputs, commonly referred to as hallucinations (61). To address this limitation while taking advantage of the autoregressive design, several strategies have been developed, including chain-of-thought prompting (62) and few-shot prompting (60).

Both methods rely on in-context learning, where the model adapts to new tasks without specialized fine-tuning. By parsing instructions and examples embedded within a prompt, the model generates solutions for tasks it was not specifically trained to handle. In this setting, models function as few-shot learners (60). For instance, in a sentiment analysis task, a prompt might include two labeled comments—one marked as positive and another as negative—followed by an unlabeled tweet. The model compares the unlabeled tweet with the provided examples to infer its sentiment.

#### 2.2.2 Cosine similarity

Cosine similarity measures the similarity between text chunks by representing them as numeric vectors and calculating the cosine of the angle between the vectors. This results in a similarity score ranging from 0 to 1, where 1 indicates identical vectors, 0 indicates orthogonality.

#### 2.2.3 BERTopic

Topic modeling is an unsupervised method for uncovering latent thematic structures in large text corpora. BERTopic, a library specifically designed for topic modeling, generates topics by utilizing transformer-based models and a three-step process that includes textual embedding, dimensionality reduction, and clustering (51). Embeddings are created using Sentence-BERT, a pre-trained transformer that transforms text chunks into dense numerical vectors (63). Afterwards, dimensionality reduction is performed with Uniform Manifold Approximation and Projection (UMAP), which considers both local and global features of the vectors (64). The clustering of the reduced dimensions is carried out using Hierarchical Density-Based Spatial Clustering of Applications with Noise (HDBSCAN), identifying clusters of varying densities and treating noise as outliers (65). In the final step, topic-word distributions are created by treating each cluster of texts as a single text and calculating term frequencies and inverse class frequencies to extract the most representative terms for each topic.

Unlike classical Latent Dirichlet Allocation—which relies on bag-of-words representations (ignoring word order and context) and requires manual specification of the number of topics—BERTopic uses contextual embeddings to capture semantic nuances such as polysemy with minimal preprocessing. Its density-based clustering automatically adapts to the data, yielding topics that are both interpretable and adaptable, often outperforming static, frequency-driven distributions like Latent Dirichlet Allocation (63, 64). For instance, BERTopic outperformed Latent Dirichlet Allocation in predicting symptom severity and therapeutic alliance while identifying key clinical themes (66).

### 2.3 Procedure

**Figure 1:**
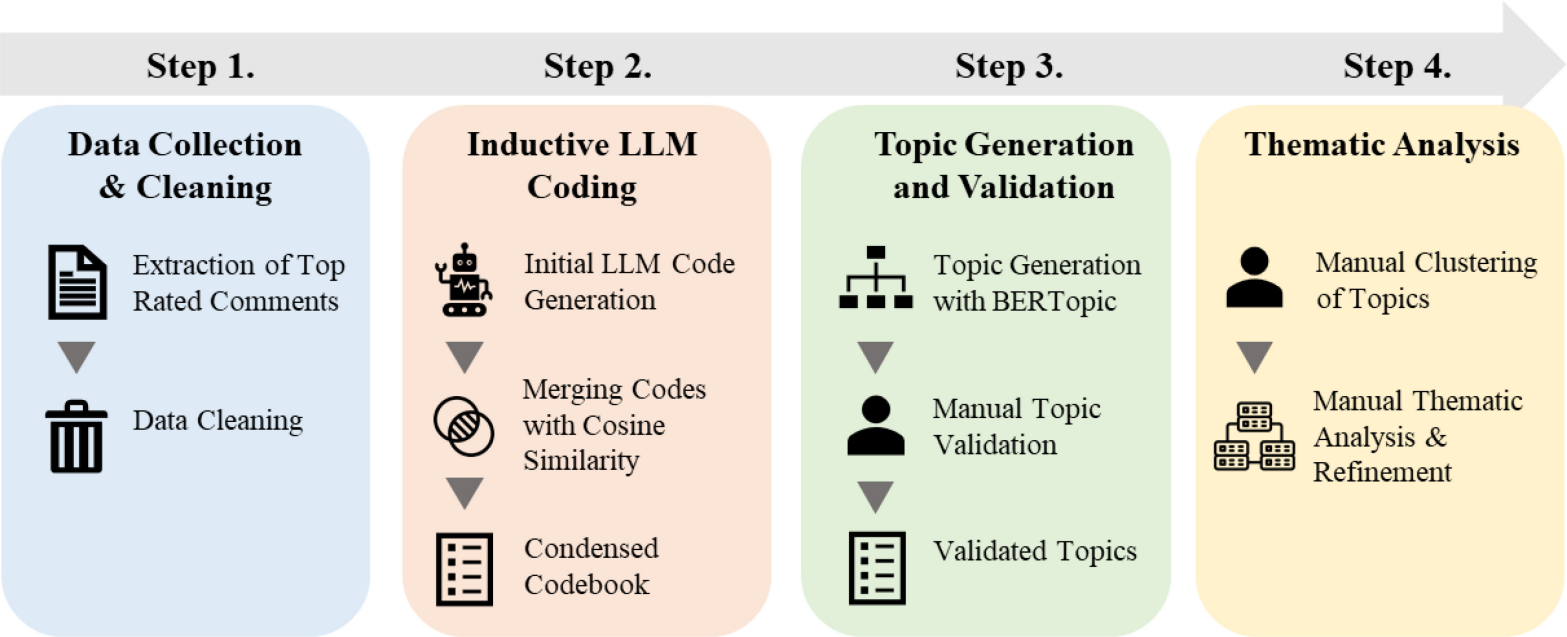
Illustration of the data analysis procedure, demonstrating the NLP methods combined with human-in-the-loop steps.

Following data extraction, data cleaning removed deleted comments and duplicate records with identical posts and responses. No further NLP preprocessing was conducted due to the extensive language understanding of the utilized LLM. The overall data analysis procedure employed a thematic analysis framework, following a modified six-step approach from Braun and Clarke (67), as outlined in **Figure 1**.

**Figure 2:**
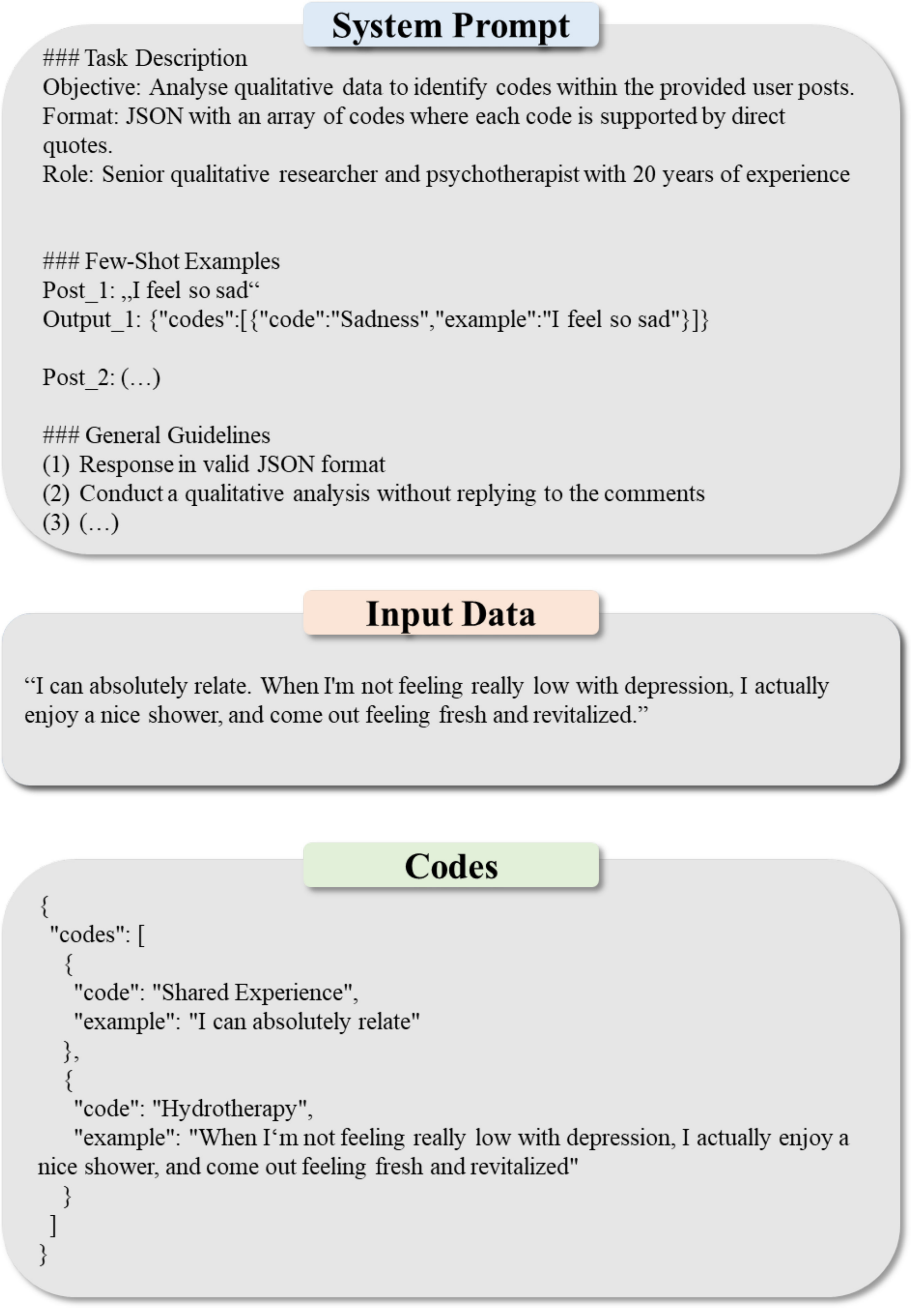
Exemplary outline of the system prompt for the qualitative LLM coding.

1. Familiarizing with Data: To gain a basic understanding of the dataset, two of the authors read through three random sets of 75 comments each.
2. Initial Code Generation: OpenAI’s GPT-3.5-turbo 16k model (68) was employed to classify each comment  c ∈ *D* (where *D* is the dataset) by assigning one or multiple codes that reflect the core aspects of the text:
codes(c)←LLM(c)
  The codes were aggregated into the set C_initial_. The basic unit of analysis for a code was defined as any contiguous text segment that conveyed a distinct meaning. We did not fix the unit size (e.g., sentence-based), allowing variability: some comments were single words (e.g., “agreed!”), while others were multi-paragraph narratives. This approach captured both brief affirmations and detailed reports, ensuring each unit was semantically complete. Prompt engineering was designed to achieve these aims through an adjusted Chain-of-Thought (62) and Few-Show-Prompting (60) framework, refined iteratively by drawing 10 random comments until it consistently identified all relevant segments and generated accurate codes. A conceptual prompt versioning protocol can be found in **Supplementary Appendix F** with the key steps being: adding segmentation to analyze contiguous meaning units, integrating diverse few-shot examples, specifying the model’s role as a qualitative analyst, implementing a strategy to prevent hallucinations by substantiating direct quotes for each code.
  Accuracy was evaluated by drawing two independent random samples of 250 code–text pairs—one representing the initial LLM coding and one representing the final coding. Each pair was rated 0 or 1 by two authors according to the following criteria: (1) Conceptual Fit (i.e., does the code capture the core meaning of the text), (2) Precision (i.e., is the code overly general or broad), (3) Context (i.e., does the text segment match the overall comment), and (4) Semantic Nature (i.e., does the code capture the semantic nuances of the original text). For the initial LLM coding, interrater reliability was assessed using Cohen’s κ with 10,000-fold bootstrapped 95% confidence intervals to account for sampling variability. Cohen’s κ is a measure of interrater reliability that accounts for the agreement occurring by chance. Its values range from -1 to 1, where 1 indicates perfect agreement, 0 represents agreement equivalent to chance, and negative values suggest less agreement than expected by chance (69). In general, values above 0.60 are considered as substantial agreement. Observed agreement was 93.1% (95% CI: 89.8%–95.9%), Cohen’s κ was 0.682 (95% CI: 0.527–0.814), and Area Under the Receiver Operating Characteristic Curve (ROC AUC) was 0.836 (95% CI: 0.751–0.915). ROC AUC quantifies the model’s ability to discriminate between classes, with values closer to 1 indicating better performance. Additionally, sensitivity (96.3%) shows that most actual positives were correctly identified, precision (95.8%) indicates that most predicted positives were true positives, and the F1‐score (96.0%) balances both sensitivity and precision into a single measure. Notably, the high precision can be attributed to the LLM’s segmentation of the raw data into clear, concise, and mostly unambiguous text segments.
  To reduce the risk of hallucinations, the LLM was prompted to substantiate each generated code with a direct quote from the raw data. The qualitative labels were extracted by prompting the LLM to format the output in a structured, machine-readable JSON format. The random samples drawn for the coding accuracy evaluation identified no hallucinations. The full prompt can be found in **Supplementary Appendix A**, with an outline of the prompt structure being illustrated in **Figure 2**. Due to LLMs’ tendency to generate semantically similar yet syntactically varied expressions, coding via LLMs was expected to yield a large pool of codes. To condense highly similar codes, cosine similarity was computed between the vector representations of these codes in C_unique_ representing the set of unique codes derived from C_initial_. All codes with a cosine similarity greater than 0.85 were grouped together. Within each group, the code with the highest rating was selected as the representative term (i.e., the label for that group), and each code was assigned exclusively to the group where it exhibited the highest similarity: Therefore, for any pair (c_j_, c_i_), if
$$\text{cos}(\theta ({\text{c}}_{j},\;{\text{c}}_{i}))\;\geq \tau \left(\tau =\;0.85\right),$$
  merge c_j_ and c_i_ into C_merged_.
3. Topic/Code Synthesis with BERTopic: Using BERTopic, the previously merged codes C_merged_ were clustered into semantically similar groups. The goal was to create precise topics that conveyed one specific meaning per topic. For example, “Depressive Mood”, “Depressive Feelings”, and “Depression” could be grouped under the topic/code “Depression”.
4. Human Validation of the generated Topics: Two of the authors manually reviewed the generated topics by BERTopic. This human validation process involved the following algorithmic strategies: Assuming the initial set of all BERTopic topics is denoted as T, each topic t ∈ T was examined according to the following criteria. If a topic t was overly broad or contained divergent codes, the authors split it into more focused sub-topics, denoted as {t_1_ , t_2_ , …, t_n_}. If t was irrelevant, it was removed from T. Similarly, if the label of t did not accurately reflect its content, it was renamed. In cases where a topic contained codes that did not align with the overall semantic category, these codes were manually evaluated to determine if it belonged to another topic; if not they were removed. Next, for each pair of topics (t, s) in T, semantically similar topics were merged. Finally, for every remaining topic t, a polarity check ensured that the qualitative orientation of the included codes was consistent. Here, polarity refers to the distinct semantic category a topic represents—for example, distinguishing between statements about improving depression and descriptions of feeling depressed. One author performed the initial validation, and the results were then checked and discussed with another author in iterative meetings until consensus was reached.
  Moreover, BERTopic automatically identifies noise—low-density, outlier data points or irrelevant information—via HDBSCAN’s density-based clustering (51), though authors manually reviewed codes in the noise topic with a frequency greater than 2 and reassigned them to fitting topics instead of full exclusion.
5. Cluster and Theme Generation and Refining: Clusters and themes were generated and refined through constant and iterative discussion among the authors. This iterative process involved identifying overarching themes that incorporated multiple clusters and interpreting each theme within the context of the underlying dataset. The authors engaged in continuous dialogue in bi-weekly meetings to resolve any discrepancies and ensure that the final set of themes was both coherent and representative of the data. After merging similar codes via cosine similarity, topic modeling, and human validation, the observed agreement was 89.5% (95% CI: 85.7%–93.0%), with Cohen’s κ decreasing slightly to 0.657 (95% CI: 0.527–0.770) and an ROC AUC of 0.797 (95% CI: 0.718–0.873). The model maintained high sensitivity (93.3%) and precision (93.8%), resulting in an F1-score of 93.6%.

## 3 Results

**Figure 3:**
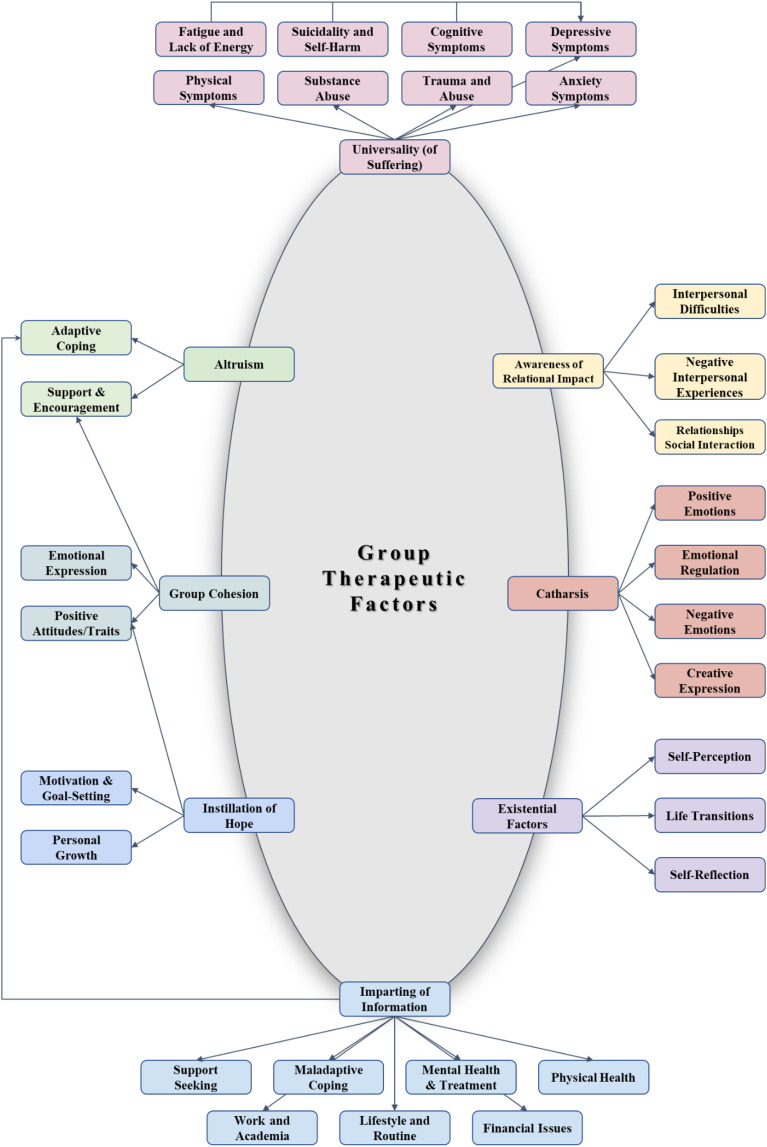
Bar chart of the 31 extracted and validated clusters, with frequency indicating the total count of all codes assigned to each cluster. Each code was exclusively assigned to a single cluster.

The initial LLM coding resulted in 24,223 codes, with 16,563 unique codes. Through cosine similarity, these were reduced to 7,097 unique codes. Topic modeling via BERTopic yielded 573 clusters, which underwent human validation to ensure thematic accuracy and coherence, leading to 589 validated topics. Further, manual thematic analysis extracted 31 clusters (**Supplementary Appendix E**). The results of the thematic analyses are illustrated schematically in **Figure 3**. These clusters were qualitatively assigned to the following group therapeutic factors postulated by Yalom: *Instillation of Hope, Universality, Awareness of Relational Impact, Imparting of Information, Altruism, Group Cohesion, Catharsis*, and *Existential Factors*. The central oval (*Group Therapeutic Factors*) represents the overarching framework derived from Yalom’s model, with each factor radiating outward to its respective clusters.

*Universality (of Suffering)* groups distress symptoms such as anxiety, depression, suicidality, trauma, and fatigue, reflecting the shared nature of many users’ experiences. *Altruism*, closely connected to *Support & Encouragement* and *Adaptive Coping*, shows how members supply one another with practical strategies (e.g., relaxation techniques, mindfulness), encouraging statements, and compassionate listening. *Group Cohesion* was interpreted through the clusters of emotional expression, positive traits, motivation, and the role of hope, highlighting the strong communal ties based on shared experiences within these forums. *Imparting of Information* consists of wide‐ranging advice, from navigating daily routines and financial strains to seeking professional treatment. *Awareness of Relational Impact* addresses interpersonal challenges and negative relational dynamics, whereas *Catharsis* encompasses positive and negative emotional expressions, creative outlets, and emotional regulation. Finally, *Existential Factors* feature topics of self‐reflection, identity, and transitions, indicating how users reflect deeper questions about personal meaning and growth.

**Table 1** provides an overview and brief description of the extracted clusters, along with the three most common codes for each cluster, accompanied by representative text examples. An extensive list with all clusters and assigned topics is illustrated in **Supplementary Appendix B**. The codes that were automatically classified by the topic model as noise are illustrated in **Supplementary Appendix C**. Throughout the results section, each cluster is followed by a notation (n = x), where x denotes the sum of all unique code counts assigned to that cluster. Within each cluster, individual codes are also illustrated with frequency counts (n = y), with y indicating how often that code appeared in the total dataset. Each code is assigned exclusively to a single topic.

**Table 1:** Overview of the validated clusters with the three most common codes for each cluster, along with representative paraphrased text examples.

Cluster (n): brief description	Codes (n)	Paraphrased examples (n)
Support and Encouragement (3313): Expressions of social support, encouragement, empathy, and validation, highlighting compassion and positive reinforcement in interactions.	Social Support (619)	“the community here is so amazing (…) you make me feel all fuzzy and warm inside.”
	Encouragement (501)	“You’re not a failure!”
	Well Wishes (252)	“Good luck to you”
Anxiety and Fear-related Constructs/Symptoms (1686): Manifestations of anxiety and fear, including stress, panic attacks, and intrusive thoughts, reflecting emotional distress.	Anxiety (584)	“My anxiety keep holding me back.”
	Stress (284)	“Surviving a 10–12 hour shift at work and staying sane.”
	Fear (258)	“My biggest fear is making one tiny mistake while driving and harming someone.”
Positive Attitudes and Character Traits (1515): Attitudes and attributes such as gratitude, hope, and resilience, emphasizing positive outlook and strength of character.	Gratitude (300)	“I am so grateful for all those who have the strength to help others.”
	Appreciation (246)	“I really needed this today.”
	Hope (235)	“I really hope everything gets better soon.”
Adaptive Coping Strategies (1297): Techniques for managing negative states, emotions, stress and adversity like mindfulness and physical activity.	Acceptance (157)	“I have finally forgiven my past and decided that even if I am behind others - it does not matter to me anymore.”
	Relaxation (147)	“Lie down in a dark room without the TV on. If you have a fan or can open a window, get some airflow going.”
	Physical Exercise and Activity (137)	“if you can, try a 15-minute workout - it can make a difference.”
Positive Emotions and Experiences (1297): Expressions of happiness and celebration, capturing joy, relief, and achievements.	Positive Experiences/Emotions (181)	“being on vacation in [location::removed] was such a nice experience.”
	Achievements and Success (170)	“I am happy to share that I have been sober for five days now.”
	Happiness (161)	“This genuinely made me really happy,”
Negative Emotions and Experiences (1258): Experiences of struggles, difficulties, and frustration, encompassing the broad spectrum of negative states and emotions.	Difficulty (279)	“It’s such a basic task, yet I can’t do it unless it’s broken into smaller steps.”
	Struggles (135)	“It’s been terrible like this for too long.”
	Frustration (131)	“It makes me so angry when people act like anything has meaning.”
Depression and Depressive Symptoms (1175): Includes general symptoms and behavioral issues of depression such as sadness, hopelessness, and emptiness.	Depression (308)	“I have been depressed for as long as I can remember,”
	Negative Thoughts (131)	“Whenever I imagine being happy, I also picture myself inevitably ruining everything again.”
	Sadness and Grief (62)	“I am in a constant state of sadness”
Emotional Expression and Communication (1173): Sharing emotions through direct communication or personal experiences.	Sharing Feelings (262)	“I opened up to my dad about how I am feeling”
	Impact (176)	“Depression is a lifelong struggle that slowly drains your soul.”
	Personal Sharing of Experiences (116)	“I experience this often as well.”
Mental Health and Treatment (921): Experiences on seeking mental health treatment, including therapy and medication.	Professional Help and Therapy (219)	“Going to the doctor, getting meds if necessary, going to therapy.”
	Medication (180)	“I’m on meds”
	Mental Health (89)	“Mental health isn’t just one thing you can easily pinpoint.”
Personal Growth and Development (837): Efforts related to improvement of symptoms and well-being as well as the personal growth’s journey.	Progress and Improvement (180)	“The more I do it, the less heavy or suffocating the depression feels when it resurfaces in a better mental state.”
	Recovery: Wishes and Efforts (74)	“I am recovering from addiction”
	Learning (64)	“Think of it as a practice run to prepare yourself.”
Relationships and Social Interaction (738): Dynamics of relationships highlighting love, affection, as well as solitude.	Affection and Love (158)	“Sending you love and light”
	Solitude (148)	“It is better to be by yourself than around people who think you are boring.”
	Connection (95)	“We all need a little connection, whether we admit it or not.”
Physical Health and Symptoms (553): Concerns related to physical health and mention of physical symptoms.	Sleep Issues and Difficulties (76)	“I can sleep all day long.”
	Symptoms (56)	“I feel like I am going to throw up.”
	Physical Illness (55)	“My muscles ache, and my stomach is always upset.”
Self-reflection and Analysis (514): Processes of self-evaluation and introspection.	(Self-)Reflection (71)	“Whenever I get a stomachache again, I ask myself what I might be anxious about.”
	Uncertainty (67)	“I am not even sure if or how I will ever move past this.”
	Questioning (45)	“What is the point of all this anyway?”
Self-perception and Identity (502): Issues and thoughts related to self-worth and identity.	Expectations (72)	“That part of your brain telling you that you shouldn’t be sad is just making things worse by guilt-tripping you.”
	Self-Worth (41)	“I hope you find your true worth one day and believe in it.”
	Self-Perception (35)	“I feel like a total failure, and it became a self-fulfilling prophecy.”
Motivation and Goal-Setting (446): Factors driving action and goal-setting as well as the realization of personal goals.	Motivation (163)	“You’re in charge of your life and mind, keep telling yourself that!”
	(Taking) Action (46)	“Doing something wrong is still way better than doing nothing at all!”
	Persistence (45)	“I just keep stumbling forward in the right direction.”
Maladaptive Coping Strategies (408): Strategies that provide short-term relief but do not contribute to long-term symptom resolution.	Avoidance (101)	“I ignore all messages and put off making plans.”
	Distraction (100)	“I make myself watch a movie just to distract myself.”
	Escapism (40)	“Dreaming, or at least attempting to, brings me some comfort.”
Interpersonal Difficulties (402): Perceived challenges and struggles in interpersonal interactions, including loneliness, misunderstanding, and conflict.	Comparison with Others (66)	“And seeing others succeed reminds me of what I could have been if I were smarter, better looking, or luckier.”
	Loneliness (58)	“I was lonely for so many years.”
	Misunderstanding (30)	“People just don’t get what we feel on a daily basis.”
Work and Academic Performance (370): Aspects related to professional and academic settings.	Work (158)	“I quit my job when they started giving me attitude.”
	Academic Performance (102)	“My grades have suffered so badly.”
	Productivity (29)	“I always preferred starting work first thing in the morning.”
Trauma and Abuse (272): Experiences of trauma and abuse.	Trauma (89)	“Life is tough for most people. Some are lucky and live great lives without trauma, while others have to learn how to live again and rise above their past.”
	Abuse (56)	“Currently facing daily abuse at work for making humanitarian choices.”
	Trigger (55)	“I know you mentioned crying randomly, but in those moments, pause, breathe deeply, and listen to your body and mind. Is something triggering you?”
Lifestyle and Routine (245): Includes daily life aspects such as lifestyle routines and sleep hygiene.	Sleeping (45)	“I decided not to set an alarm this morning so I could catch up on sleep.”
	Routine (42)	“I do all my chores and housework on Saturday”
	Pet Companionship and Wellbeing (21)	“I have pets and I can’t stand the thought of leaving them behind.”
Negative Interpersonal Experiences (234): Experiences of adverse social interactions.	Feelings of Inadequacy and Neglect (44)	“I feel like I’m not doing enough for my family, even though I do everything I can.”
	Invalidation (41)	“People think we can just snap out of it.”
	Toxic Behavior (21)	“They were acting really toxic and immature.”
Cognitive Functions and Impairments (187): Perceived decreases in cognitive functioning.	Memory (36)	“If it’s something important, I forget immediately. If it’s unimportant, I remember every detail.”
	Attention Impairment and Cognitive Decline (34)	“I can’t focus anymore. I read something ten times and still can’t understand.”
	Memory Impairment (30)	“I can’t remember anything anymore.”
Suicidal Thoughts and Self-harm (187): Experiences and mentions of suicidal ideation and self-harm.	Suicidal Ideation with Desire to End Life (112)	“[It feels like there’s been a fire burning in your mind for years that you can’t put out, and it hurts and makes you panic] I’m just ready to end it all.”
	Thoughts of Suicide and Death (26)	“I think about suicide at least once a day.”
	Self-Harm (17)	“I have a history of self-harm and suicidal thoughts.”
Fatigue and Energy (182): Issues related to low energy levels.	Tiredness and Exhaustion (107)	“I’d sit at home doing nothing and still feel completely exhausted.”
	Fatigue (36)	“It sometimes feels like my brain is operating at 5%, like a computer trying to boot up but the system needs more resources.”
	Energy Depletion and Lack of Energy (32)	“It is frustrating when you don’t have the energy to do anything.”
Physical Health Practices (182): Practices for maintaining physical health like nutrition and hygiene.	Weight and Food Choices (41)	“Keep a water bottle and a jar of peanut butter for nutrition (unless you have a nut allergy) in your room.”
	Nutrition and Supplements (36)	“The healthier your regular diet is, the less you’ll crave junk food. Empty calories just make you crave more.”
	Hygiene (30)	“Try to keep your space tidy and take a shower at least once a day.”
Life Transitions (150): Includes major life changes.	Responsibilities (44)	“All my bills and responsibilities were taken care of.”
	Parental Responsibility (26)	“You should totally fight for shared custody if you want to be involved in your child’s life.”
	Loss of a Loved One (23)	“My dad was my best friend, and he passed away about a year ago.”
Support-seeking Behaviors (142): Actions to seek help and support as well as reaching out.	Seeking Help (67)	“You need to talk to your teacher, parent, or another trusted adult. What you’re describing isn’t healthy, and you can get help for this.”
	Invitation to Connect (17)	“Feel free to send me a message or we can keep talking here.”
	Reaching Out (12)	“Props to you for reaching out ^_^”
Financial Issues (121): Concerns related to financial stability.	Financial Strain and Hardship (47)	“I am the main caretaker and a single income family and it is tough.”
	Cost Reduction (25)	“You could easily hire someone to do the same job for $7 less per hour.”
	Financial Stability and Freedom (13)	“I was fortunate to keep my decent job through this year.”
Emotional Regulation (120): Processes of managing and regulating emotions.	Emotional Impact (28)	“Yesterday I started crying as soon as I got off the phone with her because my feelings were hurt.”
	Emotional Burden (24)	“There are some patients who are so far gone that he loses sleep, waiting for the call.”
	Mood Swings (17)	“I had a crying fit for at least 20 minutes, and it wasn’t for any reason other than being ‘sad’ in the bipolar way.”
Creative Expression (115): Creative activities like hobbies and journaling.	Creativity and Self-Expression (34)	“Go and create something and get your creativity stimulated.”
	Hobbies (29)	“I found knitting to be a fun way to pass the time.”
	Reading and Writing (26)	“I have been reading philosophy, mostly stoicism.”
Substance Abuse (74): Mentions of substance (ab)use.	Addiction (20)	“But then I quickly build tolerance and have to keep increasing the dose, which soon turns into addiction.”
	Substance Use (17)	“Sadly, I turn to drinking to break out of my bubble.”
	Caffeine Consumption (16)	“I keep telling myself to switch to green tea with less caffeine, but I just love the taste of coffee too much.”

### 3.1 Instillation of hope

**Figure 4:**
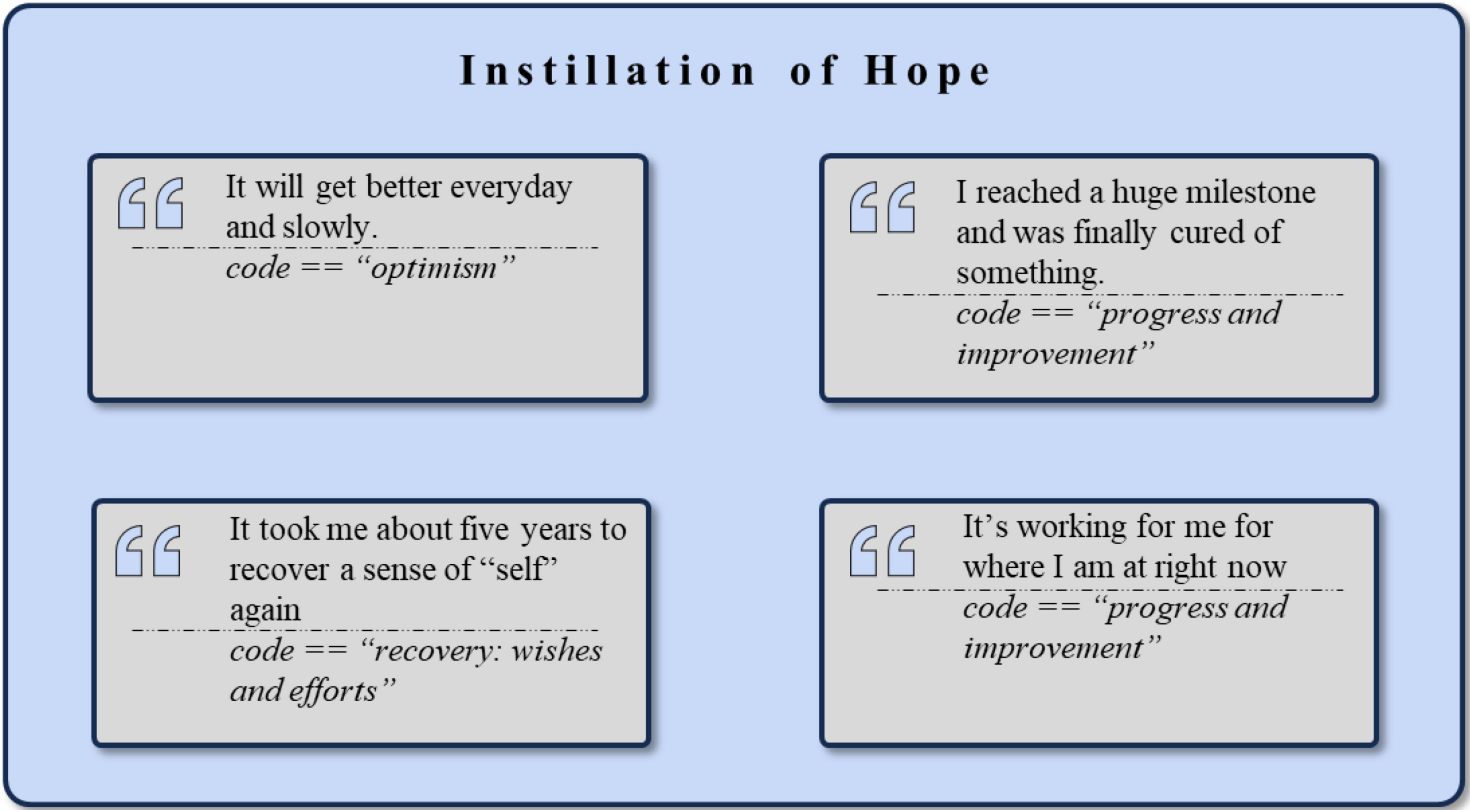
Exemplary rephrased quotes and *codes* assigned to the theme *Instillation of Hope*.

The extracted community discussions exhibited various interactions that illustrate the factor of *Instillation of Hope*, with example quotes displayed in **Figure 4**. Within the *Positive Attitudes and Character Traits* cluster (n = 1515), the frequent codes of *Hope* (n = 235), *Perspective* (n = 67), and *Resilience* (n = 50) demonstrate how group members continually encourage one another that things can get better. This high frequency of positive statements indicates a community deeply committed to fostering a hopeful outlook. Also, the community engages in uplifting and empathetic conversational strategies, as shown by the frequent mentions of *Gratitude* (n = 300), *Appreciation* (n = 246), and *Kindness* (n = 72). This mutual reinforcement also sets a standard for others to emulate, as seen in the cumulative emphasis on *Strength* (n = 55), *Empowerment* (n = 31), and *Perseverance* (n = 36).

In the *Personal Growth and Development* cluster (n = 837), the journey of self-improvement and processes related to recovery are shared by the users. Codes such as *Progress and Improvement* (n = 180), *Personal Growth* (n = 47), *Learning* (n = 64) and *Finding Meaning* (n = 28) highlight the ongoing development of individuals as they confront and overcome challenges. The discrepancies between the desire for improvement and the difficulties faced are evident in the frequent mentions of *Recovery: Wishes and Efforts* (n = 74), *Overcoming Challenges* (n = 28), and *Practice* (n = 25).

The *Motivation and Goal-setting* cluster (n = 446) shows how users share how they motivate themselves and set daily goals. Dominant codes such as *Motivation* (n = 163), *(Taking) Action* (n = 46), and *Goal Setting* (n = 37) demonstrate how community members are spurred into action. The *Desire for Change* (n = 29) and *Desire for Normalcy* (n = 29) reveal a wish to return to or establish a state of normalcy and stability. Similarly, *Personal and Persistent Effort* (n = 19), *Planning* (n = 19), and *Anticipation* (n = 18) reflect members’ proactive recovery strategies, emphasizing planning and positive action. These shared approaches highlight a belief in achievable recovery, creating an optimistic outlook in the community. Stories of recovery and growth, such as *Embracing the Healing Process* (n = 18), *Learning from Mistakes* (n = 15), *Transformation* (n = 12), and *Moving Forward* (n = 15), serve as sources of inspiration, guiding others toward self-improvement.

### 3.2 Universality (of Suffering)

**Figure 5:**
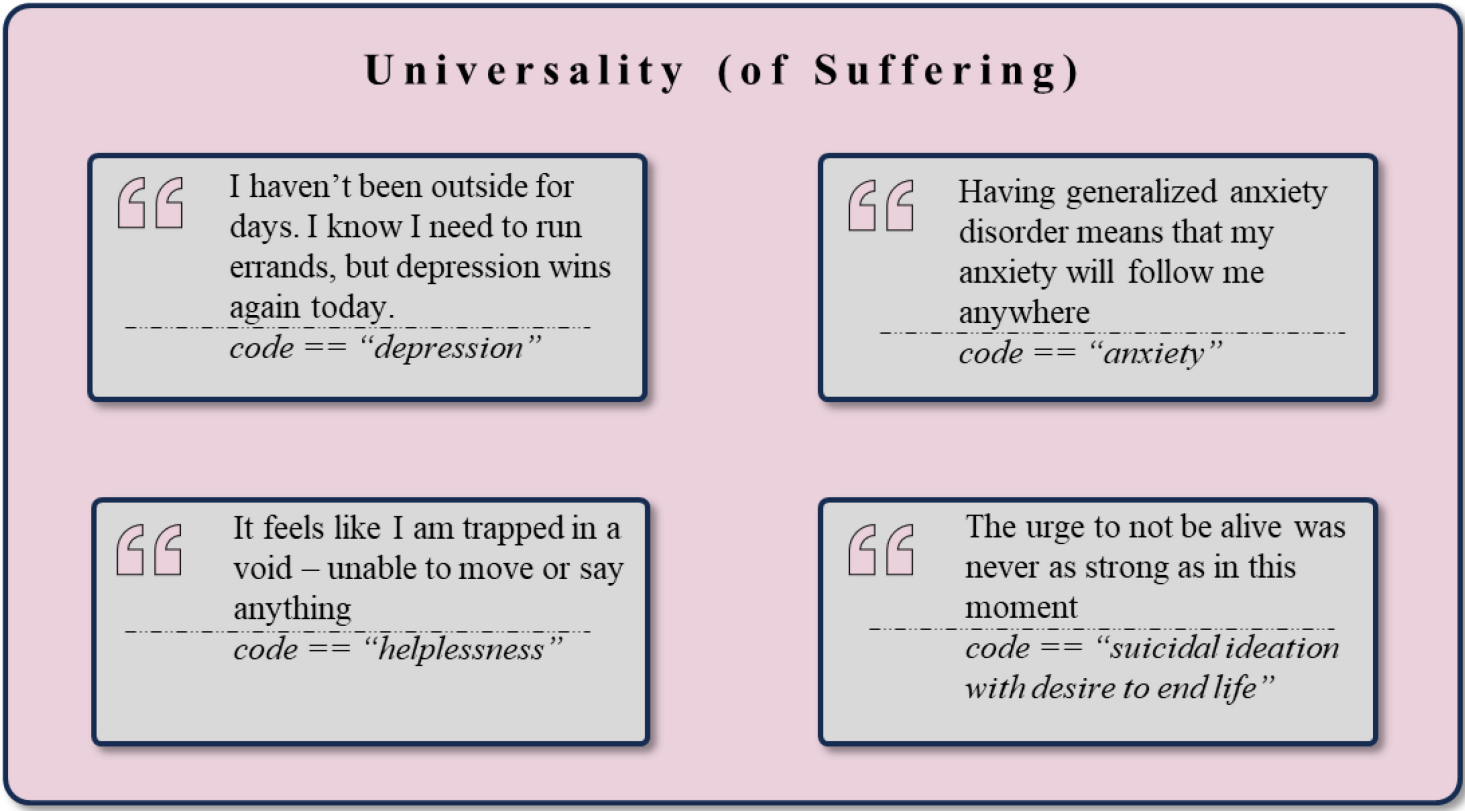
Exemplary rephrased quotes and *codes* assigned to the theme *Universality (of Suffering)*.

One of the central aspects of the analyzed data revolves around the universality of human suffering, specifically reflecting syndrome and symptom-level experiences, with example quotes illustrated in **Figure 5**. The most frequent extracted cluster is *Anxiety and Fear-related Constructs/Symptoms* (n = 1,686), showcasing several manifestations and symptoms of anxiety disorders such as *Anxiety* (n = 584), *Stress* (n = 284), *Fear* (n = 258), and *Panic Attack* (n = 113). This cluster also includes anxiety-related symptoms like *Feeling Overwhelmed* (n = 62), *Overthinking and Indecisiveness* (n = 42), *Worry* (n = 30), and *Rumination* (n = 28).

Another large cluster that maps to the theme of *Universality of Suffering* is *Depression and Depressive Symptoms* (n = 1,175). Within this cluster, *Depression* (n = 308) is the most frequently mentioned code, indicating experiences of low mood and depressive states. This cluster further encompasses symptoms constitutive of depressive disorders, such as *Negative Thoughts* (n = 131), *Sadness and Grief* (n = 62), *Guilt* (n = 57), *Regret* (n = 47), and *Apathy* (n = 37). In addition, a sub-cluster *Suicidal Thoughts and Self-harm* (n = 187) aligns with the overarching cluster of *Depression and Depressive Symptoms*. It reveals the severe suffering of some individuals, highlighted by the occurrences of *Suicidal Ideation with a Desire to End Life* (n = 112), followed by *Thoughts of Suicide and Death* (n = 26), and *Self-Harm* (n = 17).

The *Universality of Suffering* is also mirrored in the *Physical Health and Symptoms* cluster (n = 553), which captures physical health-related issues like *Sleep Issues* (n = 76), *Symptoms* (n = 56), *Physical Illness* (n = 55), and *Nausea* (n = 25). These physical health concerns underscore the interconnectedness of physical and mental health. The *Trauma and Abuse* cluster (n = 272) reflects users’ experiences with *Trauma* (n = 89), *Abuse* (n = 56), *PTSD* (n = 24), and recurring *Triggers* (n = 55). The narrative of suffering extends beyond emotional and psychological symptoms, as evidenced by the *Cognitive Functions and Impairments* cluster (n = 187), which highlights issues such as *Attention Impairment and Cognitive Decline* (n = 34) and *Memory Impairment* (n = 30). Fatigue and energy-related issues are evident in the *Fatigue and Energy* cluster (n = 182), with *Tiredness and Exhaustion* (n = 107), *Fatigue* (n = 36), and general *Energy Depletion* (n = 32) being common codes. Substance use concerns, found in the *Substance Use* cluster (n = 74), highlight worries about *Addiction* (n = 20), *Smoking and Cessation* (n = 11), and *Heavy Drinking and Alcoholism* (n = 10).

### 3.3 Awareness of relational impact

**Figure 6:**
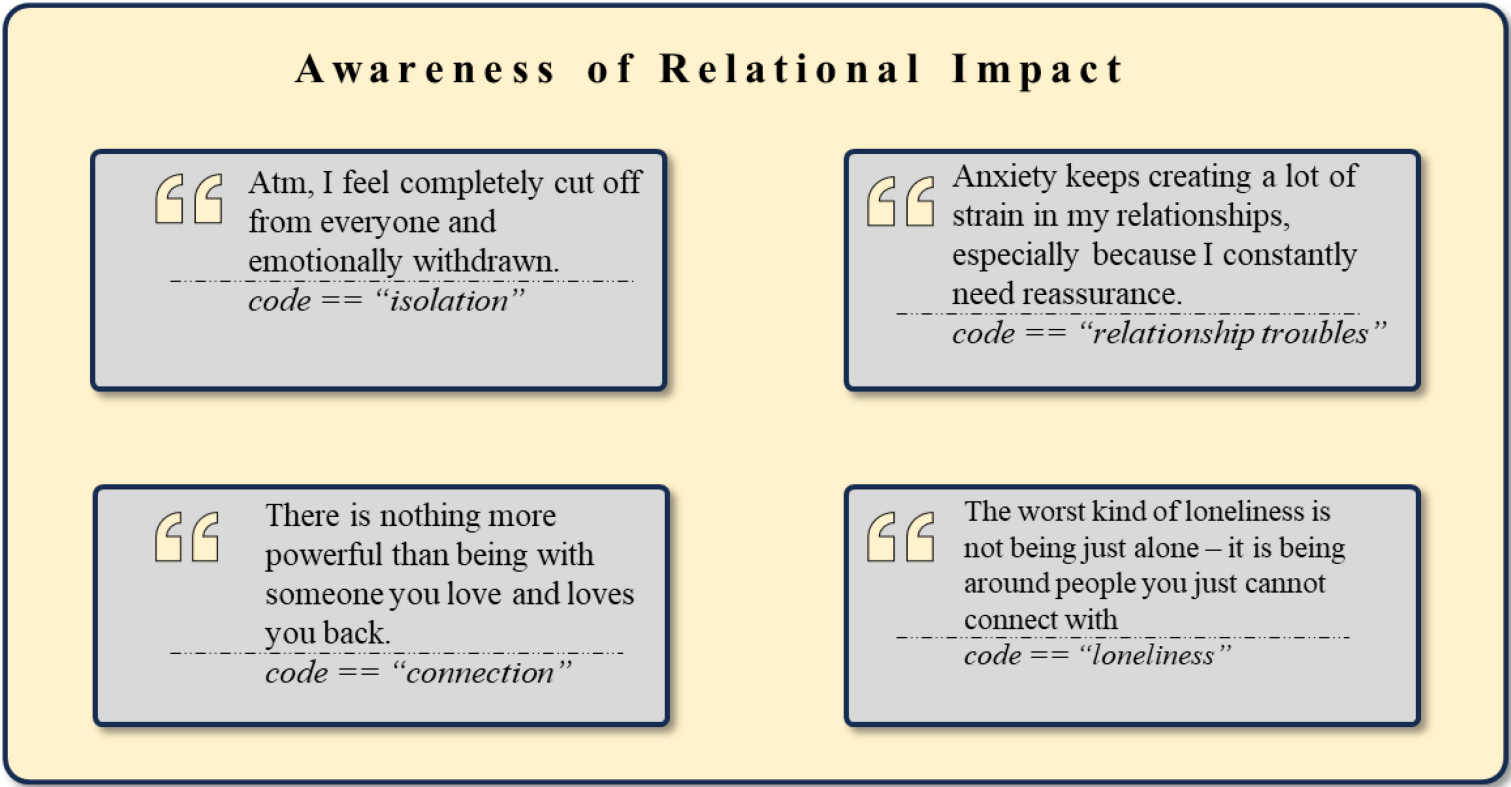
Exemplary rephrased quotes and *codes* assigned to the theme *Awareness of Relational Impact*.

The theme *Awareness of Relational Impact* was identified within the data, with illustrative example quotes provided in **Figure 6**. The cluster *Relationships and Social Interactions* (n = 738) provides an overview of more positively annotated relationships. Codes such as *Affection and Love* (n = 158) demonstrate the importance of close bonds and acts of showing affection. *Solitude* (n = 148) reflects on the state of preferring to be alone, while *Connection* (n = 95) and *Relationships* (n = 69) underscore the necessity of meaningful and lasting social bonds. The *Value of Friendship* (n = 41), *Forgiveness and Reconciliation* (n = 20), *Trust* (n = 19), as well as *Belonging* (n = 16) further highlight the importance of supportive relationships.

The following clusters emphasize a more negative view on social relationships. The cluster *Interpersonal* Difficulties (n = 402) reveals common relationship issues, with *Comparison with Others* (n = 66) being the most frequent, indicating a tendency to measure oneself against peers, often accompanied by feelings of inadequacy. Similarly, *Envy* (n = 21) further underscores this tendency. *Loneliness* (n = 58), *Loss of Relationships and Friends* (n = 22), and *Conflict* (n = 22) are other associated codes, reflecting a pervasive sense of ongoing isolation and interpersonal struggle. Other codes demonstrating the perceived lack of social support include *Misunderstanding* (n = 30), *Lack of Support* (n = 28), and *Unsupportive and Dysfunctional Parenting* (n = 14).

*Negative Interpersonal Experiences* (n = 234) capture more severe social adversities, often based in stigma. For instance, *Feelings of Inadequacy and Neglect* (n = 44) indicate profound social and emotional neglect. *Invalidation* (n = 41) highlights the detrimental effects of having one’s feelings or identity dismissed. Additionally, *Toxic Behavior* (n = 21), *Stereotyping and Discrimination* (n = 19), *Bullying* (n = 19), *Blaming the Victim* (n = 12), as well as *Gaslighting* (n = 9) illustrate hostile social interactions with harmful effects on one’s psychosocial health.

### 3.4 Imparting of information

**Figure 7:**
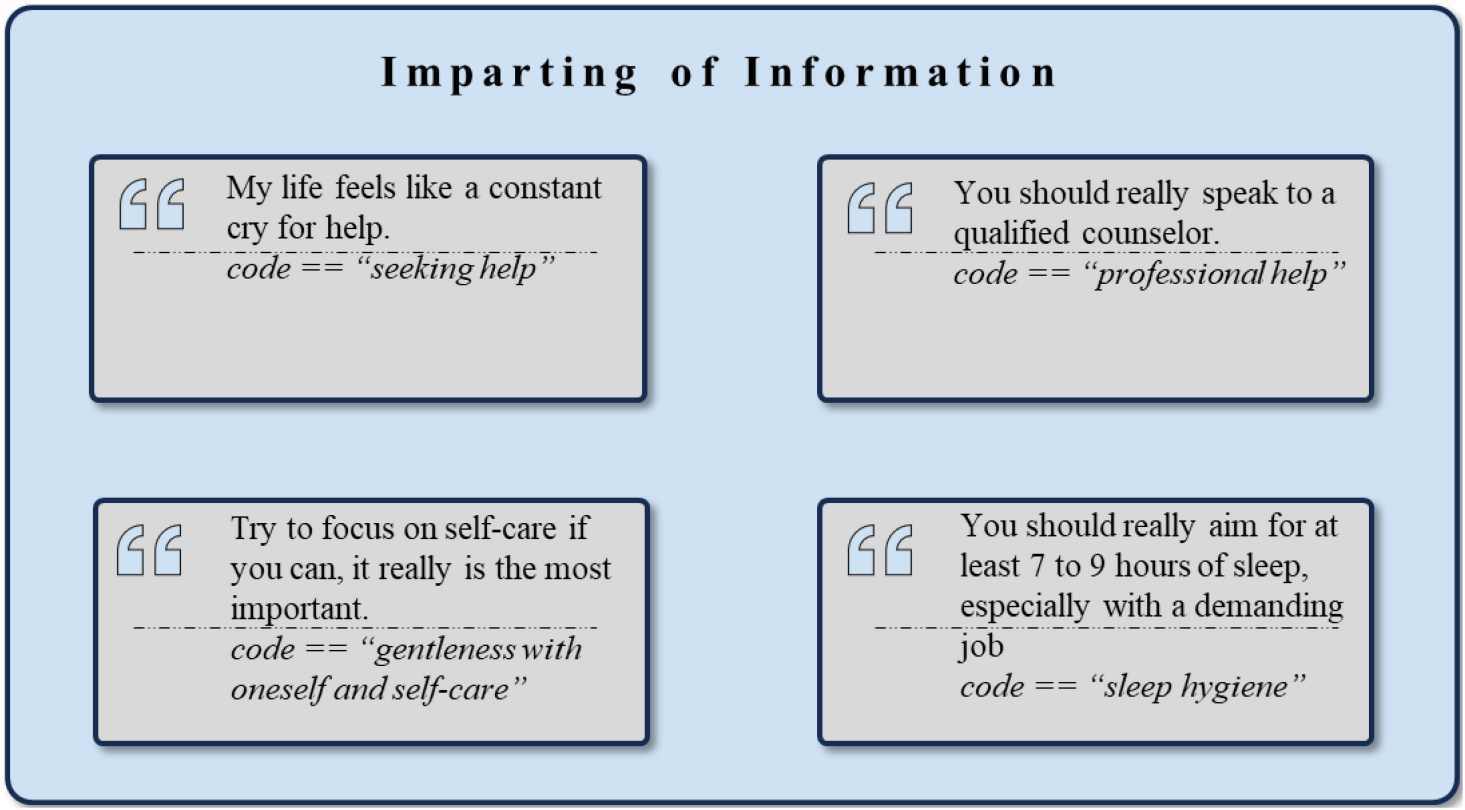
Exemplary rephrased quotes and *codes* assigned to the theme *Imparting of Information*.

The therapeutic factor, *Imparting of Information*, was a central component reflected in the dataset, with illustrative example quotes presented in **Figure 7**. The cluster of *Adaptive Coping Strategies* (n = 1297) demonstrates concrete and practical techniques from the users for managing distress and negative feelings. Prominent strategies include *Acceptance* (n = 157), *Relaxation* (n = 147), *Physical Exercise and Activity* (n = 137), and *Gentleness with Oneself and Self-care* (n = 136). Additional practices deemed effective such as *Mindfulness* (n = 78), *Breathing Exercises* (n = 65), and *Meditation* (n = 55) are also frequently mentioned. Taking *Things at One’s Own Pace* (n = 39) emphasizes the importance of self-regulation and planning as core components of effective coping strategies. Furthermore, cognitive techniques like *Positive Self-Talk* (n = 31), *Grounding Techniques* (n = 19), *Resource Utilization* (n = 13), and *Normalization* (n = 13) underscore the use of cognitive restructuring. *Self-Protection* (n = 28), *Problem-solving* (n = 14), *Sleep Hygiene* (n = 27), and *Hydrotherapy (or Taking a Shower)* (n = 18) indicate the diverse range of approaches individuals utilize to manage distress.

A sub-group of *Adaptive Coping Strategies* is the *Creative Expression* cluster (n = 115). Activities such as *Enjoying Music* (n = 22) and *Engaging in Activities* (n = 36) offer relief from negative emotional states. Engaging in reflective practices such as *Journaling* (n = 23), *Reading and Writing* (n = 26), and *Creative Hobbies* (n = 29) are further suggested by the users to facilitate emotional processes.

Another sub-group of *Adaptive Coping Strategies* is the cluster of *Physical Health Practices* (n = 182). It primarily emphasizes maintaining physical health. The cluster primarily emphasizes maintaining physical health with recommendations that focus on *Weight and Food Choices* (n = 41), *Nutrition and Supplements* (n = 36), and *Hygiene* (n = 30), thereby offering practical advice on leading a balanced and healthy life. Conversely, the cluster of *Maladaptive Coping Strategies* (n = 408) highlights how certain behavioral responses to stress may provide temporary relief but ultimately prove counterproductive. The most common strategies include *Avoidance* (n = 101), *Distraction* (n = 100), *Escapism* (n = 40), *Procrastination* (n = 34), and *Detachment* (n = 19). This categorization reflects both the authors’ interpretation and users’ explicit statements, describing how certain behaviors led to further negative outcomes.

The cluster of *Mental Health and Treatment* (n = 921) provides extensive information on experiences with therapy, medication, and treatment options. Key topics discussed include professional *Help and Therapy* (n = 219), *Medication* (n = 180), *Mental Health Awareness* (n = 89), and general treatment processes such as *Treatment* (n = 34), *Hospitalization* (n = 27), *Therapy and Counseling* (n = 25), as well as *Consulting Healthcare Professionals* (n = 24).

The cluster *Lifestyle and Routine* (n = 245) is centered around daily living and habits, with topics including *Sleeping* (n = 45) and *Routine* (n = 42). Moreover, the *Support-seeking Behaviors* (n = 142) cluster primarily involves information-sharing activities, where individuals actively seek advice and support. Common mentions within this cluster include *Seeking Help* (n = 67) and making *Invitations to Connect* (n = 17). The cluster for *Work and Academic Performance* (n = 370) highlights the importance of balancing professional and educational commitments, emphasizing *Work* (n = 105), *Academic Performance* (n = 158), *Productivity* (n = 29), and *Managing Job Satisfaction* (n = 11). Finally, *Financial Issues* (n = 121) pertains to managing economic stressors. Other discussions focus on *Financial strain and Hardship* (n = 47) and *Cost Reduction* (n = 25), providing practical tips for achieving financial stability.

### 3.5 Altruism

**Figure 8:**
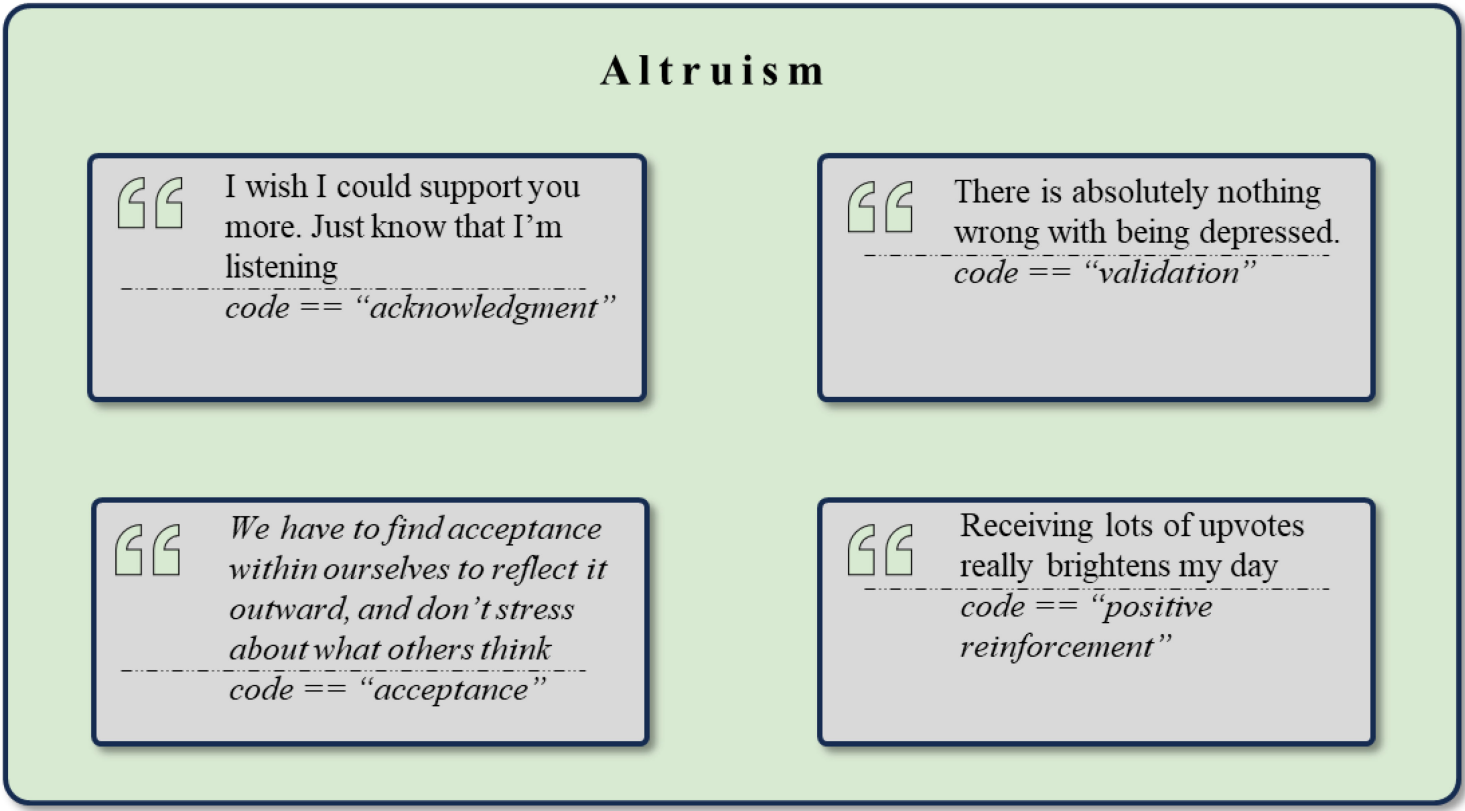
Exemplary rephrased quotes and *codes* assigned to the theme *Altruism*.

*Support and Encouragement* (n = 3313) was the most frequent cluster, highlighting a deeply supportive environment that aligns with Yalom’s factor of *Altruism* (**Figure 8**). *Social Support* (n = 619) was the most prevalent code, reflecting numerous instances of community members offering empathy, listening, and helpful advice. *Encouragement* (n = 501) reveals posts where members uplift and motivate each other with words of affirmation and morale-boosting messages. *Well Wishes* (n = 252) illustrate the community’s tendency to share heartfelt sentiments for others’ success, health, and happiness. Moreover, the code *Empathy* (n = 245) shows moments of genuine understanding and compassion. *Acknowledgement* (n = 243) and *Understanding* (n = 229) further reveal how members validate each other’s experiences, fostering a supportive environment where individuals feel seen and heard. *Reassurance* (n = 169) provides comfort and alleviates anxieties, while *Validation* (n = 134) reinforces others’ sense of worth and positive self-regard. *Positive Reinforcement* (n = 106), *Recognition* (n = 81), and *Affirmation* (n = 74) further enhance the supportive atmosphere. Additionally, the altruistic nature of the online communities is further demonstrated by the *Adaptive Coping Strategies* cluster, where users frequently share personal experiences and successful coping strategies to help one another. Similarly, the *Positive Attitudes and Character Traits* cluster further emphasizes *Hope*, *Gratitude*, and *Resilience*, leading to a narrative of positive outlook and perspective.

### 3.6 Group Cohesion

**Figure 9:**
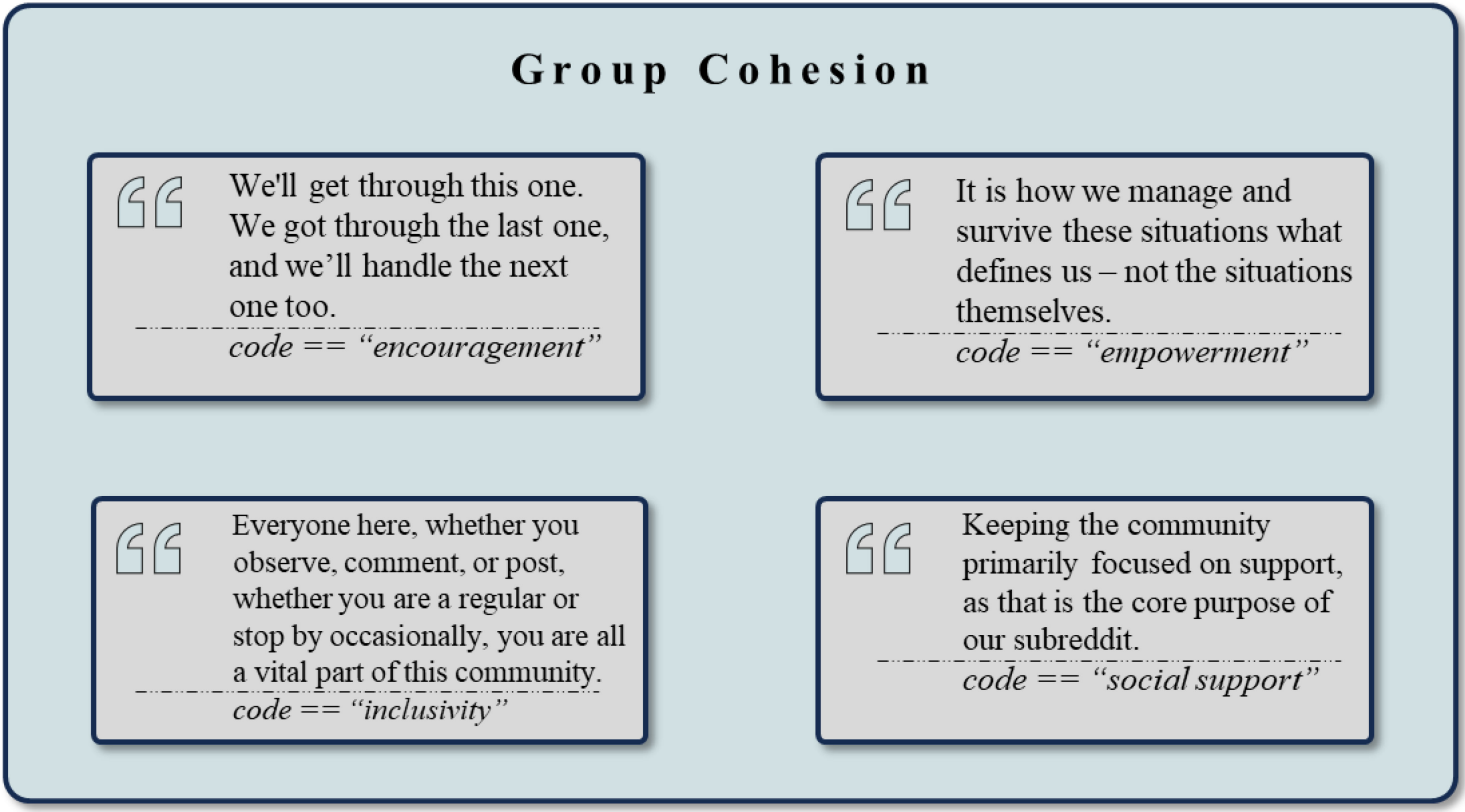
Exemplary rephrased quotes and *codes* assigned to the theme *Group Cohesion*.

Several clusters were associated with the therapeutic process of *Group Cohesion*, with illustrative example quotes in **Figure 9**. Most directly, the cluster *Emotional Expression and Communication* (n = 1173) shows that users create a supportive, open, and like-minded environment. Highly frequent codes such as *Sharing Feelings* (n = 262) and *Sharing of Experiences* (n = 116) suggest the group’s readiness to open up and express vulnerabilities. Furthermore, the group’s communication is reflected in codes such as *Communication* (n = 113), *Concern* (n = 95), and *Emotional Response* (n = 43), which highlight the emphasis on open dialogue.

The factor of *Group Cohesion* is further indirectly shown in the highly frequent and dominant cluster of *Support and Encouragement* (n = 3313), highlighting the members’ approaches to offer encouraging messages and social as well as emotional support to each other.

Although not directly linked to *Group Cohesion* in the sense of explicit statements about perceiving oneself as part of the group, the *Positive Attitudes and Character Traits* cluster adds another layer to this theme. The frequent occurrence of *Gratitude* (n = 300), *Appreciation* (n = 246), and *Hope* (n = 235) shows respect and empathy, creating a welcoming and open atmosphere.

### 3.7 Catharsis

**Figure 10:**
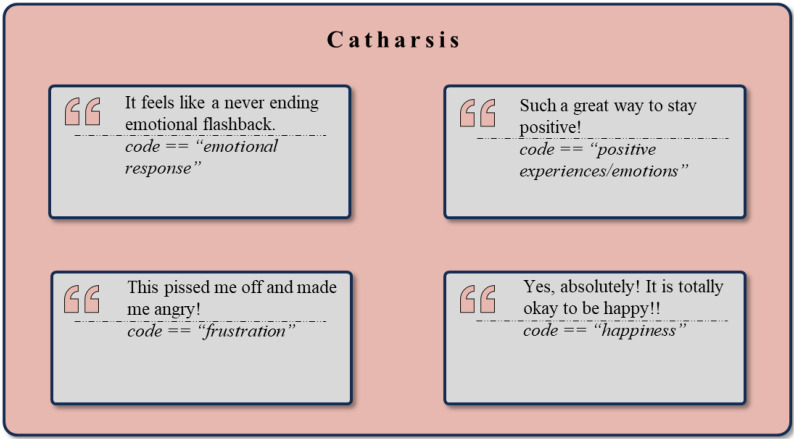
Exemplary rephrased quotes and *codes* assigned to the theme *Catharsis*.

In the analyzed Reddit data, the factor of Catharsis (as described by Yalom) was identified through the expression and release of emotional tension, arising from both positive and negative emotions (19). Supporting example quotes are shown in **Figure 10**. The *Positive Emotions and Experiences* cluster (n = 1297), which includes *Happiness* (n = 161), *Optimism* (n = 101), and *Pride* (n = 80), represents a vital aspect of the emotional spectrum where cathartic release can play a crucial role. Furthermore, *Achievements* (n = 170) and *Relief* (n = 60) demonstrate the active presence of positive emotional experiences that often follow meaningful emotional expression and release. Other illustrative examples from this cluster include *Celebrations* (n = 61), *Enjoyment* (n = 54), *Excitement* (n = 48), and *Engagement* (n = 39).

Conversely, the cluster of *Negative Emotions and Experiences* (n = 1258), encompassing *Frustration* (n = 131), *Pain* (n = 104), and *Anger* (n = 63), illustrates the emotional burdens that many individuals experience, often characterized by intense internal strife. Examples within this cluster also include *Struggles* (n = 135), *Criticism* (n = 43), *Judgment* (n = 22), *D*istress (n = 14), and *Feelings of Rejection* (n = 11):

The *Emotional Regulation* cluster (n = 120), consisting of aspects such as *Mood Swings* (n = 17) and *Emotional Burden* (n = 24), highlights the process of managing emotions. Further illustrative examples include the *Desire for Calmness* (n = 15), *Mood Management and Improvement* (n = 10), response to *Aggression* (n = 9), and *Inner Balance* (n = 7).

### 3.8 Existential factors

**Figure 11:**
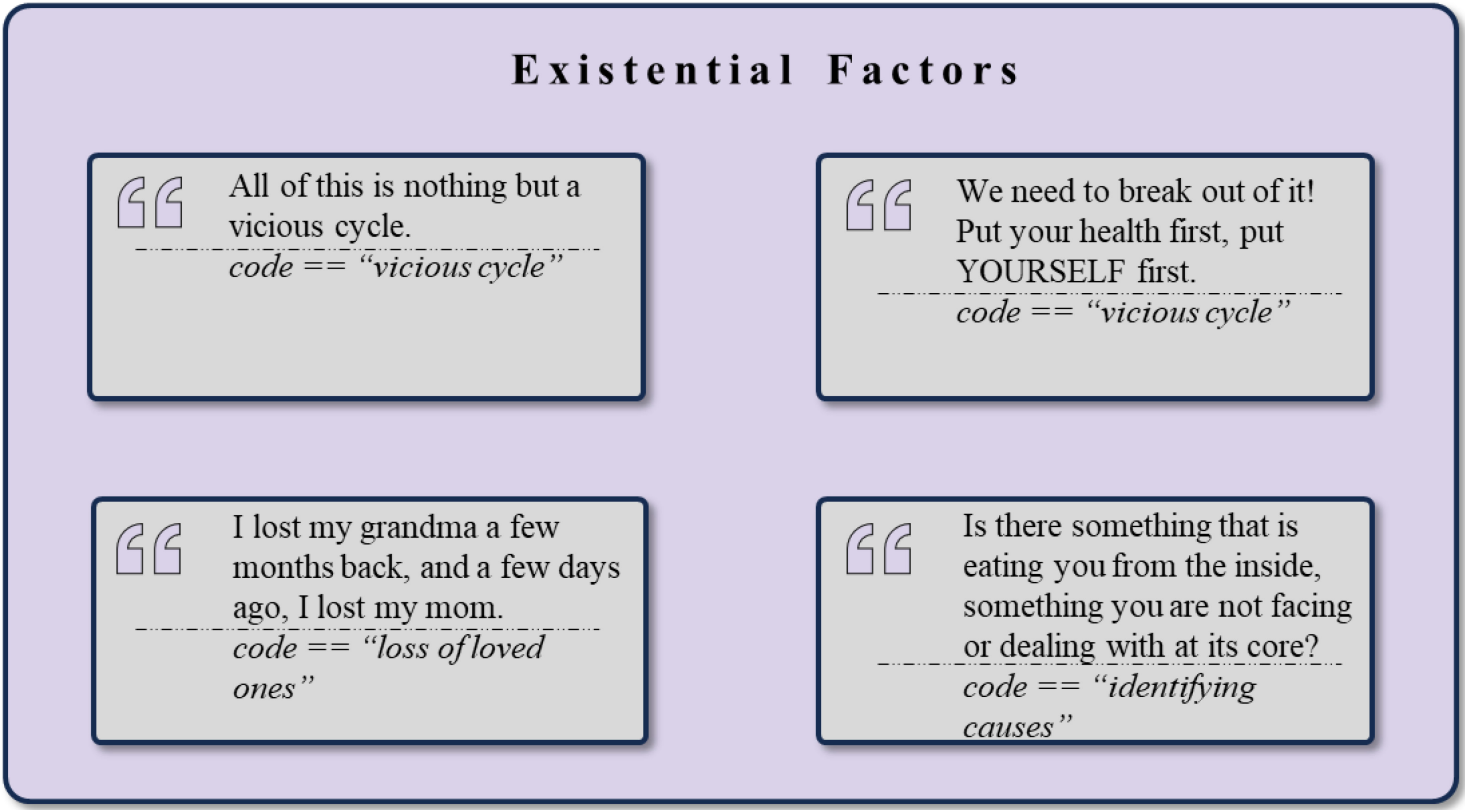
Exemplary rephrased quotes and *codes* assigned to the theme *Existential Factors*.

The therapeutic process, *Existential Factors*, was also represented in the dataset, highlighting themes of self-reflection, identity development, and navigating life transitions, with example quotes demonstrated in **Figure 11**. The cluster of *Self-reflection and Analysis* (n = 514) underscores the importance of gaining insight through emotional expression. This cluster includes instances of *Self-Reflection* (n = 71), *Uncertainty* (n = 67), *Questioning* (n = 45), and *Childhood Memories* (n = 27) where individuals engage in introspection to understand their behaviors. Other notable codes are *Realization* (n = 38), *Behavioral Norms and Misconduct* (n = 28), *Sensitivity* (n = 25), *Self-Evaluation* (n = 20), *Existential Questioning* (n = 15), and *Decision-Making Processes* (n = 9).

The *Self-Perception and Identity* cluster (n = 502) reveals the development of a sense of identity within the group. Key elements of this cluster include issues of *Expectations* (n = 72), *Self-Worth* (n = 41) and *Self-Perception* (n = 35), as individuals grapple with their self-image and esteem. The cluster also encompasses *Identity* (n = 29), highlighting how group interactions enable personal identity exploration. Concepts like *Independence and Autonomy* (n = 29), *Internal Conflict* (n = 18), and *Comfort Zone* (n = 14) are relevant as they reflect the internal struggles individuals face when defining themselves within a social context. More negative aspects are exhibited, such as *Battling with a Negative Self-Image* (n = 33), undergoing *Self-Criticism* (n = 31), experiencing *Insecurity* (n = 22), feeling the need to *Wear a Mask and not Being Able to Be Oneself* (n = 18), and facing a *Loss of Self and Identity* (n = 16).

*Life Transitions* (n = 150) exemplify how individuals navigate significant life changes, reflected in themes such as *Responsibility* (n = 44), *Parental Responsibility* (n = 26), *Loss of a Loved One* (n = 23), *Relocation* (n = 12), and *Divorce and Separation* (n = 6) that indicate the broader spectrum of life changes.

## 4 Discussion

In this study, we explored how user‐generated discourse in mental health‐focused Reddit communities can be interpreted through the lens of Yalom’s group therapeutic factors (19). Although our approach cannot infer actual therapeutic processes due to the lack of real-time interactions, professional facilitation, and direct observation, our interpretative findings suggest that eight of Yalom’s factors (e.g., *Instillation of Hope*, *Group Cohesion*, *Altruism*) are present in this asynchronous, peer-led environment. By employing a mixed‐methods approach—including an LLM for coding, cosine similarity/BERTopic for topic clustering, and iterative human validation—we were able to generate 31 qualitative clusters and map them to eight group therapeutic themes with direct textual evidence (**Figure 3**).

A large portion of the community interactions was mapped to *Instillation of Hope*, as reflected by frequent codes such as *Social Support*, *Encouragement*, *Hope*, and *Gratitude*. This indicates an empowering sense of collectivity. These dynamics underscore how peer‐led environments can foster forward‐thinking mindsets (19, 20) and illustrate the significance of role modeling in online mental health forums (70). Mirroring prior findings (17, 71), peer encouragement also appears to help individuals adopt proactive coping strategies.

Similarly, *Group Cohesion* is suggested by clusters that show emotional expression, open communication, and a shared sense of communal belonging—key elements Yalom emphasizes in face‐to‐face group therapy (19). While real‐time interactions and nonverbal cues are absent, repeated exchanges of empathy and vulnerability appear to produce a digital solidarity effect. Consistent with Diefenbeck et al. (20), cohesive group mechanisms can indeed materialize in asynchronous contexts, although the structural limitations (e.g., delayed feedback, limited social cues) naturally constrain some interpersonal processes (72). Nonetheless, empathy and warmth appear contagious, reinforcing cycles of supportive engagement (73, 74).

In these digital spaces, *Altruism* emerges through hands‐on coping tips—such as mindfulness exercises (75), physical activity (76), or evidence‐based daily routines (39)—thus helping peers navigate distress and potentially empowering them to self‐manage symptoms. Altruistic acts, including listening, affirming, and offering detailed advice, benefit both the giver and receiver, aligning with research that highlights the prosocial rewards of helping others online (77, 78). In parallel, a recurring theme of *Imparting of Information*—which covered everything from medication experiences to financial or academic guidance—demonstrates how these forums function as peer-managed hands-on knowledge hubs. This resonates with the interdependence of Yalom’s factors, whereby *Altruism* and *Group Cohesion* are strengthened through concrete, helpful exchanges, spurring further community involvement (10, 19).

While many subreddit interactions were coded as encouraging, some parts addressed distressing symptoms, reflecting the factor of *Universality*. Users shared personal experiences of anxiety, depression, suicidal ideation, self‐harm, and other struggles—underlining that suffering is a common human experience (79). Such disclosures can be double‐edged: on the one hand, recognizing that others share similar hardships may reduce isolation and self‐stigma (11, 24). On the other hand, exposure to distressing details might intensify a contagion effect, potentially escalating harmful behaviors (80, 81). Anonymity and user‐led dynamics can make moderation challenging, especially around high‐risk topics like suicidality or self‐harm (18, 22). The platform’s accessible, hyperpersonal nature (7) also draws individuals who feel misunderstood by offline networks (35). This highlights a paradox: while supportive interactions can help mitigate isolation, unregulated content could possibly amplify negative emotional spirals or enable phenomena such as “toxic positivity”—a phenomenon where overly optimistic messages fail to acknowledge a user’s deeper distress (74, 82).

Another key factor identified from the data was *Catharsis*, closely tied to emotional regulation (19, 36). Posts reflecting both *Positive Emotions and Experiences* (e.g., celebrating achievements) and *Negative Emotions and Experiences* (e.g., expressions of anger or despair) suggest that releasing emotional tension is a central function of these forums. Although cathartic exchanges can be validating and therapeutic, they also risk exposing users to explicit, unmoderated descriptions of self‐harm or suicidal intentions (83). As with Universality, the benefits of free emotional expression must be weighed against the possibility of exacerbating vulnerability among already distressed individuals (84, 85).

The theme *Existential Factors* appeared in user discussions on self-reflection, identity development, and life transitions, focusing on introspection and the search for meaning. These peer-led communities can serve as hyperpersonal spaces (7), enabling reflected communication of both positive and negative feelings while emphasizing self-reflection rather than idolizing others (86). This environment promotes self-reflective dialogues akin to those outlined by Tiidenberg et al. (87), allowing users to engage in self-reflection and receive feedback, which may improve health outcomes (80, 86).

Consistent with the findings of Diefenbeck et al. (20), the factors revolving around social learning were mostly absent or rare. *Interpersonal Learning*, *Corrective Recapitulation of Family of Origin*, *Imitative Behavior*, and *Development of Socialization Techniques* were less evident, likely due to the peer-led and asynchronous nature of the online communities. Yalom suggests that factors like *Interpersonal Learning* and *Development of Socialization Techniques* require professional facilitation and in-person interactions, which are difficult to replicate online due to limitations such as lack of immediate feedback and non-verbal cues (19, 72). Though social learning is limited in the data, there are reflections and indicators of *Awareness of Relational Impact*. Users processed relational dynamics and reflected on their social relationships with all their strengths, challenges, and adversities.

From a methodological standpoint, our study shows how a human‐in‐the‐loop pipeline combining LLMs and topic modeling can produce both breadth and interpretive depth in large‐scale qualitative analyses (47, 48, 50). By prompting GPT‐3.5‐turbo 16k to segment text into concise meaning units, high initial coding accuracy was achieved. Nonetheless, as prior work suggests (43, 44), purely algorithmic or machine-driven approaches may still miss or misinterpret subtle, context‐dependent nuances. Németh et al. (44) term this the “hermeneutic failure” of machine annotation: while algorithms can detect overt patterns, they struggle with deeper interpretive processes—especially for higher‐order sociological or existential concepts. To tackle this, we (1) used an LLM to generate semantically coherent codes in context, (2) required direct quotations for evidence, and (3) included several human validation steps.

### 4.1 Limitations of the study

Several limitations must be acknowledged in this study. Using Reddit as a data source may introduce biases because its predominantly younger, tech‐savvy user base is not fully representative of the broader population. In addition, approximately 10% of the initial codes were excluded by the topic model as noise, potentially omitting relevant information (see **Supplementary Appendix C** for details). Although a bottom-up approach offered a comprehensive view of potential categories, it sometimes made it difficult to capture all semantically relevant codes under one cluster. Nevertheless, it helped avoid the bias of pre-selecting categories. This approach, however, did help avoid the bias associated with pre-selecting categories. Another important limitation relates to the rapid pace of technological development in the field of LLMs. The specific model used in this study was deprecated prior to publication (68), implying that future replication efforts may require adapting the methodology to different or more advanced models. Furthermore, the study’s focus on top-rated comments may skew the findings toward more positively received interactions, potentially overlooking less popular yet equally important contributions. The cross-sectional and interpretative nature of the research design also restricts our ability to assess the actual therapeutic value of the interactions. Although a large portion of the interactions were positive or conveyed positive coded messages, the design does not allow for evaluating causal therapeutic effects. In this context, it was not possible to infer whether positive interactions were genuinely therapeutic or represented ‘toxic positivity’—where overly optimistic messages mask underlying distress without fostering authentic emotional growth. Finally, rather than calculating interrater reliability directly with the LLM, two authors evaluated its output. This procedure might have overestimated the precision metrics, as the LLM itself segments the raw data and produces the codes. However, given the large volume and complexity of the data, having human experts review the LLM’s consistently formatted output was a pragmatic and feasible approach.

### 4.2 Recommendations for future research

Future research should prioritize co-creation processes with actual users and forum administrators to ensure ethical integrity and adherence to participatory research principles, particularly when working with publicly accessible mental health data. In addition, studies could include data from a broader range of online platforms and integrate longitudinal data to enhance generalizability. Analyzing longitudinal data with state-of-the-art (reasoning) LLMs (e.g., OpenAI’s GPT-4o and o1/o3(-mini), Google’s Gemini 2.0, Anthropic’s Claude Sonnet 3.7, xAI’s Grok 3, and DeepSeek’s r1) could help illustrate symptom trajectories over time. Employing a top-down analysis with these models, which incorporate larger context windows, may also be able to identify actual therapeutic processes based on extensive codebooks and longitudinal data. Advances in capable LLMs could further automate the conversion of large qualitative corpora into structured formats, with findings then integrated into a real-time interactive web application developed in collaboration with forum administrators and aligned with community guidelines for identifying positive and therapeutic interactions while also automating moderation of harmful content.

Another approach, illustrated in **Supplementary Appendix G**, is a fully automated workflow that replicates all proposed steps. It orchestrates multiple (advanced reasoning) LLMs to handle data cleaning, code generation, codebook maintenance, clustering, and thematic analysis—while still allowing optional human validation. Moreover, integrating ethnographic methods can further enhance this approach. For example, an online ethnography (88) found that lay narratives in depression forums act as “secular ritual healing,” reframing distress, suggesting that combining digital coding with fieldwork may offer a more holistic view of therapeutic dynamics.

## 5 Conclusion

This study demonstrates how Yalom’s group therapeutic factors can be applied to large-scale, peer‐led online mental health discussions, using a mixed‐methods workflow that integrates NLP, large language models, and human supervision. This theoretical model offered a coherent basis for discourse analysis, allowing us to map factors such as *Instillation of Hope*, *Imparting of Information*, *Group Cohesion*, and *Altruism* across a large portion of the data. These online forums may represent important and resourceful spaces for individuals facing mental health challenges by offering continuous, peer-based support, even though the cross-sectional and interpretive design of our analyses captured both positive interactions and negative exchanges.

In this context, the socially reinforced (and sometimes contagious) nature of online interactions can amplify both positive and negative spirals, potentially affecting self-stigma and distress (22, 74, 82). While anonymity and the hyperpersonal nature of online communication may reduce stigma and facilitate self-disclosure, they also enable the unregulated spread of potentially harmful content. This is particularly relevant for suicide-related discussions, which may be associated with the Werther effect (23), and self-harm behaviors, which can be transmitted and normalized through online interactions (89). Due to these effects, there is a necessity for moderation in online support communities to prevent potential harm while preserving the benefits of peer-organized mental health discussions (90).

Overall, many of Yalom’s therapeutic factors could be mapped to the data, highlighting the feasibility of this theoretical framework for interpreting online interactions. The findings further hint at the potential of self-organized digital spaces to enable self-organized therapeutic processes, but they also point to unresolved challenges regarding the regulation and ethical oversight of these communities. As mental health issues continue to rise and access to professional treatment remains limited (91), future research might expand data collection across diverse online platforms and employ longitudinal designs. Including community members could further inform the development of machine-driven strategies for peer-led mental health interventions.

## Data Availability

The original contributions presented in the study are included in the article/**Supplementary Material**, further inquiries can be directed to the corresponding author/s.
