# Supplementary material for: Identifying Yalom’s group therapeutic factors in anonymous mental health discussions on Reddit: a mixed-methods analysis using large language models, topic modeling and human supervision
Source: Front Psychiatry. 2025 Jun 9;16:1503427. doi: 10.3389/fpsyt.2025.1503427 (PMC12183517; doi:10.3389/fpsyt.2025.1503427)
Supplement: Supplementary file 1 [file DataSheet1.zip › Appendix B.docx]

Appendix B

All validated topics and sub-topics. The number in brackets indicates the total frequency count for each topic/code.

Adaptive Coping Strategies (1297):

Acceptance (157)

Relaxation (147)

Physical Exercise and Activity (137)

Gentleness with Oneself and Self-Care (136)

Coping Mechanism (106)

Mindfulness (78)

Breathing (65)

Meditation (55)

Taking it at Your Own Pace (39)

Engaging in Activities (36)

Positive Self-Talk (31)

Self-Protection (28)

Breaks (28)

Sleep Hygiene (27)

Enjoying Music (22)

Grounding Technique (19)

Hydrotherapy (Taking a Shower) (18)

Problem-Solving (14)

Nighttime as a Safe Haven (14)

Resource Utilization (13)

Normalization (13)

Embracing Imperfection (13)

Prayer (12)

Reframing (12)

Safe Space (10)

Self-Reliance (10)

Self-Talk (10)

Sensory Focus (9)

Preference for Silence (7)

Serotonin and Dopamine Management (7)

Letting Go (7)

Treating Self and Others Well (6)

Tension Release (5)

Chunking (3)

Planning (3)

Anxiety and Fear-related Constructs/Symptoms (1686):

Anxiety (584)

Stress (284)

Fear (258)

Panic Attack (113)

Feeling Overwhelmed (62)

Overthinking and Indecisiveness (42)

Worry (30)

Rumination (28)

Intrusive Thoughts (27)

Disassociation (24)

OCD (23)

Fear of Failure (21)

Emotional Distress (20)

Paralysis (14)

Hypervigilance (13)

Fear of Judgment (13)

Dread (12)

Fear of Death (11)

Fight or Flight Response (11)

Specific Phobia (11)

Distorted Thinking (11)

Perceived Danger (10)

Sense of Doom (10)

Caution (8)

Rumination and Intrusive Thoughts (8)

Brain Fog (7)

Nervousness (7)

Depersonalization (6)

Magnification (5)

Perception of Death (5)

Preoccupation (5)

Shortness of Breath (3)

Cognitive Functions and Impairments (187):

Memory (36)

Attention Impairment and Cognitive Decline (34)

Memory Impairment (30)

Concentration Issues (18)

General Focus and Concentration (15)

Forgetfulness (14)

Selective Memory (14)

Cognitive Techniques (10)

Brain Functioning (7)

Lack of Clarity (6)

Lack of Knowledge and Insight (3)

Creative Expression (115):

Creativity and Self-Expression (34)

Hobbies (29)

Reading and Writing (26)

Journaling (23)

Humor (3)

Depression and Depressive Symptoms (1175):

Depression (308)

Negative Thoughts (131)

Sadness and Grief (62)

Guilt (57)

Suffering (55)

Crying (51)

(Self-)Doubt (49)

Regret (47)

Apathy (41)

Numbness and Emptiness (40)

Hopelessness (37)

Hate and Self-Loathing (30)

Despair and Desperation (29)

Shame (25)

Bipolar Disorder (25)

(Self-)Blame (25)

Lack of Purpose (23)

Low Self-Esteem (20)

Nostalgia (20)

Feeling Worthless and Terrible (18)

Loss of Interest (17)

Helplessness (13)

Mania (12)

Self-Perceived Failure (11)

Loss of Quality of Life (10)

Giving Up (7)

Feeling Lost and Confused (7)

Pessimism (5)

Emotional Expression and Communication (1173):

Sharing Feelings (262)

Impact (176)

Personal Sharing of Experiences (116)

Communication (113)

Concern (95)

Emotional Response (43)

Listening (38)

Apology (30)

Agreement (24)

Understanding and Recognizing Emotions (24)

Inquiry (23)

Vulnerability (23)

Reminder (19)

Surprise (19)

Report (19)

Opening Up (18)

Future Perspective (18)

Emotional State (18)

Emotional Expression (18)

Expressing Feelings (11)

Metaphors and Analogies (11)

Ambivalence (9)

Mixed Feelings (9)

Emotional and Spiritual Resonance (9)

Preference for Directness (8)

Feeling Understood (8)

Expressing and Recognizing Needs (6)

Clarification (6)

Emotional Regulation (120):

Emotional Impact (28)

Emotional Burden (24)

Mood Swings (17)

Desire for Calmness (15)

Mood Management and Improvement (10)

Response to Aggression (9)

Inner Balance (7)

Self-Regulation (5)

Emotional Processing and Management (5)

Fatigue and Energy (182):

Tiredness and Exhaustion (107)

Fatigue (36)

Energy Depletion and Lack of Energy (32)

Burnout (7)

Financial Issues (121):

Financial Strain and Hardship (47)

Cost Reduction (25)

Financial Stability and Freedom (13)

Savings (12)

Financial Management (10)

Broke (6)

Affordability (4)

Wealth (4)

Interpersonal Difficulties (402):

Comparison with Others (66)

Loneliness (58)

Misunderstanding (30)

Lack of Support (28)

Misinterpretation (24)

Loss of Relationships and Friends (22)

Conflict (22)

Envy (21)

Unsupportive and Dysfunctional Parenting (14)

Betrayal (14)

Heartbreak (13)

Family Conflicts and Challenges (12)

Lack of Social Skills (11)

Invisibility (10)

Conversation Troubles (9)

Relationship Troubles (8)

Inability to Relate (7)

Avoiding Interactions (7)

Confrontation (6)

Questioning Intent (6)

Inability to Explain (5)

Trust Issues (5)

Toxic Communities and Individuals (4)

Life Transitions (150):

Responsibilities (44)

Parental Responsibility (26)

Loss of a Loved One (23)

Relocation and Moving Out (12)

Adoption (11)

Life Changes and Phases (11)

Life Challenges and Adjustments (11)

Divorce and Separation (6)

Adulthood Challenges and Transitions (6)

Lifestyle and Routine (245):

Sleeping (45)

Routine (42)

Pet Companionship and Wellbeing (21)

Healthy and Active Lifestyle (14)

Exploring Alternatives (14)

Lifestyle Change (13)

Work-Life Balance (13)

Energy Management (13)

Beauty (11)

Simplicity and Minimalism (11)

Preference for Home Environment (11)

Cleaning Efforts (11)

Caffeine Reduction (7)

Organization (5)

Scheduling (4)

Outdoor Activities (4)

Vacation (3)

Grocery Shopping (3)

Maladaptive Coping Strategies (408):

Avoidance (101)

Distraction (100)

Escapism (40)

Procrastination (34)

Sarcasm (21)

Detachment (19)

Inactivity (18)

Watching TV (16)

Withdrawal (13)

Denial (13)

Avoidance of Social Situations (12)

Minimization (10)

Rationalization (6)

Pretending (5)

Mental Health and Treatment (921):

Professional Help and Therapy (219)

Medication (180)

Mental Health (89)

Awareness (57)

Diagnosis (37)

Treatment (34)

Stigma (28)

Hospitalization (27)

Therapy and Counseling (25)

Consulting an Healthcare Professional (24)

Antidepressants (22)

Effectiveness Variability (22)

Intervention (20)

Advocacy (13)

Misconceptions (12)

Genetic Predisposition (12)

Research (12)

Symptom Management (12)

Therapist Relations and Competence (11)

System/Biological Imbalance (11)

Side Effects (11)

Cognitive Behavioral Therapy (CBT) (11)

Psychiatric Consultation (6)

Healthcare Access and Options (6)

Health Insurance Coverage (6)

Role of Psychiatrists (5)

Online Resources (3)

Limited Effectiveness (3)

Educating Others (3)

Miscellaneous (452):

Nature (50)

Weather Preferences (45)

Social Media (27)

Age (23)

Dream (19)

Rule Enforcement and Compliance (18)

Gaming and Movies (16)

Repetition (15)

Significance (15)

Frequency (14)

Inevitability and Unavailability (12)

Association (11)

Content Sharing (11)

Limited Media Consumption (10)

Contextual Focus (9)

Anonymity (9)

Perception of Colors (7)

Animal Behavior and Traits (7)

Humor (6)

Necessity (6)

Reddit (6)

Relevance (6)

Cat-Specific Interests (6)

Seriousness (5)

Clothing (5)

Gender (5)

Involuntary (5)

Structure (5)

Unpleasant Environments (5)

Emoticon (4)

Family History (4)

Justice (4)

Lack of Context (4)

Preparedness (4)

Role Reversal (4)

Taste (4)

Verification (4)

Deterrent (3)

Noise (3)

Prevalence (3)

Patterns of Failure (3)

Temporary Solutions (3)

Thread Lock (3)

Missed Opportunities (3)

Lack of Improvement (3)

Innocence (3)

Generational Differences (3)

Updates (3)

Disability (3)

Variety (3)

Perceived Lack of Choice (3)

Motivation and Goal-setting (446):

Motivation (163)

(Taking) Action (46)

Persistence (45)

Goal Setting (37)

Desire for Change (29)

Personal and Persistent Effort (19)

Planning (19)

Anticipation (18)

Desire for Normalcy (16)

Desire for Normalcy and Peace (13)

Consistency (12)

Commitment (11)

Willingness to Try (10)

Hard Work (8)

Negative Emotions and Experiences (1258):

Difficulty (279)

Struggles (135)

Frustration (131)

Pain (104)

Anger (63)

Pressure (59)

Discomfort (43)

Criticism (43)

Disappointment (30)

Confusion (26)

Dislike (24)

Feelings of Embarrassment (23)

Judgment (22)

Worsening Situation (21)

Lack of Control (21)

Negative Emotion (19)

Discontent (17)

Bad Days (15)

Distress (14)

Feeling Trapped (14)

Negative Pandemic Impact (12)

Breakdown (12)

Boredom (11)

Feelings of Rejection (11)

Negative Response (10)

Longing (10)

Lack of Solutions (10)

Alienation (9)

Selfishness (8)

Negativity and Bias (7)

Perceived Injustice (7)

Misery (7)

Feeling Misunderstood (7)

Thoughts of Revenge (6)

Negative View of Holidays (5)

Negative Consequences (5)

Feeling Hurt (5)

Aggression and Violence (4)

Negative Outlook (3)

Negative Memories (3)

Cynicism (3)

Negative Interpersonal Experiences (234):

Feelings of Inadequacy and Neglect (44)

Invalidation (41)

Toxic Behavior (21)

Stereotyping and Discrimination (19)

Bullying (19)

Blaming the Victim (12)

Dismissiveness (12)

Negative Influences (9)

Gaslighting (9)

Insensitivity (8)

Accusation (8)

Manipulation (8)

Negative Feedback (7)

Dismissive Attitude (5)

Intimidation (3)

Living for Others (3)

Rudeness (3)

Unfairness (3)

Personal Growth and Development (837):

Progress and Improvement (180)

Recovery: Wishes and Efforts (74)

Learning (64)

Survival (53)

Personal Growth (47)

Adaptation (30)

Importance of Education (30)

Overall Well-being (28)

Overcoming Challenges (28)

Finding Meaning (28)

Visualization (27)

Practice (25)

Potential (24)

Passion and Exploration (22)

Stagnation (21)

Healing Process (18)

Effectiveness (15)

Embracing Mistakes for Growth and Learning (15)

Moving Forward (15)

Transformation (12)

Giftedness (11)

Self Investment (11)

Sobriety and Recovery (11)

Long Journey and Individual Path (10)

Mistakes (8)

Improved Quality of Life (7)

Mental State Improvement and Shifts (7)

Self-Realization (6)

Maturity and Growth (5)

Overcoming Negativity (5)

Physical Health Practices (182):

Weight and Food Choices (41)

Nutrition and Supplements (36)

Hygiene (30)

Meal Preparation and Food Preferences (29)

Staying Hydrated (18)

Eating Habits (17)

Weight (11)

Physical Health and Symptoms (553):

Sleep Issues and Difficulties (76)

Symptoms (56)

Physical Illness (55)

Physical Symptoms (41)

Unpleasant Body Sensations (27)

Nausea (25)

Jaw Clenching and Dental Issues (24)

Stomach Issues (23)

Activity Limitations and Challenges (21)

Physical Health (20)

Headache (19)

Rapid Heart Palpitations (17)

Muscle Tension and Spasms (15)

Medical Tests (14)

Medical Conditions (14)

Physical Sensations (12)

Physical Injuries/Traumas (10)

COVID-19 (10)

Physical Stress Symptoms (9)

Appetite Loss (9)

Accident (9)

Physical Contact (8)

Immune System Suppression (8)

Heart Conditions and Issues (8)

Difficulties with Basic Needs (7)

Physical Sensation (5)

Dizziness (5)

Cough (3)

Psychosomatic Symptoms (3)

Positive Attitudes and Character Traits (1515):

Gratitude (300)

Appreciation (246)

Hope (235)

Kindness (72)

Control (70)

Perspective (67)

Value (61)

Strength (55)

Resilience (50)

Caring for Others (44)

Humor (43)

Perseverance (36)

Empowerment (31)

Respect (26)

Boundaries (25)

Patience (25)

Privacy (23)

Authenticity (18)

Courage and Bravery (13)

Honesty (12)

Value and Importance of Life (10)

Personal and Family Prioritizing (8)

Memory Preservation and Honoring (7)

Leadership (6)

Inclusivity (6)

Nonjudgmental Approach (5)

Positive Attitude (5)

Standing Up for Oneself (5)

Willingness to Protect Others (4)

Intelligence (4)

Expertise (3)

Positive Emotions and Experiences (1297):

Positive Experiences/Emotions (181)

Achievements and Success (170)

Happiness (161)

Optimism (101)

Pride (80)

Celebration (61)

Relief (60)

Enjoyment (54)

Excitement (48)

Interest (40)

Comfort (40)

Engagement (39)

Admiration (31)

Small Victories (27)

Confidence (26)

Joy (26)

Benefits (21)

Contentment and Peacefulness (20)

Peacefulness and Contentment (20)

Stability (16)

Laughter (15)

Celebrating Success (14)

Freedom (14)

Satisfaction and Self-fulfillment (14)

Gladness (6)

Luck (5)

Liberation (4)

Feeling Good (3)

Relationships and Social Interaction (738):

Affection and Love (158)

Solitude (148)

Connection (95)

Relationships (69)

Value of Friendship (41)

Social Interaction (30)

Family Perception (24)

Forgiveness and Reconciliation (20)

Community (20)

Trust (19)

Dependency (18)

Belonging (16)

Companionship (13)

Social Influence (10)

Desire for Friendship (10)

Collaboration (10)

Finding Balance in Relationships (9)

Engaging in Activities (8)

Reciprocity (7)

Desire for Companionship (7)

Initiating Conversations (6)

Self-perception and Identity (502):

Expectations (72)

Self-Worth (41)

Self-Perception (35)

Negative Self-Image (33)

Self Criticism (31)

Identity (29)

Independence and Autonomy (29)

Identification (28)

Insecurity (22)

Approval (20)

Self-Awareness (19)

Internal Conflict (18)

Wearing a Mask (Not being able to be oneself) (18)

Individual Differences (18)

Loss of Self and Identity (16)

Self-Discovery (14)

Comfort Zone (14)

Struggles with Self-Worth (12)

Change in Sense of Self (7)

Self-Esteem Boosting (5)

Imposter Syndrome (5)

Deserving (5)

Body Image (4)

Uniqueness (4)

Role Consideration (3)

Self-reflection and Analysis (514):

(Self-)Reflection (71)

Uncertainty (67)

Questioning (45)

Realization (38)

Behavioral Norms and Misconduct (28)

Childhood Memories (27)

Sensitivity (25)

Consequences (22)

Observation (21)

Self-Evaluation (20)

Behavior Analysis and Responses (16)

Questioning Existence (15)

Analyzing and Managing Thoughts (15)

Behavioral Analysis and Responses (11)

Vicious Cycle (11)

Identifying Causes (10)

Recognizing Limitations (9)

Decision-Making Process (9)

Children's Potential and Behavior (9)

Personality Traits and Reflection (8)

Contradiction (7)

Feeling Out of Place (7)

Difficulty in Performing Tasks (7)

Problem Perception and Dynamics (5)

Self-Fulfilling Prophecy (4)

Lack of Awareness (4)

Existential Crisis (3)

Substance Use (74):

Addiction (20)

Substance Use (17)

Caffeine Consumption (16)

Smoking and Cessation (11)

Heavy Drinking and Alcoholism (10)

Suicidal Thoughts and Self-harm (187):

Suicidal Ideation with Desire to End Life (112)

Thoughts of Suicide and Death (26)

Self-Harm (17)

Desire for Non-Existence (10)

Suicide Attempts (9)

Suicide Prevention (8)

Suicide Impacts and Relations (5)

Support and Encouragement (3313):

Social Support (619)

Encouragement (501)

Well Wishes (252)

Empathy (245)

Acknowledgment (243)

Understanding (229)

Reassurance (169)

Validation (134)

Positive Reinforcement (106)

Recognition (81)

Affirmation (74)

Celebrating Others' Success (70)

Suggestions and Recommendations (68)

Sympathy (67)

Congratulations (54)

Compassion (50)

Advice (41)

Solidarity (38)

Safety (37)

Compliment (35)

Offering Help (35)

Praise (30)

Suggestion (28)

Assurance (20)

Availability (16)

Acknowledging Others' Struggles (15)

Comforting Gesture (13)

Condolences (13)

Offer for Conversation (9)

Celebrating Other’s Success (7)

Empathetic Response (3)

Good Vibes (3)

Stay Strong (3)

Welcoming (3)

Uplifting (2)

Support-seeking Behaviors (142):

Seeking Help (67)

Invitation to Connect (17)

Reaching Out (12)

Seeking Attention (11)

Seeking and Exploring Solutions (11)

Seeking Guidance (11)

Seeking Comfort (8)

Lack of Advice (5)

Time-related Constructs (227):

Time (114)

Prioritization (29)

Time Management (28)

Duration (18)

Perception of Time (14)

Temporary (11)

Urgency (7)

Sense of Waiting (6)

Trauma and Abuse (272):

Trauma (89)

Abuse (56)

Trigger (55)

Neglect and Abandonment (38)

PTSD (24)

Nightmares (7)

Near-Death Experience (3)

Work and Academic Performance (370):

Work (158)

Academic Performance (102)

Productivity (29)

Task Management (25)

Career Change (13)

Employment (13)

Job Dissatisfaction (11)

Job Search and Management (9)

Career Development and Advancement (6)

Degree (4)
