# Supplementary material for: Identifying Yalom’s group therapeutic factors in anonymous mental health discussions on Reddit: a mixed-methods analysis using large language models, topic modeling and human supervision
Source: Front Psychiatry. 2025 Jun 9;16:1503427. doi: 10.3389/fpsyt.2025.1503427 (PMC12183517; doi:10.3389/fpsyt.2025.1503427)
Supplement: Supplementary file 1 [file DataSheet1.zip › Appendix C.docx]

Appendix C

All excluded topics and codes. The number in brackets indicates the total frequency count for each topic/code.

-----------------------------------------------------------------------------------------------------------------------------------------------------------------------------------------------------------------------------------------------------------------------------------------------------------------------------------------------------------------------------------------------------------------------------------------------------------------------------------------------------------------------------------------------------------------------------------------------------------------------------------------------------------------------------------------------------------------------------------------------------------------------------------------------------------------------

Remainder (2930):

Accessibility (2)

Account Deletion (2)

Acting Normal (2)

Aromatherapy (2)

Asthma (2)

Atheism (2)

Audience (2)

Autopilot (2)

Avoiding Substance Use (2)

Being Prepared (2)

Belief in Afterlife (2)

Belief in Capability (2)

Belief in Others (2)

Belief in Self (2)

Bigger Picture (2)

Blessings (2)

Book Title (2)

Brain Dump (2)

Bucket List (2)

Buddhism (2)

Carefree Attitude (2)

Catch-22 Situation (2)

Celebratory Exclamations (2)

Challenges of Treatment (2)

Challenging Conversations (2)

Character Assessment (2)

Checking on Others (2)

Clean Environment (2)

Colorblindness (2)

Comfort in Familiarity (2)

Concealing Emotions (2)

Condemnation (2)

Conflict of Desires (2)

Consumerism (2)

Convincing Oneself (2)

Counteraction (2)

Customer Interaction (2)

Dark (2)

Decluttering (2)

Defeat (2)

Delayed Gratification (2)

Delegating (2)

Dental Care (2)

Depressive Realism (2)

Derealization (2)

Desire for Distance (2)

Desire for Peace (2)

Desire to Learn (2)

Desire to Leave (2)

Dismissive Attitudes (2)

Disorientation (2)

Disregard of Feelings (2)

Documentation (2)

Embracing Failure (2)

Emergency Services (2)

Emotional Regulation (2)

Emotional Turmoil (2)

End of Relationship (2)

Escaping Reality (2)

External Perception (2)

External Resources (2)

Extreme Measures (2)

Extreme Reaction (2)

Fall (2)

Familiarity (2)

Family Activities (2)

Family as a Reason to Live (2)

Fawn Response (2)

Feeling Defective (2)

Feeling Down (2)

Feeling Ignored (2)

Feeling Undervalued (2)

Feeling Weak (2)

Feeling of Exclusion (2)

Feeling of Existence (2)

Feeling of Imprisonment (2)

Feeling of Insignificance (2)

Financial Gain (2)

Flexible Schedule (2)

Focus on Present (2)

Focus on the Present (2)

Fortunate (2)

Frugality (2)

Functional Impairment (2)

Future-oriented Thinking (2)

Golden Rule (2)

Grandiosity (2)

Graying Hair (2)

Hair Loss (2)

Hallucinations (2)

Height (2)

Homeownership (2)

Household Chores (2)

Hyperventilation (2)

Hypomania (2)

Ideal Self (2)

Impostor Syndrome (2)

Impulsive Behavior (2)

Impulsivity (2)

Inability to Enjoy (2)

Inability to Perform Tasks (2)

Inability to Relax (2)

Information Overload (2)

Intensity of Emotion (2)

Intent to Help (2)

Interview Failure (2)

Intrusiveness (2)

Job Termination (2)

Lack of Execution (2)

Lack of Goals (2)

Lack of Instruction (2)

Lack of Relaxation (2)

Lashing Out (2)

Lasting Impression (2)

Laying on the Floor (2)

Layoffs (2)

Legacy (2)

Limited Career Options (2)

Lockdown (2)

Locking the Post (2)

Locking the Thread (2)

Long Episode (2)

Long Shifts (2)

Long-Term Effects (2)

Long-term Plan (2)

Loss of Income (2)

Lurking (2)

Masturbation (2)

Materialism (2)

Media Representation (2)

Medical Help (2)

Mental Overload (2)

Mental Strain (2)

Miscommunication (2)

Miserable (2)

Mistrust (2)

Natural Remedies (2)

Need for Information (2)

Need for Open Conversations (2)

Negative Behaviors (2)

Negative Childhood (2)

Negative Judgement (2)

Negative Reactions (2)

New Opportunities (2)

No Personal Attacks (2)

Nonexistent (2)

Novelty (2)

Obsessive Thinking (2)

Obsessive Thoughts (2)

Overindulgence (2)

Overstimulation (2)

Parent-Child Bond (2)

Parental Sacrifice (2)

Perceived High (2)

Perceived Laziness (2)

Persistent Negative Outlook (2)

Personal Development (2)

Personal Insult (2)

Physical Changes (2)

Physical Reaction (2)

Positive Impression (2)

Positive Self-Assessment (2)

Potential Harm (2)

Pretense of Well-being (2)

Proactiveness (2)

Psychological Effects (2)

Psychosis Experience (2)

Quitting Other Substances (2)

Reasons for Living (2)

Reasons to Live (2)

Reduced Attention Span (2)

Reduced Creativity (2)

Reluctance to Help Seeking (2)

Reluctance to Leave (2)

Reluctance to Return (2)

Request for Information (2)

Revised Delivery Plan (2)

Risk of Overdose (2)

Running (2)

Screaming (2)

Seeking Information (2)

Seeking a Way Out (2)

Self-Comfort (2)

Self-Definition (2)

Self-Help Techniques (2)

Self-Punishment (2)

Self-Sufficiency (2)

Sense of Purposelessness (2)

Sense of Security (2)

Setbacks (2)

Shared Activities (2)

Showering (2)

Sibling Dynamics (2)

Similar Feelings (2)

Simplistic Solutions (2)

Soothing Effect (2)

Special Day (2)

Special Occasion (2)

Spontaneity (2)

Startle Response (2)

Stoicism (2)

Suicidal Thoughts/Ideation (2)

Sustainability (2)

Sweetness (2)

Text Analysis (2)

Tingling Sensation (2)

Tinnitus (2)

Traffic (2)

Uncommon (2)

Unexpected Experience (2)

Unexpected Plot (2)

Unpaid Overtime (2)

Unwanted Attention (2)

Use of Emojis (2)

Validating Emotions (2)

Validity of Posts (2)

Visual Impairment (2)

Welcoming Atmosphere (2)

/r/BackOnYourFeet (1)

/r/SWResources (1)

/r/assistance (1)

/r/dbtselfhelp (1)

/r/depression (1)

/r/mixednuts (1)

/r/stopselfharm (1)

/r/suicidewatch (1)

2015 Woes (1)

Abandonment and Unloved Feelings (1)

Abnormal Reactions (1)

Abrupt Negative Shift (1)

Abrupt Resolution (1)

Abrupt Service (1)

Absence Limitation (1)

Absorbing World Events (1)

Absurdity (1)

Abusive Behavior (1)

Accepting Posts (1)

Access to Programs (1)

Access to Toilet (1)

Accessibility Challenge (1)

Accessibility of Internet (1)

Accidental Spills (1)

Account Linking (1)

Accuracy in Order (1)

Achieving Mental State (1)

Achieving a Specific Relationship (1)

Acknowledging Feelings (1)

Acknowledging Imagination (1)

Acknowledging Internet's Scale (1)

Acknowledging Non-Normalcy (1)

Acknowledging Severity (1)

Acknowledging Uncomfortable Emotions (1)

Acknowledging World's Cruelty (1)

Acne Coverage (1)

Acne Scarring (1)

Acting Techniques (1)

Acting with Purpose (1)

Active Living (1)

Acupressure (1)

Acute Episodes (1)

Ad Placement (1)

Adapting to Increased Togetherness (1)

Addictive Entertainment (1)

Additional Items (1)

Additional Links (1)

Addressing Risks (1)

Adjusting Language for Audience (1)

Adjusting to Reality (1)

Adjustment to Loss (1)

Adrenaline Release (1)

Adrenaline Seeker (1)

Advantage of Professor’s Office Hours (1)

Adverse Reaction to Old Photos (1)

Advice for Talking (1)

Advising Against Web Search (1)

Advisory Warning (1)

Advocating for Custody (1)

Affordable Options (1)

Affordable Rent (1)

Age Discrepancy (1)

Age-appropriate Education (1)

Aging Out (1)

Agitation (1)

Agnosticism (1)

Air Not Breaking Through (1)

Airbnb in Scotland (1)

Alarm Failure (1)

Alarm Setting (1)

Alarm System Analogy (1)

Alcohol Intolerance (1)

Alien Companion (1)

Alignment of Methods (1)

Allegation Response (1)

Allowing Emotions (1)

Alternative to Incarceration (1)

Ambience Interrupted (1)

Ambiguity of Feelings (1)

Ambiguous Mood (1)

Amusing Incident (1)

Amusing Thought (1)

Animal as a Healer (1)

Answering Questions (1)

Antacids (1)

Anthropomorphism (1)

Anti Drink and Drive (1)

Anti-Homophobic Stance (1)

Anticipating Negative Events (1)

Anticipating Reactions (1)

Anticipation for Cyberpunk 2077 (1)

Antistress Website (1)

Apartment Issue (1)

Apartment Rental (1)

Apathy towards Hobbies (1)

App Usage Patterns (1)

App-Blocking as a Coping Strategy (1)

Applicability of Strategy (1)

Application Sabotage (1)

Approachability (1)

Arai-guma (1)

Art Deal (1)

Asking Doctor for Help (1)

Asking for Opportunities (1)

Asking for Refund (1)

Associating Examples with Codes (1)

Association of Memories with Places (1)

Assumed Care in Personal Life (1)

Assumed Native Language (1)

Assuming Capacity (1)

Assuming Normalcy (1)

Assumption of Assistance (1)

Assumption of Help (1)

Assumptions based on Low Wage (1)

Assumptions of Entitlement (1)

Attaching a Reason (1)

Attachment Check (1)

Attachment Issues (1)

Attachment to Career (1)

Attempt Resolution (1)

Attempted Crankiness (1)

Attempted Rationalisation (1)

Attempting to Resolve (1)

Attempts to Help (1)

Attributing Emotion to Non-living (1)

Attribution of Reason (1)

Attribution to Laziness (1)

Audible Cringing (1)

Audience Attraction (1)

Automatic (1)

Automatic Response (1)

Available Accommodations (1)

Aversion to Reading (1)

Avoid Unsolicited Discussions (1)

Avoidance of Impacting Others (1)

Avoidance of Pity (1)

Avoiding Added Sugar (1)

Avoiding All-or-Nothing Thinking (1)

Avoiding Blanket Statements (1)

Avoiding Chaos (1)

Avoiding Doomscrolling (1)

Avoiding Eye Contact (1)

Avoiding Immediate Reactions (1)

Avoiding Loss (1)

Avoiding Mainstream (1)

Avoiding Manipulation (1)

Avoiding Overexertion (1)

Avoiding Political Discussions (1)

Avoiding Socializing (1)

Avoiding Stereotypes (1)

Avoiding Temptation (1)

Avoiding Toxic Comments (1)

Avoiding Uncomfortable Situations (1)

Awareness of Efforts (1)

Balanced Mood (1)

Banning Users (1)

Barriers to Getting License (1)

Battle in My Head (1)

Beating Oneself Up (1)

Becoming a Target (1)

Before and After (1)

Behavior Towards Others (1)

Behavioral Response (1)

Being High (1)

Being Labeled as 'Smart' (1)

Being Noticed by Others (1)

Being Patient (1)

Being an Old Soul (1)

Belief Change (1)

Belief Correction (1)

Belief in Abilities (1)

Belief in Best Outcome (1)

Belief in God's Goodness (1)

Belief in Individual (1)

Belief in Oneself (1)

Belief in the Outcome (1)

Belief of Undeserving (1)

Betting on Oneself (1)

Bias Management (1)

Bird Videos (1)

Blessing and Curse (1)

Blindness during Sickness (1)

Blocking Users (1)

Blood Flow Redistribution (1)

Blood Sugar Imbalance (1)

Blood pH Change (1)

Blurred Days and Missing Memories (1)

Blurred World Perception (1)

Body Aches (1)

Body Wants to Live (1)

Bonding Moments (1)

Bone Graft (1)

Boost in Humanity View (1)

Born in the 80s (1)

Bowel Issues (1)

Boxing (1)

Breathlessness (1)

Brigading (1)

Brightened up the day (1)

Broad Social Circle (1)

Broca’s Area Inactivation (1)

Bronchoconstriction (1)

Browsing Behavior (1)

Browsing Fresh Content (1)

Brushing Off (1)

Bubble Baths (1)

Building Each Other Up (1)

Building Social Skills (1)

Building up of Special Occasions (1)

Bushfires (1)

CBD Brand Endorsement (1)

CBD Oil (1)

CBD Usage (1)

Cactus Collection (1)

Call for Police (1)

Call for Submissions (1)

Call the Police (1)

Calling 911 Recommended (1)

Camping (1)

Can Cause Skin Damage (1)

Candida Yeast Overgrowth (1)

Canned Responses (1)

Cantaloupe (1)

Capability (1)

Caption Preference (1)

Career Focus (1)

Caring Individuals (1)

Caring Partner (1)

Caring Personality (1)

Caring for Physically Disabled Parent (1)

Caring for Siblings (1)

Caring for a Disabled Family Member (1)

Carrot & Stick Approach (1)

Carrying Random Items (1)

Casual Conversation (1)

Casual Conversations (1)

Casual Return (1)

Casual Tone (1)

Catastrophizing (1)

Catch 22 Situations (1)

Caught in Personal Issues (1)

Cause of Acne (1)

Cause of Impulsivity (1)

Celebratory Comment (1)

Celebrity (1)

Certain Prediction (1)

Challenges in Explanation (1)

Challenges of Single Income (1)

Challenges of Starting (1)

Challenging Mother (1)

Change in Social Circle (1)

Change of Environment (1)

Change of Internal Language (1)

Change of Location (1)

Changed Reaction to Substances (1)

Changed Worldview (1)

Changes in Appearance (1)

Changing Room Atmosphere (1)

Changing Surroundings (1)

Chat Option (1)

Checking In (1)

Chemistry and Timing (1)

Child Negligence (1)

Child Protective Services (1)

Child as Emotional Sponge (1)

Childfree by Choice (1)

Childism (1)

Children Program (1)

Choice and Reaction (1)

Choice in Reaction (1)

Choice in Response (1)

Cholesterol Levels (1)

Choosing No Contact (1)

Choosing Perceptions (1)

Choosing to Stay in Hula Hoop (1)

Christmas Card Signing Tradition (1)

Chronic Conditions Development (1)

Chronic Shortness-of-Breath (1)

Chronicity (1)

City Division (1)

Civility (1)

Clarify Cultural Differences (1)

Class Load Management (1)

Classification (1)

Clay Paw Print (1)

Clerks (1)

Client Experience (1)

Closing Discussion (1)

Club (1)

Co-parenting Challenges (1)

Coasting on Past Knowledge (1)

Cognitive Dissonance (1)

Cognitive Intrusion (1)

Cognitive Spiral (1)

Cold Sensation (1)

Cold Water Exposure (1)

Cold Water Splash (1)

Cold Water on Face (1)

Collecting Evidence (1)

Collective Experience (1)

Color Coding for Mood (1)

Combat Not Sole Cause (1)

Combat Strategy (1)

Combat-centric Perception (1)

Comfort in Disclosure (1)

Comfort with Oneself (1)

Comfortable with a Stranger (1)

Comment Deletion (1)

Commiseration (1)

Commitment Issues (1)

Common Occurrence (1)

Commonality of Condition (1)

Communicating with Friends (1)

Community Standards (1)

Community Struggles (1)

Company Inattention (1)

Company Policy (1)

Company Shutdown (1)

Comparative Criminal Liability (1)

Comparing Trends (1)

Comparing to Others' Skills (1)

Compelled Completion (1)

Compensation by Overachieving (1)

Competitiveness (1)

Completion Despite Delays (1)

Completion of Chores (1)

Complexity of Being Human (1)

Complexity of Conditions (1)

Concurrence (1)

Conditioned Emotional Responsiveness (1)

Conditioned Preferences (1)

Confiding in Someone (1)

Conflict Between Inner Voices (1)

Conforming to Social Groups (1)

Connecting with Roommates (1)

Conscious Decision to Have Children (1)

Consequence-free Living (1)

Consequences for Homophobia (1)

Conserving Mental Energy (1)

Consider Drop Down Levels (1)

Considerate Hosting (1)

Consideration of Others' Feelings (1)

Consideration of Overdose (1)

Consideration of Trade Education (1)

Considering Leaving Abusive Environment (1)

Constant 'What Ifs' (1)

Constant Edginess (1)

Constant Inner Dialogue (1)

Constant Monitoring and Criticizing (1)

Constant Programming (1)

Constant Striving (1)

Constructive Use of Traits (1)

Consultation with Attorney (1)

Consumption of Chapstick (1)

Contacting Old Acquaintances (1)

Content Accumulation (1)

Content Deletion (1)

Content Perception (1)

Content Quality (1)

Contextualizing (1)

Continuation of Life Post-Attempt (1)

Continued Behavior Through Life (1)

Continuous Epiphanies (1)

Continuous Therapies (1)

Contract Non-renewal (1)

Contradictory Emotions (1)

Contrasting Habits (1)

Cool Showers (1)

Coping with Emotional Turbulence (1)

Coping with Social Change (1)

Coping with Urges (1)

Corporate Mismanagement (1)

Cortisol Levels (1)

Cosmetic Surgery Consideration (1)

Cost of Chronic Agitation (1)

Counter Intuitive (1)

Counting (1)

Coverage of Material (1)

Covert Emotional Incest (1)

Coziness (1)

Crab Legs (1)

Craft Projects (1)

Creating Never Ending Lists (1)

Creating a Menu Item (1)

Creation of Illusions (1)

Creation of Megathread (1)

Creation of a Guide (1)

Cringeworthy Experiences (1)

Crippling Effect (1)

Crisis Response (1)

Criteria for Contacting Emergency Services (1)

Critical Approach (1)

Critical Evaluation of Putin's Speech (1)

Critical Thinking and Judgement (1)

Crossing the Street (1)

Crowds (1)

Cruelty of Endings (1)

Cruelty of Virus (1)

Crunchy Granola Liberal Area (1)

Crush (1)

Cultural Adjustment (1)

Curability of Vaginismus (1)

Curating Followings (1)

Cure for Brain Disorders (1)

Current State (1)

Current Year Highlight (1)

Custodianship of Inner Child (1)

Custom Response (1)

Customizable Items (1)

Cut Off Contact (1)

Cuteness-Based Breeding (1)

Cutting Contact (1)

Cutting People Off (1)

Cycle of Emotions (1)

Cynical Outlook (1)

DARE Strategy (1)

Daily Chores Commitment (1)

Daily Occurrence (1)

Daydreaming of an Alternative Life (1)

Deactivate Account (1)

Dealing with Difficult Conditions (1)

Debilitating (1)

Decades-long Use (1)

Decision to Log Off (1)

Decreased Activity (1)

Decreased Social Energy (1)

Dedication (1)

Default Mode Network (DMN) (1)

Defendant Strategy (1)

Defending Oneself (1)

Defensive Response (1)

Definition Clarity (1)

Definition Seeking (1)

Deflection Mechanism (1)

Delay in Opening Messenger (1)

Delayed Discovery (1)

Delayed Reaction (1)

Delayed Spell Check (1)

Delegation (1)

Deleting Posts (1)

Demanding Transparency (1)

Demeaning Language (1)

Demotivation (1)

Dependence on Technology (1)

Depiction in Comics (1)

Deserved Outcome (1)

Deserved Place (1)

Deserving Life (1)

Designated Driving (1)

Desire for Additional Mother Figures (1)

Desire for Better Lives for Children (1)

Desire for Better Options (1)

Desire for Care and Attention (1)

Desire for Closeness (1)

Desire for Easy Solution (1)

Desire for Energy Redistribution (1)

Desire for Genuine Care (1)

Desire for Good People Around (1)

Desire for Harmony (1)

Desire for Legitimization (1)

Desire for Ownership (1)

Desire for Parenthood (1)

Desire for Past Attractiveness (1)

Desire for Public Events (1)

Desire for Renewal (1)

Desire for Resolution (1)

Desire for Security (1)

Desire for Simple Solutions (1)

Desire for Solutions (1)

Desire for Storm (1)

Desire for Time and Freedom (1)

Desire for Trade (1)

Desire for WFH (1)

Desire for Younger Sibling (1)

Desire for a Simpler Life (1)

Desire to Cancel (1)

Desire to Drop Out (1)

Desire to Emulate (1)

Desire to Exit (1)

Desire to Experience 'Normal' (1)

Desire to Get Away (1)

Desire to Perform Well (1)

Desire to Possess Skills (1)

Desire to Purchase (1)

Desire to Relax (1)

Desire to Return to Berlin (1)

Desire to Vent (1)

Desired Resource (1)

Despondency (1)

Detached Conversations (1)

Detox from Multiple Platforms (1)

Detrimental Self-Defense (1)

Devaluation of Aggressor (1)

Developing Bond (1)

Development of Conditions (1)

Deviation from Norm (1)

Diagnosed in adulthood (1)

Diagnostic Dissonance (1)

Did not live with dinosaurs (1)

Difference in Approach (1)

Different Home Environments (1)

Different Parameters (1)

Different Pregnancy Experience (1)

Different Shits (1)

Differing Outcomes (1)

Difficult Mornings (1)

Difficult Patients (1)

Difficulties in Analyzing (1)

Difficulty Getting Out of Bed (1)

Digestive System Effects (1)

Digital Tools (1)

Diminishing Effect Over Time (1)

Diminishing Others' Opinions (1)

Diminishing Remarks (1)

Direct Interaction (1)

Direct Message Request (1)

Direct Messages (1)

Direction to Appropriate Resources (1)

Disagreement with Parent (1)

Disbelief in Popularity (1)

Discarding Long Comments (1)

Disconnect from Superficial Platforms (1)

Disconnection from Friends (1)

Discontentment with Existence (1)

Discord Between Self and Others' View (1)

Discount Offer (1)

Discrediting (1)

Discrepancy Between Appearance and Reality (1)

Discrepancy in Public and Private Behavior (1)

Discrepancy in Technological Advancement (1)

Discrepancy in Work Policies (1)

Discrepancy with Real World (1)

Discussing Reasons (1)

Disdain for Authority (1)

Disgust and Violation (1)

Disheartenment (1)

Disinterest in Video Games (1)

Dislike of Mental State (1)

Dismissal of Feelings (1)

Dismissal of Materialism (1)

Dismissal of Moodiness (1)

Dismissal of Phrases (1)

Dismissed Emotions (1)

Disorganization of Personal Items (1)

Disparagement (1)

Disputing Patient's Experience (1)

Disregard for Education (1)

Disregard for Others' Feelings (1)

Disrupted Thinking (1)

Distinction Between Fiction and Reality (1)

Distinguishing Thoughts and Feelings (1)

Distrust in Bodily Health (1)

Diverse Factors (1)

Diverse Formats (1)

Diverse Reactions (1)

Diversification of Efforts (1)

Diversity (1)

Diversity in College (1)

Doctorate (1)

Dog Walking (1)

Doing Good Deeds (1)

Doing What's Right (1)

Dominating Conversations (1)

Don't Give Up (1)

Double Checking (1)

Double Life Feeling (1)

Double-Edged Sword (1)

Download Data (1)

Downloading Activity (1)

Drink Options (1)

Drive to Care for Others (1)

Dropping Performance (1)

Dry Mouth (1)

Dry Skin (1)

Dumbing down a Song (1)

Duration of Grief (1)

Dwelling on Past Events (1)

Dysfunctionality (1)

ERP Therapies (1)

Early Awakening (1)

Early Internet Barriers (1)

Early Wakening (1)

Earplugs (1)

Ears and Balance (1)

Ease of Publishing (1)

Easier to Help Others (1)

Easy to Digest Food (1)

Eating Disorder (1)

Eccentric Behaviors (1)

Economic Barrier to Access (1)

Economic Barriers (1)

Economic and Emotional Costs (1)

Eczema (1)

Eczema Flare-up (1)

Educating Younger Family Members (1)

Educational Awareness (1)

Educational Videos (1)

Effect of Endorphin Withdrawal (1)

Effect of Online Schooling (1)

Effective Handling (1)

Effects on Social Life (1)

Effort to Preserve Harmony (1)

Effort to Socialize (1)

Ego Dynamics (1)

Electronic Drum Kits (1)

Email Checking (1)

Emails (1)

Embracing Diversity (1)

Embracing Emotions (1)

Embracing Features (1)

Embracing Gray Hair (1)

Embracing Personal Agency (1)

Embracing Variability (1)

Embracing the Moment (1)

Embracing the Normal (1)

Emergency Assistance (1)

Emergency Housing Services (1)

Emergency Resources (1)

Emotional Adjustment (1)

Emotional Dissonance (1)

Emotional Hangover (1)

Emotional Hurt (1)

Emotional Instability (1)

Emotional Intensity (1)

Emotional Journey (1)

Emotional Labor (1)

Emotional Problems (Sister) (1)

Emotional Rebirth (1)

Emotional Spiral (1)

Emotional Stability (1)

Emotional Turbulence (1)

Emotional Unhappiness (1)

Emotional Uplift (1)

Emotional Void (1)

Emotional Volatility (1)

Emotional Weariness (1)

Emotive punctuation usage (1)

Empathetic Expression (1)

Empathetic Reach-Out (1)

Empathetic Statement (1)

Emphasis on Longevity (1)

Emphasizing Importance (1)

Employment Dissatisfaction (1)

Enabling (1)

Encountering Abusiveness (1)

Encouraged Topics (1)

Encouraging Attitude (1)

Encouraging Comments (1)

Encouraging Others (1)

Encouraging Outreach (1)

End of Friendship (1)

Endangering Behavior (1)

Endless Source of Problems and Solutions (1)

Enduring Effects (1)

Engaging in Conversation (1)

Engaging in Fights (1)

Engaging in Harmful Activities (1)

Engrossment (1)

Enhanced Mental Clarity (1)

Enjoying Distance (1)

Enjoying the Act of Driving (1)

Enjoying the Present (1)

Enlistment in the Army (1)

Entertaining Videos (1)

Entertainment (1)

Enthusiastic Return (1)

Entrepreneurship (1)

Environmental Change (1)

Environmental Influence (1)

Environmental Stewardship (1)

Equality (1)

Equality Statement (1)

Equipment Readiness (1)

Ergonomic Improvements (1)

Ethics and Competitors (1)

Etiquette Breach (1)

Evaluating Options (1)

Evaluating Responses (1)

Everyman (1)

Evidence-based Thinking (1)

Exaggerated Response (1)

Exaggerated Sacrifice for Emphasis (1)

Exaggeration for Effect (1)

Exceptional Quality (1)

Exceptions during Crisis (1)

Exclusion of Infants Based on Physical Ability (1)

Existence of Crappy People (1)

Existence of Solutions (1)

Existential Nihilism (1)

Existential Thoughts (1)

Expectation of a Reason (1)

Experiences over Tangible Gifts (1)

Experiencing Cruelty (1)

Experiencing Loss (1)

Experiential Knowledge Requirement (1)

Explanation of Disadvantages (1)

Explanatory Response (1)

Exploitation Feeling (1)

Exploring Accommodations (1)

Exploring Further Medical Opinions (1)

Exploring New Places (1)

Expressing Need for Conversation (1)

Expressing Opinions (1)

Expression of Belief (1)

Expression of Catch 22 (1)

Expression of Pity (1)

Extended Memory to Other Loved Ones (1)

External Absolution (1)

External Failure (1)

External Reference (1)

External Resource (1)

External Situational Factors (1)

Extra Sunlight (1)

Eye Contact (1)

Eye Twitching (1)

FMLA (1)

Face Masks (1)

Facilitating Access to Help (1)

Facing Reality (1)

Fading SAD (1)

Faith in Body's Abilities (1)

Faith in the Future (1)

False Ideas about Others (1)

Familiar Landmarks (1)

Family Achievements (1)

Family News (1)

Family Punctuality (1)

Family Seclusion (1)

Family and Home (1)

Family as Random Chance (1)

Fantasy vs Reality (1)

Fast Years (1)

Fastereft.com (1)

Fasting Techniques (1)

Fatalistic Thinking (1)

Father's Day Without Father (1)

Favorite Place (1)

Favoritism (1)

Fed Up Collection (1)

Feedback Loop (1)

Feeding Wildlife (1)

Feel Good (1)

Feeling Abnormal (1)

Feeling Alienated (1)

Feeling Amazing (1)

Feeling Attacked (1)

Feeling Betrayed by Friends (1)

Feeling Better (1)

Feeling Better After Decision (1)

Feeling Comfortable (1)

Feeling Deluded (1)

Feeling Desperate (1)

Feeling Determined (1)

Feeling Dramatic (1)

Feeling Excluded (1)

Feeling Faint (1)

Feeling Free (1)

Feeling Imposed Upon (1)

Feeling Inauthentic (1)

Feeling Invalid (1)

Feeling Lazy (1)

Feeling Let Down (1)

Feeling Liberated (1)

Feeling Like a Failure (1)

Feeling Misled (1)

Feeling Mortified (1)

Feeling Not Okay (1)

Feeling Overly Sensitive (1)

Feeling Programmed (1)

Feeling Rotten at Core (1)

Feeling Sad and Dramatic (1)

Feeling Secure (1)

Feeling Sexy (1)

Feeling Shrunk or Inflated (1)

Feeling Sophisticated (1)

Feeling Spoiled (1)

Feeling Stronger (1)

Feeling Surrounded by Irrationality (1)

Feeling Targeted (1)

Feeling Unanchored (1)

Feeling Unjustified (1)

Feeling Vulnerable (1)

Feeling after Eating (1)

Feeling at Ease (1)

Feeling like a Failure (1)

Feeling like a Loser (1)

Feeling of Being Tolerated (1)

Feeling of Camaraderie (1)

Feeling of Chore (1)

Feeling of Deadness (1)

Feeling of Everything Being Too Much (1)

Feeling of Heaviness (1)

Feeling of Impossibility (1)

Feeling of Inability (1)

Feeling of Incompleteness (1)

Feeling of Lack (1)

Feeling of Life Being Over (1)

Feeling of Life Passing By (1)

Feeling of Meaninglessness (1)

Feeling of Not Fitting In (1)

Feeling of Observing (1)

Feeling of Ordinariness (1)

Feeling of Paradise (1)

Feeling of Permanence (1)

Feeling of Readiness to Give Up (1)

Feeling of Ruin (1)

Feeling of Similarity (1)

Feeling of Suckiness (1)

Feeling of Surreality (1)

Feeling of Wasted Time (1)

Feeling of Wasting Others' Time (1)

Feeling of Worthlessness (1)

Feeling the Same Way (1)

Feelings Reemergence (1)

Feelings of Agedness (1)

Feelings of Faking (1)

Feelings of Incompetence (1)

Feelings of Inefficiency (1)

Feelings of Letting Others Down (1)

Feelings of Uselessness (1)

Felony Charges (1)

Fetal Movement (1)

Fight Against Suicidal Urges (1)

Fight Types (1)

Figuring out Treatment Plan (1)

Filtering Comments (1)

Final Goodbyes (1)

Finality of Aging (1)

Finality of Death (1)

Finality of Situation (1)

Financial Aid (1)

Financial Assistance (1)

Finding Common Ground (1)

Finding Direction (1)

Finding Effective Methods (1)

Finding Goodness in Everyone (1)

Finding Meaning in Simple Pleasures (1)

Finding Middle Ground (1)

Finding Personal Reason (1)

Finding Reason to Live (1)

Finding Someone Compatible (1)

Finding a Way (1)

First Attempt (1)

First-time Participation (1)

Fitness Tracker Accuracy (1)

Flat Earth Belief (1)

Flight Class Preference (1)

Flourishing Life (1)

Fluctuating Social Behavior (1)

Flying Alone (1)

Focus on Humanity (1)

Focus on Immediate (1)

Focus on Negatives (1)

Focusing on Important Aspects (1)

Focusing on the Present (1)

Following Child Home (1)

Following Spiritual Teacher (1)

Following Trends (1)

Fond Memories (1)

Fondness for Cactus (1)

Food Sensitivities (1)

Football (1)

Forced Eating (1)

Forced Normality (1)

Forcing Yourself (1)

Formation of Activision (1)

Fortune Telling (1)

Freedom from Daily Commute (1)

Frequent Activity (1)

Frequent Behavior (1)

Frequent Expression (1)

Frequent Urination (1)

Friend Incarceration (1)

Frustration with Finding Purpose (1)

Fulfilling Preferences (1)

Fulfillment through Volunteering (1)

Fun Uncle/Aunt (1)

Fun in Decorating (1)

Function Impairment (1)

Functionality Maintenance (1)

Future Focus (1)

Future Opportunities (1)

Gaining Insight and Wisdom (1)

Gallbladder Problems (1)

Generalization (1)

Generational Behavior (1)

Generational Difference (1)

Generational Transition (1)

Genius Status (1)

Gentle Approach (1)

Geographic Location (1)

Geographical Change (1)

Getting Out of Bed (1)

Getting Started (1)

Getting out the Door (1)

Getting to Know Each Other (1)

Gift-Giving (1)

Gifting (1)

Giving Back (1)

Glasses Fogging (1)

Glitch (1)

Global Need for Societal Focus (1)

Going the Extra Mile (1)

Golden Handcuffs (1)

Good Thoughts (1)

Goodwill (1)

Google for Information (1)

Grain of Salt (1)

Grandchildren (1)

Greyhounds (1)

Grim Outlook on Human Conditions (1)

Grouchy Owner (1)

Groundhog Day (1)

Group Assignments (1)

Grouping Codes into Categories (1)

Guidance on Re-posting (1)

Guild Formation (1)

HALT Method (1)

HR Complaint (1)

HR Involvement (1)

Hair Care (1)

Hair Color Change (1)

Hair Dye (1)

Haircut (1)

Hand Activities (1)

Handling Raises (1)

Handwriting Resemblance (1)

Hang in (1)

Hang in there (1)

Hangover-like Feeling (1)

Hard-working (1)

Harmful Habits (1)

Harsh Awakening (1)

Harshness Towards Children (1)

Hating Chosen Industry (1)

Healthy Relationships (1)

Hearing Aid and Cochlear Implant Complexity (1)

Heart Rate Management (1)

Heartburn (1)

Helpful Technique (1)

Hemorrhoid (1)

Hiding Cult Background (1)

Hiding Personal Issues (1)

Hiding True Feelings (1)

High Cortisol Effects (1)

High Energy Periods (1)

High Functioning Autism (1)

High Ideals (1)

High Prolactin Levels (1)

High Score Mentality (1)

High Volume of Posts (1)

Highlight Reel Syndrome (1)

Highlighting Irony in Social Reactions (1)

Historical Figures (1)

Historical Influence (1)

Holding Businesses Accountable (1)

Holding False Beliefs (1)

Holding On (1)

Holiday Traditions (1)

Holiday Trip (1)

Holistic Approach to Treatment (1)

Hollywood Illusions (1)

Home as a Symbol (1)

Homework (1)

Homework Overload (1)

Hostility (1)

Hot Bath (1)

Hotline Failure (1)

Hotlines (1)

Household Infestations (1)

Housework on Saturday (1)

How to Do Nothing (1)

Howl's Moving Castle (1)

Hugs (1)

Human Validity (1)

Humanitarianism (1)

Humble Response (1)

Hunger Response (1)

Hurtful Comments (1)

Hurting Others (1)

Hyperactivity (1)

Hypercritical Home (1)

Hypertension (1)

Hysteria (1)

Ideal Morning (1)

Ideal of Above Average (1)

Identifying Key Themes (1)

Identifying the True Inner Voice (1)

Identifying with Someone (1)

Ignored Feelings (1)

Ignoring Negative Voices (1)

Illegal Activity (1)

Imagining Extreme Scenarios (1)

Immature Response (1)

Immediate Action Required (1)

Immediate Gratification (1)

Immediate Police Response (1)

Immediate Removal (1)

Immediate Response (1)

Immersive Fun Approach (1)

Immigration to the US (1)

Impersonal Response (1)

Implausibility of Immediate Adjustment (1)

Implementation and Follow-through (1)

Implementation of Tips (1)

Importance of Attitude (1)

Importance of Being There (1)

Importance of Digging for Answers (1)

Importance of Diligence (1)

Importance of Feeling (1)

Importance of Garbage Men (1)

Importance of Onboarding (1)

Importance of Origin (1)

Importance of Present Tasks (1)

Importance of Risk Assessment (1)

Importance of Talking (1)

Importance of Togetherness (1)

Importance of Upvotes (1)

Importance of the Moment (1)

Imposed Idealization (1)

Impression Management (1)

Improved Management (1)

Improving Mental Clarity (1)

Improving One's Situation (1)

Improvised Solutions to Infestations (1)

Impulsive Actions (1)

Inability to Accept Loss (1)

Inability to Accomplish Tasks (1)

Inability to Engage (1)

Inability to Function (1)

Inability to Let Go (1)

Inability to Offer Direct Help (1)

Inability to Save All (1)

Inability to Work (1)

Inaccurate Portrayal (1)

Inaccurate Use of 'Sue' (1)

Inadequate Advice (1)

Inadequate Compensation (1)

Inappropriate Information Disclosure (1)

Inappropriate Roles (1)

Inappropriate for Long-term (1)

Inappropriateness of Feelings (1)

Inattentive ADD (1)

Inauthenticity (1)

Incoherence (1)

Incompetent Manager (1)

Incomprehension (1)

Inconsistent Feelings (1)

Inconsistent Habits (1)

Inconsistent Reactions to Suicidality (1)

Inconvenient Objects (1)

Inconvenient Store Hours (1)

Incorporating DBT Skills (1)

Incorporating Movement (1)

Incorrect Information (1)

Incorrect Usage of Terms (1)

Incorrect Use (1)

Increase in Going Out (1)

Increased Adrenaline (1)

Increased Consumption During Intense Thinking (1)

Increased Focus on Problems (1)

Increased Hunger (1)

Increased Normalcy and Energy (1)

Increased Obsessions (1)

Increased Severity (1)

Incredulity (1)

Indication of Seeing Something Unusual (1)

Indicator of Mental State (1)

Indifference towards Responsibilities (1)

Indulgence (1)

Indulging in Entertainment (1)

Inexcusable Behavior (1)

Influence of Fictional Characters (1)

Influence of Philosophical Teachings (1)

Influence on Choices (1)

Influence on Science Fiction (1)

Influential Books (1)

Informal Endearment (1)

Informal Tone (1)

Information Retrieval (1)

Informing (1)

Informing Coworkers (1)

Ingrained Characteristics (1)

Inherent Goodness (1)

Inherent Meaning of Life (1)

Initial Mail (1)

Inquiring About First Language (1)

Instant Switch (1)

Instilling Money Sense (1)

Instructional Videos (1)

Insufficient Protective Equipment (1)

Insufficient Wages (1)

Intellectual Development (1)

Intelligence Goes Beyond Grades (1)

Intelligent Response (1)

Intense Emotional Experience (1)

Intense Mental Gymnastics (1)

Intense Sleep (1)

Intensifier (1)

Intensity Throughout Cycle (1)

Intent to Clean Own Space (1)

Intent to Share (1)

Intent to Use (1)

Intent to try suggestion (1)

Intention to Acquire Resources (1)

Intention to Return (1)

Intention to Utilize More (1)

Intentional Activity Replacement (1)

Intentional Conception (1)

Interest in Holiday (1)

Intermittent Clarity (1)

Intermittent Fasting (1)

Internal Age Mismatch (1)

Internal Dialogue (1)

Internal Project Transfer (1)

Internal Resources (1)

Internal Voices (1)

Internet Browsing (1)

Interstate Travel (1)

Intimate Conversations (1)

Intolerance for Mediocrity (1)

Intolerance of Pretense (1)

Intricately Intertwined (1)

Intrinsic Qualities (1)

Intuition (1)

Invisible Disability (1)

Involuntary Condition (1)

Involvement in Activities (1)

Involvement of Child and Family Services (1)

Irrational Dissonance (1)

Irregular Bathing (1)

Irregular Schedule (1)

Irrelevant Questions (1)

Irreversible Damage (1)

Isolating Behavior (1)

Itching (1)

Job Misfit (1)

Job Perception (1)

Joint Effort (1)

Joseph Merrick's Disposition (1)

Justification for Indoor Time (1)

Justifying Feelings (1)

Karma (1)

Keeping Promises (1)

Keeping Things to Yourself (1)

Kick when Down (1)

Kickboxing (1)

Kitchen Sofa (1)

Knowledge Without Benefit (1)

Labeling as Hypocrite (1)

Labor Camp Experience (1)

Lack of Attachment (1)

Lack of Awareness in Society (1)

Lack of Belief from Others (1)

Lack of Common Sense (1)

Lack of Compatibility (1)

Lack of Compensation (1)

Lack of Consistency (1)

Lack of Customization (1)

Lack of Direction (1)

Lack of Discussion (1)

Lack of Evidence (1)

Lack of Experienced Guidance (1)

Lack of Faith in the Future (1)

Lack of Fault (1)

Lack of Financial Security (1)

Lack of Follow-up (1)

Lack of Fullness (1)

Lack of Functional Skills (1)

Lack of Funds (1)

Lack of Future Outlook (1)

Lack of Gratification (1)

Lack of Household Items (1)

Lack of Human Contact (1)

Lack of Information (1)

Lack of Insurance (1)

Lack of Interest in Goals (1)

Lack of Leisure (1)

Lack of Life Fulfillment (1)

Lack of Life Skills Education (1)

Lack of Life in Eyes (1)

Lack of Love (1)

Lack of Mental Stamina (1)

Lack of Natural Light (1)

Lack of Parental Introspection (1)

Lack of Participants (1)

Lack of Personal Funds (1)

Lack of Personal Resources (1)

Lack of Pleasure in Eating (1)

Lack of Pocket Space (1)

Lack of Positive Care (1)

Lack of Practical Opportunities (1)

Lack of Prior Studying Habit (1)

Lack of Proof (1)

Lack of Protection and Care (1)

Lack of Reaction to Environmental Stimuli (1)

Lack of Skills (1)

Lack of Snow (1)

Lack of Tissues (1)

Lack of Tolerance (1)

Language Miscommunication (1)

Language Proficiency (1)

Laser Surgery (1)

Last Conversation (1)

Late Arrival (1)

Late Rising (1)

Late-Night Homework (1)

Laundry Location (1)

Laundry Management (1)

Leading by Example (1)

Lean Into the Feeling (1)

Learned Behavior from Parent (1)

Learning Disabilities (1)

Learning Resources (1)

Learning to Study and Apply Oneself (1)

Legal Issues (1)

Legal Protection (1)

Legal Recourse (1)

Leisurely Return (1)

Leisurely Start to the Day (1)

Lending an Ear (1)

Letting Go of the Old Thought (1)

Leveraging Absence (1)

Leveraging Personal Circumstances (1)

Life Perception (1)

Life Pessimism (1)

Life as a Game (1)

Life's Direction (1)

Lifelong Effects (1)

Light Management (1)

Light at the End of the Tunnel (1)

Light-Heartedness (1)

Light-hearted Reaction (1)

Light-heartedness (1)

Lighting Adjustment for SAD (1)

Liking Others' Art (1)

Limitations in Providing Opportunities (1)

Limitations on Lifting (1)

Limited Ability to Help (1)

Limited Access to Shops (1)

Limited Attendance at Funeral (1)

Limited Attention (1)

Limited Benefit (1)

Limited Employment Options (1)

Limited Following (1)

Limited Online Presence (1)

Limited Outdoor Activities (1)

Limited Reddit Usage (1)

Limited Scope (1)

Limited Selection (1)

Limited Social Circle (1)

Limited Social Groups (1)

Limited Tolerance (1)

Limiting Driving Behavior (1)

Limiting Topics of Discussion (1)

Lingerie (1)

Link Issue (1)

List Making (1)

Lists (1)

Littering (1)

Living Comfortably (1)

Living Nightmare (1)

Living Situation (1)

Living Vicariously (1)

Living a Good Life (1)

Living as a Statement (1)

Lizard Brain Response (1)

Location Query (1)

Locking Post (1)

Locking Threads (1)

Locking of Discussion (1)

Long Absence (1)

Long Days (1)

Long Showers (1)

Long Wait (1)

Long Working Hours (1)

Long-Term Goals (1)

Long-Term Singlehood (1)

Long-Term Unemployment (1)

Long-Term Unhappiness (1)

Long-term Absence of Peace (1)

Long-term Result (1)

Long-term Weed Usage (1)

Longevity (1)

Lose Lose Situation (1)

Losing Sense of Authentic Self (1)

Losing Something Important (1)

Loss of Carefreeness (1)

Loss of Childhood (1)

Loss of Direction (1)

Loss of Faith in People (1)

Loss of Functionality (1)

Loss of Meaning (1)

Loss of Passion (1)

Loss of Pleasure (1)

Loss of Smell (1)

Loss of Specific Recollections (1)

Loss of Speech (1)

Loss of Years (1)

Lost Opportunities (1)

Lost Time (1)

Low Blood Sugar (1)

Low Maintenance (1)

Low Quality Content (1)

Low Resource Consumption (1)

Low Risk Perception (1)

Loyalty to Company (1)

Lymph Node Swelling (1)

MMR Vaccination (1)

MTHFR Gene (1)

Machining Experience (1)

Made-up Posts (1)

Madness and Genius (1)

Magnesium (1)

Maintaining Options (1)

Maintaining Professional and Social Life (1)

Making Bets (1)

Making Them Proud (1)

Making the Best of Now (1)

Maladaptive Daydreaming (1)

Management of Adrenaline Rush (1)

Managing Flare Ups (1)

Managing Seasonal Affective Disorder (SAD) (1)

Mandatory Overtime (1)

Manual Coding (1)

Mars Truthers (1)

Matchmaking (1)

Materialistic Bragging (1)

Materials Collection (1)

Me (1)

Meal Planning (1)

Media-induced Intrusions (1)

Medical Assistance (1)

Meeting Creators (1)

Meeting with Manager (1)

Meltdown (1)

Membership (1)

Memorable Event (1)

Memorable Events (1)

Memorializing the Deceased (1)

Mental Activities (1)

Mental Agitation (1)

Mental Energy Boost (1)

Mental Shift Strategies (1)

Mentality Shift (1)

Mentally Preparing Self-Explanation (1)

Method of Consumption (1)

Metric System vs Imperial System (1)

Micro Apartments (1)

Micropenis (1)

Mid-Spring Peak (1)

Mild Response (1)

Mind Wandering (1)

Mind as Self-preserving (1)

Minimal Content (1)

Minimal Standards for Keeping Items (1)

Minimizing Presence (1)

Miscommunication on Efforts (1)

Misconduct (1)

Miserable Childhood (1)

Miserable Existence (1)

Misjudging Social Cues (1)

Misjudgment of Outcomes (1)

Misleading Appearances (1)

Mismatched Engagement (1)

Misogynistic Language in Dating (1)

Misperception of Threat (1)

Misrepresentation of Beliefs (1)

Misrepresentation of Deaf Experience (1)

Misrepresentation of Feelings (1)

Misrepresenting Patient's Health Condition (1)

Missed Opportunity (1)

Missing Content (1)

Missing Context (1)

Missing the Past (1)

Misunderstanding about licensing consequences (1)

Misunderstanding of Term (1)

Misunderstood Laziness (1)

Mixed Bag of PsyT Bloggers (1)

Mixed Messages (1)

Mobile Apps (1)

Mobile Browsing (1)

Mobile Compatibility (1)

Moderation Decision (1)

Moderator Announcement (1)

Modifying Tasks (1)

Moisturizer (1)

Monologuing (1)

Mood-Dependent Behavior (1)

Mother's Reactions (1)

Multiple Contributing Factors (1)

Multiple Thread Activity (1)

Mumbling in Sleep (1)

Myself & Irene (1)

NATO Escalation (1)

Naive Comment (1)

Name Coincidence (1)

Narrative Reprocessing (1)

Nasal Congestion (1)

National Domestic Violence Crisis Line (1)

Nationality (1)

Natural Aging Process (1)

Natural Instinct (1)

Need for Centralized Discussion (1)

Need for Comprehensive Solution (1)

Need for Distance (1)

Need for More Volunteers (1)

Need for Professional or Medical Guidance (1)

Need for Space to Grieve (1)

Need for Standardized Data Collection (1)

Need to Share (1)

Negative Assumptions (1)

Negative Connotation (1)

Negative Correlation (1)

Negative Incentives (1)

Negative Interactions (1)

Negative Life Outcomes (1)

Negative Maternal Behavior (1)

Negative Online Experience (1)

Negative Outlook on the Future (1)

Negative Parental Labels (1)

Negative Perception of Life (1)

Negative Reaction (1)

Negative Reaction to Disclosure (1)

Negative Remarks (1)

Negative Social Encounter (1)

Negative Social Experience (1)

Negative assumptions based on personal interests (1)

Negative impact on job and relationships (1)

Neglected Household Chores (1)

Neglecting Kitchen (1)

Negligence and Immaturity (1)

Negotiated Sentence (1)

Negotiating Better Terms (1)

Negotiation Over Prices (1)

Nervous System Dysregulation (1)

Nervous System Response (1)

Nest Building (1)

Neural Rerouting (1)

Neurobiological Changes (1)

Neurological Repair (1)

Neutral Zone (1)

New Reality (1)

New and Unknown Situation (1)

Niece's Nickname (1)

Nieces and Nephews (1)

Nietzsche as a Philosopher (1)

Night Guard (1)

Night Line (1)

Night Mood (1)

Nightmares of Suicide (1)

Nihilistic Life Philosophy (1)

No Cavities Found (1)

No Commuting (1)

No Contact Deliveries (1)

No Context Provided (1)

No Details Available (1)

No Issues (1)

No Longer Needs to Track (1)

No Need for Cure (1)

No Need to Worry (1)

No Negative Consequences (1)

No Plan (1)

No Quick Fixes (1)

No Reason to Cry (1)

No Set Standards (1)

No Shame in Feelings (1)

No Size Limitation in Skateboarding (1)

No Small Talk (1)

No Vitriolic Comments (1)

Nobility (1)

Non-Desire for Babies (1)

Non-Desire for Pregnancy (1)

Non-Friend Counsel (1)

Non-Participation in Christmas (1)

Non-Traditional Heroes (1)

Non-Traditional Remedies (1)

Non-Verbal Interaction (1)

Non-confrontational Interaction (1)

Non-constructive Conversation (1)

Non-essential Items Attraction (1)

Non-participation (1)

Non-political (1)

Nonchalance towards Gray Hair (1)

Normality of Feelings (1)

Normalize Feelings (1)

Normalizing Emotions (1)

Normalizing Feelings (1)

Not Angry (1)

Not Broken (1)

Not Bursting the Bubble (1)

Not Caring About Others' Opinions (1)

Not Compulsory (1)

Not Interested in Provided Resource (1)

Not Laziness (1)

Not Lazy (1)

Not Needing to Talk (1)

Not Overburdening Oneself (1)

Not Overreacting (1)

Not Shy (1)

Not Taking Life Too Seriously (1)

Not Taking it Personally (1)

Not an Anime Fan (1)

Notable Birthmark (1)

Nothing is Impossible (1)

Novelty of Thought (1)

Number Reference (1)

Number of Sessions (1)

Nurses as Heroes (1)

OSDD (1)

Object Misidentification (1)

Objection to Capitalist Life (1)

Objective vs. Subjective Reality (1)

Objectives (1)

Observing Emotional Change (1)

Observing Others' Ease (1)

Obsessing Over Messages (1)

Obtaining a Diploma (1)

Occasional Recurrence (1)

Off-topic Discussions (1)

Offer of Reconsideration (1)

Offering Gifts (1)

Offering Upvote (1)

Office Space (1)

One-Night Experience (1)

Online Accessibility (1)

Online Classes (1)

Online Content (1)

Online Health Resources (1)

Online Healthcare Resources (1)

Online Information (1)

Online Ordering (1)

Online Purchase (1)

Online Research (1)

Online Services (1)

Online Survey (1)

Onset in Puberty (1)

Open Invitation for Help (1)

Openness to Trying Techniques (1)

Opposition to Obedience (1)

Opt-out Process (1)

Optimizing (1)

Order Placement (1)

Ordering Steps (1)

Ordering System (1)

Ordinary Behavior (1)

Organ Damage (1)

Other Options (1)

Others' Reaction (1)

Outcast (1)

Outreach Invitation (1)

Outsourcing (1)

Over-Inflated Role (1)

Over-Reaction (1)

Overactive Response (1)

Overanalyzing Interactions (1)

Overcoming Floundering (1)

Overcoming Limiting Beliefs (1)

Overcoming Negative Moods (1)

Overcompensation and Delusion (1)

Overconfidence (1)

Overexposure to News (1)

Overgeneralization (1)

Overload of Stimulus (1)

Overloaded (1)

Oversimplification Harm (1)

Overstretching (1)

Overuse of Clapping Emoji (1)

Overwork (1)

Overworked (1)

Overworking (1)

Oxygen Utilization (1)

PMDD (1)

Pacemaker Implementation (1)

Packed Meetings (1)

Parental Negligence (1)

Parental Response to Hunger (1)

Parents Recognizing and Changing (1)

Part-Based Release (1)

Participation Call (1)

Passing Time (1)

Passive Living (1)

Passivity (1)

Passivity vs. Activity (1)

Past Strategies (1)

Path of Least Resistance (1)

Patient Complaints (1)

Pattern of Self-sacrifice (1)

Peers' Wisdom (1)

Pep Talks (1)

Perceived Abnormality (1)

Perceived Absurdity (1)

Perceived Bottom (1)

Perceived Causality (1)

Perceived Child Inaccuracy (1)

Perceived Decline (1)

Perceived Exclusion (1)

Perceived Exoticism (1)

Perceived Ignorance (1)

Perceived Incompetence (1)

Perceived Inefficacy (1)

Perceived Irrationality (1)

Perceived Lack of Understanding from Others (1)

Perceived Life Deterioration (1)

Perceived Low Risk (1)

Perceived Negligence (1)

Perceived Normalcy (1)

Perceived Personal and World Dysfunction (1)

Perceived Psychic Abilities (1)

Perceived Ridiculousness (1)

Perceived Simplification of Problems (1)

Perceived Societal Decline (1)

Perceived Suddenness (1)

Perceived Threats (1)

Perceived Uselessness (1)

Perceived as Choice (1)

Perception Issues (1)

Perception as Pessimist (1)

Perception of Aging (1)

Perception of Art (1)

Perception of Compounded Negative Events (1)

Perception of Entitlement (1)

Perception of Life as Struggle (1)

Perception of Mundane Routine (1)

Perception of Portions (1)

Perception of Tasks as Laborious (1)

Perception of Threat (1)

Perception of Unchangeability (1)

Perceptual Distortion (1)

Performativity in Society (1)

Periodic Maintenance (1)

Permanence of Feeling (1)

Permanent Physical Damage (1)

Permission from OP (1)

Perpetrators' Intentions (1)

Perpetual Problems (1)

Perplexity (1)

Persistent Mindset (1)

Persistent Negative State (1)

Persistent Unsettling Feelings (1)

Persistent Wetness (1)

Personal Belief (1)

Personal Beliefs (1)

Personal Crisis (1)

Personal Outreach (1)

Personal Projects (1)

Personal Remedy (1)

Personal Sacrifice (1)

Personal Spotlight (1)

Personalized Meaning (1)

Personalized Weddings (1)

Personification of Mental State (1)

Perspective Shifting (1)

Persuasion (1)

Pervasive Melancholy (1)

Pet Tax (1)

Philosophical Influence (1)

Philosophy Implementation (1)

Phone Call Reliance (1)

Phone Number (1)

Physical Aging (1)

Physical Assistance (1)

Physical Change (1)

Physical Consequences (1)

Physical Defense Mechanisms (1)

Physical Distance (1)

Physical Distancing (1)

Physical Impairment (1)

Physical Reactions (1)

Physical Techniques (1)

Pious Front (1)

Place Name (1)

Plan to Review Content (1)

Platform Customization (1)

Playful Arguments (1)

Playful Insult (1)

Playful Teasing (1)

Playful Tone (1)

Pleasurable Situation (1)

Pleasure Activities (1)

Pleasure as Ephemeral (1)

Plenty of Options (1)

Plot Believability (1)

Plot Twist (1)

Plucking Technique (1)

Plush by STP (1)

Poignancy (1)

Pomodoro Technique (1)

Pooping Anxieties (1)

Poor Decision Making (1)

Poor Eating Habits (1)

Popular Phrases (1)

Popularity (1)

Porters (1)

Positive Content Filter (1)

Positive Farewell (1)

Positive Focus (1)

Positive Mental State (1)

Positive Perception of the Sub (1)

Positive Personal Judgment (1)

Positive Remembrance (1)

Positive Role Model (1)

Positive Self-image in Young Teens (1)

Positive Self-perception (1)

Post Deletion (1)

Post Length (1)

Post Removal (1)

Post Update (1)

Post-Crisis Reaction (1)

Post-Disaster Aid (1)

Posthumous Attention (1)

Posting Free Stuff (1)

Posting Requirements (1)

Potency of Smell (1)

Potential Post (1)

Power of Conversation (1)

Practicing Description (1)

Practicing Responses (1)

Pragmatism (1)

Praising Attitude (1)

Pre-digital Publishing Process (1)

Predatory Behavior (1)

Predictability (1)

Predictable Reaction (1)

Predictable Reactions (1)

Preferable to Unemployment (1)

Preference for Bearaby (1)

Preference for Fictional Names (1)

Preference for Local Products (1)

Preference for Muji Gel Pens (1)

Preference for Online Classes (1)

Preference for Own Terms (1)

Preference for Speaking (1)

Preference for Sunday Mornings (1)

Preferred Social Circle (1)

Premature Burdens (1)

Premature Graying (1)

Premature Solutions (1)

Preparation for Civil Unrest (1)

Preparation for Ordering (1)

Prepared Responses (1)

Preparing for Performance (1)

Presence of Goodness (1)

Preservation of Family Image (1)

Pretending to be okay (1)

Pretense as a Strategy (1)

Previous Attempts (1)

Price Range (1)

Print Media Constraints (1)

Priority on Personal Issues Over New Knowledge (1)

Prison (1)

Proactive Management (1)

Probation (1)

Product Seeking (1)

Professional Appearance (1)

Professional Assistance (1)

Professional Conduct (1)

Professional Gatekeeping (1)

Professional Opinion (1)

Professional Role (1)

Professor's Personality (1)

Professor's Preference (1)

Profit-Driven (1)

Prohibited Topics (1)

Prohibiting Harmful Comments (1)

Project Deadlines (1)

Promise to Return (1)

Prompt Completion (1)

Prompt Repairs (1)

Proposing Group Activities (1)

Prosecutor Stance (1)

Protection from Harassment (1)

Protective Behavior (1)

Proudness (1)

Providing Information (1)

Providing Link for Details (1)

Providing Opportunity for the Other to Initiate (1)

Psych Ward Admissions (1)

Psychology Career (1)

Psychotic Behavior (1)

Public Information Misuse (1)

Public Knowledge Limitation (1)

Public Persona (1)

Purchasing Power (1)

Pursuing Better Opportunities (1)

Pursuing Passion (1)

Pursuit of Pleasure (1)

Putin's Nuclear Doctrine (1)

Putting Kids First (1)

Putting Others Down (1)

Putting Yourself Out There (1)

Quality over Quantity (1)

Questioning Others' Listening Skills (1)

Quoting David M. Eagleman (1)

R/RelationshipAdvice (1)

Race Track Background (1)

Rapid Transition (1)

Rare Passion (1)

Reaction Retraining (1)

Reactivity to Notifications (1)

Readiness for Death (1)

Reading Impairment (1)

Realism (1)

Realism vs. Pessimism (1)

Reality Testing (1)

Reason for Living (1)

Reason for Non-Voting (1)

Reasoning for Not Removing Comments (1)

Reasons to Stay (1)

Rebellion (1)

Rebirth (1)

Recall of Past Conversations (1)

Recall of Past Posts (1)

Recipient Check (1)

Recipient Identification (1)

Reciprocal Interaction (1)

Reciprocated Feelings (1)

Recognition of Misery (1)

Recognition of Others (1)

Recognizing Bodily Responses (1)

Recognizing Efforts (1)

Recognizing Exceptional Service (1)

Recognizing Extremes (1)

Recognizing Goodness in the World (1)

Recognizing Own Pathologies (1)

Recoil and Dismissal (1)

Recollection as a Strategy (1)

Recollection of Past Interaction (1)

Reconnecting with Colleagues (1)

Reconsidering Desires (1)

Recurring Statement (1)

Red Cross Aid (1)

Reddiquette (1)

Reddit Post (1)

Reddit r/SuicideWatch and Hotline List (1)

Reddit use (1)

Redemption Through Performance (1)

Redirect to Megathread (1)

Reduced Blood Flow (1)

Reduced Platform Use (1)

Reduced Positive Energy (1)

Reduced Screen Time (1)

Reduced Sexual Drive (1)

Reducing Aging Effects (1)

Reducing Carbohydrates (1)

Reducing Clutter (1)

Reducing Cortisol (1)

Reducing Multitasking (1)

Reducing Offensive Reactions (1)

Reducing Sugars (1)

Reevaluating Relationship (1)

Reevaluation (1)

Reference to Authority (1)

Reference to Delivery Service (1)

Reference to Hitchhiker's Guide to the Galaxy (1)

Reference to Refuting Comments (1)

Referral to Care (1)

Referring to Other Platforms (1)

Reflect on Interactions (1)

Reflection on Past Actions (1)

Reflex Response (1)

Refusal of Repayment (1)

Reimagining the Scenario (1)

Reject Complexity (1)

Rejecting Commodity View of Children (1)

Rejecting Trivialization (1)

Rejection of Competition (1)

Rejection of Dysfunction (1)

Rejection of Materialism (1)

Rejection of Societal Norms (1)

Rejection of Supervisor (1)

Rejection of Traditional Work Ethic (1)

Relatable Behavior (1)

Relating Back to Themselves (1)

Relative Severity (1)

Relaxing Beverage (1)

Relevant Topics (1)

Reliability (1)

Reliance on Assistance (1)

Reliance on Significant Other (1)

Religion (1)

Religious Groups Banding Together (1)

Religious Pressure (1)

Religious Views (1)

Remembering the Deceased (1)

Remodeling (1)

Removal Due to Reason (1)

Removal Reason (1)

Removal of Comments (1)

Removal of Means (1)

Removal of Toxic Comments (1)

Removing COVID19-related Posts (1)

Renting (1)

Repetitive Content (1)

Reply Guidelines (1)

Reporting Process (1)

Reporting to Admins (1)

Request for Clarification (1)

Request for Completed Playlist (1)

Request for Deferment (1)

Request for Dialogue (1)

Request for Justification (1)

Request for Source (1)

Request for Techniques (1)

Request for Update (1)

Required by Law (1)

Requirement for Reasonable Accommodations (1)

Rescue and Prevention (1)

Researching Menus (1)

Resentment Towards Existence (1)

Residence (1)

Residency Match (1)

Resistance to Being Signed Off (1)

Resistance to External Opinion (1)

Resistance to Help (1)

Resistance to Norms (1)

Resolution (1)

Resolution Seeking (1)

Resource Listing (1)

Resource PDF (1)

Restricted Activities (1)

Resume and Career Center (1)

Resuming Driving (1)

Return to Normal Life (1)

Returning Lost Items (1)

Returning to Neutral (1)

Returning to the Thread (1)

Reusability (1)

Reversal of Blindness (1)

Reverse Psychology (1)

Review of Key Points (1)

Revisiting Past Activities (1)

Rhythm and Flow (1)

Ridicule and Mockery (1)

Right to Become Better (1)

Right to Exist (1)

Right to Make Complaint (1)

Rising Statistics (1)

Robotics (1)

Role Model (1)

Role of Government (1)

Room Condition (1)

Rough Morning (1)

Sacrifice for Siblings (1)

Sad Paradox (1)

Sad Story (1)

Sarcastic Agreement (1)

Saturation of Genre (1)

Scam Perception (1)

Scents (1)

School Fees (1)

Scientific Methods (1)

Scouting Content (1)

Screaming at 22:00 on Tuesdays (1)

Searchability of Techniques Online (1)

Searching for Information (1)

Seasonal Greeting (1)

Secure and Grateful (1)

Sedation (1)

Seeking Cause (1)

Seeking Definition (1)

Seeking Euphoria (1)

Seeking Immediate Help (1)

Seeking Information from Other Sources (1)

Seeking Instant Gratification (1)

Seeking Mind-Numbing Activities (1)

Seeking Other Opportunities (1)

Seeking Referrals (1)

Seeking Someone to Talk To (1)

Seeking Specialized Dentist (1)

Seeking Specialized Help (1)

Seeking a Reason (1)

Selection of Company (1)

Selective Social Efforts (1)

Selective Splurging (1)

Self Coaching Method (1)

Self-Advertisement (1)

Self-Assertion in Some Areas (1)

Self-Belief (1)

Self-Capability (1)

Self-Choice (1)

Self-Defense (1)

Self-Disparagement (1)

Self-Efficacy (1)

Self-Introduction (1)

Self-Labeling as Lazy (1)

Self-Limiting Thoughts (1)

Self-Mislabeling (1)

Self-Proof (1)

Self-Sacrificing Behaviors (1)

Self-Study with Textbook (1)

Self-focused Recollection (1)

Self-validity (1)

Self-work Importance (1)

Selling Handmade Products (1)

Sending Good Energy (1)

Sensation of Something Stuck (1)

Sensation of Unreality (1)

Sense of Being Seen (1)

Sense of Ending (1)

Sense of Finality (1)

Sense of Loss (1)

Sense of Peace (1)

Sense of Surreality (1)

Sense of Unworthiness (1)

Sense of Wasted Life (1)

Sensor Functioning (1)

Sensory Appeal of Food (1)

Sensory Stimulation (1)

Separation of Self from Ideas (1)

Setting Alarm (1)

Setting Up for Failure (1)

Sexual Orientation and Identity (1)

Shared Activity (1)

Shared Background (1)

Shared Uncle (1)

Sharing Personal Information (1)

Sharing Personal Past (1)

Sharing Problems (1)

Sharing Resources (1)

Sheltered Upbringing (1)

Shetland (1)

Shift Changes (1)

Shift Towards Logical Thinking (1)

Shift in Beliefs (1)

Short Staffing (1)

Short Tempered (1)

Short-term Planning (1)

Shutdown Response (1)

Shutting Down (1)

Shyness (1)

Siblings as Caregivers (1)

Sign-up Problem (1)

Silencing Natural Reflexes (1)

Silent Interaction (1)

Similar Appearance (1)

Similar Situation (1)

Similar Techniques (1)

Simple Tasks (1)

Single Motherhood (1)

Single Parenthood (1)

Single Programmer Development (1)

Situation Removal (1)

Skepticism Towards Trends (1)

Skepticism about Change (1)

Skepticism of Police Assistance (1)

Skepticism towards AI (1)

Skin Disorders (1)

Skin Issues (1)

Slang (1)

Slow Response to Others (1)

Slower Speech (1)

Small Exposures (1)

Smugness Over Rule Breaking (1)

Social Exclusion (1)

Social Hangover (1)

Social Hangovers (1)

Social Masking (1)

Social Regression (1)

Social and Intellectual Regression (1)

Societal Standards for Men (1)

Soft Voice (1)

Solitary Confinement (1)

Somatic Manifestation (1)

Somatic Obsession (1)

Sonic Revelations (1)

Soul Damage (1)

Sounds (1)

Sour Candy Technique (1)

Sparring (1)

Special Bond (1)

Special Vibe (1)

Specific Product Request (1)

Specific Task (1)

Specific key characters request (1)

Spectrum of Conditions (1)

Speech Degradation (1)

Speed Walking (1)

Spiraling Effect (1)

Spiraling Thoughts (1)

Spirits Lifted (1)

Sponsor Guidance (1)

Spontaneous Requests (1)

Sporadic Energy Surges (1)

Sports Affiliation (1)

Standards for Food Disposal (1)

Stargazing (1)

Starting Capoeira (1)

Starting Over (1)

Starting Point Strategy (1)

Starting and Stopping Projects (1)

Startling Change (1)

Starvation Analogy (1)

Stating the Obvious (1)

Status Quo (1)

Stories Bringing Back to Reality (1)

Story Verification (1)

Strangeness and Popularity (1)

Stranger's Approach (1)

Strangers' Care (1)

Strategic Thinking (1)

Strategies for Managing People (1)

Streaming Platform (1)

Strong Bond (1)

Strong Opinions (1)

Structured Schedule (1)

Struggle through Education (1)

Struggling with Change (1)

Stuck in Unhealthy Patterns (1)

Student Loans (1)

Study Techniques (1)

Studying Schedule (1)

Stuffed Animals (1)

Stunted Emotional Development (1)

Subconscious Emotional Processing (1)

Subconscious Processing (1)

Subjective Normal (1)

Subjectivity of Meaning (1)

Substitution Strategy (1)

Sudden Onset (1)

Suffocation Feeling (1)

Suggesting Blood Sugar Monitoring (1)

Suggesting Legal Action (1)

Suggesting Options (1)

Suicide and Christmas (1)

Suicide as Selfish or Cowardly (1)

Sun Protection (1)

Sunrise (1)

Superficial Wellness (1)

Supportive Comments (1)

Suppressing Critical Internal Voice (1)

Surgical Solutions (1)

Surrender Approach (1)

Susceptibility to Messages (1)

Suspicion (1)

Suspicion of Intent (1)

Swallowing (1)

Sweet Personality (1)

Sympathetic Response (1)

Symptom: Dehydration (1)

Synchronization (1)

Synonym for Offended (1)

Taboo Topics (1)

Taco Preference (1)

Tacos Preference (1)

Taking Advice (1)

Taking Care of Pregnant Partner (1)

Taking Chance on Unknown Language (1)

Taking Ownership (1)

Taking Things Less Seriously (1)

Talk to your parents (1)

Talkativeness with Friends (1)

Talking Points (1)

Talking to Friends and Family (1)

Talking to Someone (1)

Targeting Veterans (1)

Task Specific Risk Assessment (1)

Teacher Assistance (1)

Technical Skills for Publishing (1)

Teenage Ego (1)

Television (1)

Temporal Atmospheres (1)

Temporal Shift of Emotions (1)

Temporary Care (1)

Temporary nature of Feelings (1)

Tension with Parents (1)

Termination (1)

Terminology Usage (1)

Tetris (1)

Text Messaging (1)

Text-Post Only (1)

Thanking Anonymous Gifter (1)

Thanks for Sharing (1)

Theft (1)

Thinking Outside the Box (1)

Third Party Involvement (1)

Thought-Provoking (1)

Tics (1)

Time of Day Influence (1)

Tips for Driving Test (1)

Tissue Energy Conservation (1)

Tool Packs (1)

Torturous Feelings (1)

Toxic Online Communities (1)

Toxic Shopping Sprees (1)

Trading Behavior (1)

Trampoline (1)

Trampoline Park (1)

Transforming Waste (1)

Transience of Material Possessions (1)

Transition from passivity to assertiveness (1)

Transition in Views (1)

Transition to Single Life (1)

Transitional Housing (1)

Transmission to Descendants (1)

Treatment of Vaginismus (1)

Trial and Error (1)

Triggered by Comments (1)

Trivial Causes (1)

Trivializing Conditions (1)

Trivializing Serious Conditions (1)

Troll Management (1)

Trolling Activity (1)

Trolling Issues (1)

Trouble with Eating (1)

Trusting Inner Voice (1)

Turning on Camera (1)

Turnip (1)

Twisted Sense of Rightness (1)

Two People on Phones (1)

Typical Homeless Requests (1)

UK Government Response (1)

Unanswered Questions (1)

Unapparent Causes (1)

Unauthorized Access (1)

Uncertain Ownership (1)

Unchanging Opinions (1)

Unclear goals (1)

Uncomfortable Environment (1)

Uncompleted Assignments (1)

Unconventional Preferences (1)

Unconventional Profession (1)

Undefined Content (1)

Undefined Troubles (1)

Undereating Causes (1)

Underestimation (1)

Underperformance (1)

Undignified Death (1)

Unemotional (1)

Unemployment Due to Health (1)

Unexpected Attachment (1)

Unexpected Comment (1)

Unexpected Events (1)

Unexpected Onset (1)

Unexpected Sources (1)

Unexpected Storyline (1)

Unfair Assignment (1)

Unfair Pricing (1)

Unfair Rehiring (1)

Unfamiliar Surroundings (1)

Unfamiliarity with Current Artists (1)

Unfinished Projects (1)

Unfortunate Events (1)

Unfulfilled Desires (1)

Unhappiness as the Norm (1)

Unhappiness at Work (1)

Unhealthy Lifestyle (1)

Unhealthy Situations (1)

Unhelpful Assumptions (1)

Unidentifiable Feelings (1)

Uninformed Statements (1)

Uninstalling Game (1)

Unique Knowledge (1)

Unique Phenomenon (1)

Unique Traditions (1)

Unity of Being (1)

Universal Beliefs Among Immigrant Parents (1)

Unknown Bathroom Activities (1)

Unknown Cause (1)

Unlearning Disordered Reactions (1)

Unlikelihood (1)

Unplanned Pregnancy (1)

Unplugged Version (1)

Unpopular Opinion Sharing (1)

Unpreparedness (1)

Unprofessionalism (1)

Unraced Greyhound (1)

Unremembered Culprit (1)

Unrestricted Living (1)

Unsatisfaction (1)

Unspecified Location (1)

Unspecified Mood (1)

Unspecified Occurrence (1)

Unsustainable Facade (1)

Unusual Activity (1)

Unusual Request (1)

Unwanted Group Attention (1)

Unwanted Guests (1)

Unwanted Opinions (1)

Unwillingness to Forgive (1)

Upcoming Content (1)

Updating Resume (1)

Uplifting Feeling (1)

Upvoting Based on Edits (1)

Upvoting Behavior (1)

Upvoting Habits (1)

Urge for Bowel Movement (1)

Urgency in Helping Current Sufferers (1)

Urging to Leave (1)

Use of Blackout Curtains (1)

Use of Car for Coping (1)

Use of Checklists (1)

Use of Digital Platforms (1)

Use of Heart Emojis (1)

Use of In-group Language (1)

Use of LOL and HAHA (1)

Use of Oximeter (1)

Use of Visual Aid (1)

User Reference (1)

Using 'Hide' Feature (1)

Using Kids Shopping Carts (1)

Using Phrases (1)

Using Protective Ear Wear (1)

Using YouTube for Guided Activities (1)

Utility in Crisis (1)

Utopian Society (1)

Vaccine Misinformation (1)

Validity of Emotions (1)

Valuing Family Bonds (1)

Valuing New Normal (1)

Valuing Partners (1)

Valuing Togetherness (1)

Valuing Truthfulness (1)

Variability by Topic (1)

Variability in Brain Chemistry and Nervous System (1)

Variability of Management Skills (1)

Varied Outcomes (1)

Vegetative State (1)

Verbalization (1)

Verification Voluntariness (1)

Video Calls (1)

Video Chats (1)

Video Link (1)

Video Playback Interruption (1)

Video Recommendation (1)

Video Resources (1)

Viewer Discretion (1)

Vindictive Manager (1)

Violation of Basic Rights (1)

Violent Daydreaming (1)

Visceral Experiences (1)

Vision Impairment (1)

Vision Issues (1)

Visual Appeal (1)

Visual Influence (1)

Vocation No Insulation (1)

Volume Regulation (1)

Volunteer work (1)

Volunteering Opportunities (1)

Wanting to Maintain Distance (1)

Warm Beverages (1)

Warm Shower (1)

Warning to be Careful (1)

Waste Management (1)

Wasted Opportunities (1)

Water Pressure (1)

Wayback Machine (1)

Websites for Information (1)

Weekly Image Thread (1)

Weird Behavior (1)

Weird Mechanism (1)

Welcoming Death (1)

Welding (1)

Well-meaning Intentions (1)

Wellness Channels (1)

Wellness Promotion (1)

Whataboutism (1)

Wider Audience Reach (1)

Wildness (1)

Will Power Oversimplification (1)

Willful Defiance (1)

Willingness to Listen (1)

Willingness to Revert (1)

Willingness to Trade (1)

Wish for a Gun (1)

Wish to Learn New Skills (1)

Wishing for November to Be Over (1)

Wishing for Peace (1)

Wishing to be Normal (1)

Witnessed Ongoing Fights (1)

Words to Live By (1)

Work Absenteeism (1)

Work Frequency (1)

Worries about Motherhood (1)

Worries of Projecting Personal Issues (1)

Worth Fighting For (1)

Worth of Sharing (1)

Year Reference (1)

Yearning for Easier Solutions (1)

Yearning for Past Self (1)

Yes Men (1)

YouTube Channel Promotion (1)

YouTube Channels (1)

YouTube Link (1)

YouTube Videos (1)

Zoning Out (1)

Zoom Calls with Children (1)

r/petioles (1)
