# Supplementary material for: Identifying Yalom’s group therapeutic factors in anonymous mental health discussions on Reddit: a mixed-methods analysis using large language models, topic modeling and human supervision
Source: Front Psychiatry. 2025 Jun 9;16:1503427. doi: 10.3389/fpsyt.2025.1503427 (PMC12183517; doi:10.3389/fpsyt.2025.1503427)
Supplement: Supplementary file 1 [file DataSheet1.zip › Appendix D.docx]

**Appendix D**

**Ethical considerations for the use of reddit data for this research.**

Ethical approval was not required for the study involving human data in accordance with the local legislation and institutional requirements. Written informed consent was not required, for either participation in the study or for the publication of potentially/indirectly identifying information, in accordance with the local legislation and institutional requirements. The social media data was accessed and analyzed in accordance with the platform's terms of use and all relevant institutional/national regulations.

Reddit, a popular platform for user-generated content within topic-specific subreddits, is publicly accessible through active forums, archives, and APIs, as outlined in its privacy policy (Reddit Privacy Policy, 2024). According to Reddit's privacy policy, the data can be used for research purposes (Reddit Privacy Policy, 2024). However, this open accessibility raises important ethical questions, particularly in health-related subreddits. Despite the pseudonymity, there may exist the risks of (partial) re-identification (Gliniecka, 2023; Fiesler et al., 2024). Verbatim quotes or contextual information could enable re-identification of users, especially when throwaway accounts are not used or personal information is willingly shared within the user account history. Therefore, additional privacy-preserving techniques, such as automated anonymization procedures and paraphrasing, should be applied. Additionally, direct quotes should be rephrased to avoid reverse-searching for the original data. Moreover, while users accept the terms of service and agreement, their perceptions of privacy might still diverge from Reddit's public nature, as sensitive health information may be shared with an expectation of implicit confidentiality — i.e., users may not expect their data to be used for research or other purposes (Gliniecka, 2023; Fiesler et al., 2024). This makes a careful risk-benefit assessment by the researchers essential.

Health-related subreddits may be used by vulnerable groups, among others, which requires researchers to provide additional sensitivity to avoid reproducing potentially stigmatizing narratives (Fiesler et al., 2024). The large scale and anonymity of Reddit make it impossible to obtain individual consent from all users. However, ethical considerations can be addressed through appropriate dialogue with moderators ("admins") and by respecting subreddit guidelines (Gliniecka, 2023; Fiesler et al., 2024). Researchers must carefully consider and weigh potential harms and risks associated with the research. Additionally, researchers should reflect on their own positionality and power dynamics, prioritize the well-being of online communities and the people behind them, and ensure responsible representation of the results. Adherence to evolving platform policies is also necessary (Gliniecka, 2023; Fiesler et al., 2024).

This research project aims to address these ethical considerations through the following procedures:

Data minimization was ensured by extracting only the top-rated comments. Other data, such as usernames, metadata (timestamps, post IDs), and standard data categories, were explicitly excluded. The additional anonymization of raw data involved a multi-step process: automated named entity removal using the GLiNER model, followed by manual review to redact potentially identifiable information, including direct or indirect mentions of names, contact details, specific locations or dates, unique personal experiences, as well as employment or education details in linked content (e.g., manually searching for statements such as "I live in," "contact me," "@" etc.). Additionally, all direct quotes in the manuscript were paraphrased to further protect anonymity and prevent traceability through reverse search. However, the "everyday" risks of being online are acknowledged, meaning participation in any online community inherently brings a degree of vulnerability and the potential for loss of anonymity.

Furthermore, recognizing the potential vulnerability of individuals discussing mental health online, a resource-oriented approach was adopted, explicitly aiming to identify support mechanisms and therapeutic processes to highlight positive and constructive aspects of online communities, while also acknowledging the potential risks of negative content. The advanced NLP methods used were validated by experts, with consensus reached through extensive discussions and theoretical embedding of the findings. Finally, this research prioritizes community engagement. The findings will be shared with the subreddits, should the subreddit moderators welcome such dissemination. Affected individuals or community members, whose experiences are reflected (or not) in the data, are explicitly invited to contact the first author. Additionally, the researchers have carefully reviewed the subreddit guidelines. Subreddits that explicitly excluded researchers were excluded from the data collection process.

**Literature**

[Fiesler, C., Zimmer, M., Proferes, N., Gilbert, S., and Jones, N. (2024). Remember the human: A systematic review of ethical considerations in reddit research. *Proc. ACM Hum.-Comput. Interact.* 8, 1–33.](https://www.zotero.org/google-docs/?W7RfUC) doi: 10.1145/3633070.

[Gliniecka, M. (2023). The ethics of publicly available data research: A situated ethics framework for Reddit. *Soc. Media Soc.* 9, 20563051231192021.](https://www.zotero.org/google-docs/?W7RfUC) doi: 10.1177/20563051231192021.

[Reddit Privacy Policy (2024). Available at: https://www.reddit.com/policies/privacy-policy](https://www.zotero.org/google-docs/?W7RfUC)
