# Supplementary material for: Identifying Yalom’s group therapeutic factors in anonymous mental health discussions on Reddit: a mixed-methods analysis using large language models, topic modeling and human supervision
Source: Front Psychiatry. 2025 Jun 9;16:1503427. doi: 10.3389/fpsyt.2025.1503427 (PMC12183517; doi:10.3389/fpsyt.2025.1503427)
Supplement: Supplementary file 1 [file DataSheet1.zip › Appendix E.docx]

**Appendix E.**Assigned clusters to Yalom’s therapeutic group factors.


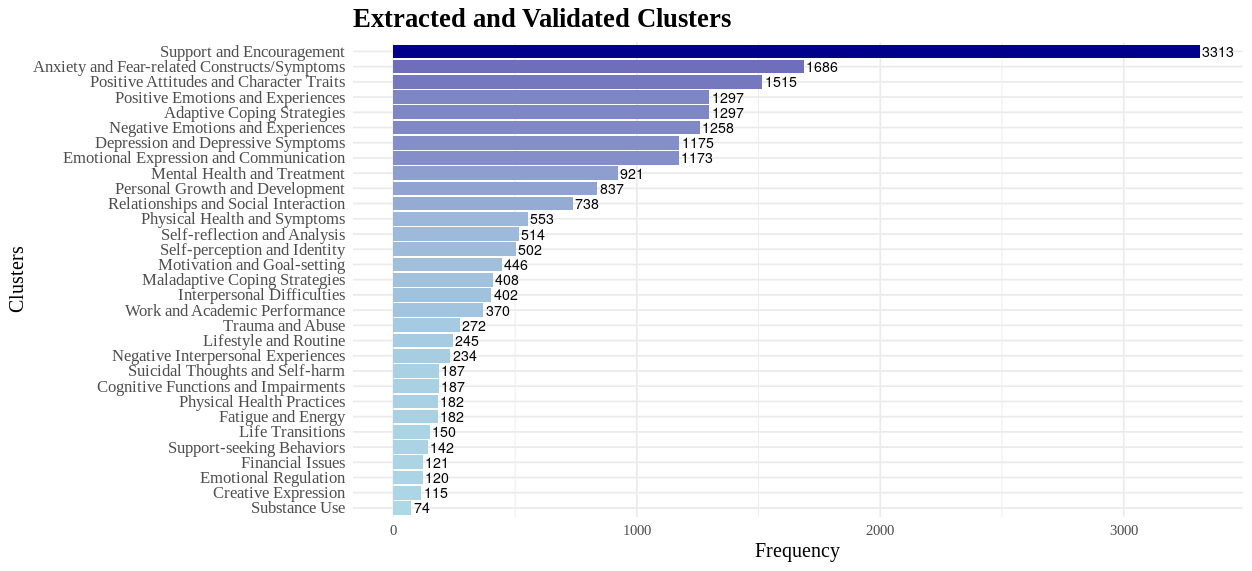


Bar chart of the 31 extracted and validated clusters, with frequency indicating the total count of all codes assigned to each cluster. Each code was exclusively assigned to a single cluster.
