# Supplementary material for: Identifying Yalom’s group therapeutic factors in anonymous mental health discussions on Reddit: a mixed-methods analysis using large language models, topic modeling and human supervision
Source: Front Psychiatry. 2025 Jun 9;16:1503427. doi: 10.3389/fpsyt.2025.1503427 (PMC12183517; doi:10.3389/fpsyt.2025.1503427)
Supplement: Supplementary file 1 [file DataSheet1.zip › Appendix F.pdf]

Appendix F.

```
// ============================================================
// Overall Prompt Versioning Summary:
// ============================================================
// The development of the prompt went as follows:
// 1. Started with a basic instruction to analyze qualitative data.
// 2. Identified the need to segment the data for targeted analysis.
// 3. Introduced a requirement for structured JSON output, addressing faulty outputs and direct replies of the model.
// 4. Added few-shot examples and reinforced the role to ensure the model strictly performs qualitative analysis.
// 5. Enhanced the prompt to include an overall category for consistency and a broader context.
// 6. Added a strategy to prevent potential hallucinations by requiring each code to include a direct quote from the data.
// Note: there were several small adjustments in structure and language that are not shown here
// ============================================================
//
// ============================================================
// Step 0: Explained Logical Annotation & Template Overview
// ----------------------------------------
// Blueprint for the iterative prompt versioning proces
// It lays out the fundamental components and logic behind each subsequent iteration (Steps 1 through 6),
//
// Key Components Defined in Each Iteration:
//   - Thought (T[n]): The conceptual reasoning or goal behind the current iteration.
//   - Prompt (P[n]): The specific instruction or request given to the model.
//   - Result (R[n]): The output produced by the model based on the current prompt.
//   - Adjustment (Δ): The specific change or set of changes made based on issues identified in R[n].
//   - Documentation: A narrative record capturing the issues, observations, and decisions for each iteration.
//
// Extensive Logical Annotation:
//   1. The process starts with an initial thought and prompt (T[0] and P[0]) that establish what we want to achieve.
//      - Example: "Conduct qualitative analysis on data X."
//      - This simple directive, however, is too broad and does not account for the need to analyze small segments or produce structured outputs.
//   2. To remedy this, the process iteratively refines the prompt by:
//      - Segmenting the data into manageable parts.
//      - Mandating a structured JSON output with clearly defined keys and values.
//      - Embedding few-shot examples to guide the model toward consistent output.
//      - Reinforcing the role of the model as a qualitative data analyst, ensuring that it never replies directly to the raw text.
```

//   - Including an overall category to capture the broader context for each set of codes.
//   - Adding a safeguard against potential hallucinations by requiring that every code be anchored by a direct quote (the "example") from the data.
//   3. Each iteration (from Step1 through Step6) addresses specific issues discovered during testing, such as:
//   - Overly broad instructions leading to unsegmented analysis.
//   - Faulty or unstructured JSON outputs.
//   - Direct responses to the data rather than an abstracted analysis.
//   - Inconsistency in the generated codes and lack of an overall framing category.
//   - The potential for hallucinations (i.e., generating codes without concrete evidence) even if it was not a major issue during testing.
//   4. The iterative loop continues until the model's output satisfies all pre-defined criteria (valid JSON, correct segmentation, overall category inclusion, and concrete examples for each code), at which point the process is marked as accepted.
//
// Purpose of This Template:
//   - To systematically track and document the evolution of the prompt from its initial, basic form to a refined version that meets all qualitative analysis requirements.
//

// ===
// Dissection of the Prompt || Prompt Engineering Process
// ===

// Step 1: Initial Basic Instruction
// ----------------------------------
// Basic instruction: "Conduct qualitative analysis on data X."
// - Issue: Too broad; the model attempted a full content analysis on a single comment.
// - Decision: Instruct the model to work on small segments and define the role of the LLM more clearly (separating all sections with “##### “placeholder” e.g. “Your Task/Role””).
Step1:
    BasicPrompt ← "Conduct qualitative analysis on data X."
    Issue: "Full content analysis without segmenting the data."
    Decision: "Instruct the model to analyze data segment-wise."

// Step 2: Incorporating Segmentation Requirement
// --
// Revised prompt: Instruct the model to analyze small segments.
// - Observation: Analyzing the entire text at once was problematic due to multiple semantically distinct concepts.
// - Issue: Needed to extract meaningful meaning units from the raw data.
// - Decision: Explicitly instruct the model to extract and analyze individual segments.
Step2:
    RevisedPrompt ← "Conduct qualitative analysis on data X by analyzing small segments."
    Issue: "Lack of segmentation prevented targeted coding."
    Decision: "Direct the model to segment the text for detailed analysis."

// Step 3: Introducing Structured Output Requirement (JSON Format)

// --
// Added instruction: Output the analysis in JSON format.
// - Observation: Simply stating JSON format did not work in many cases, resulting in faulty JSON and direct replies to comments.
// - Issue: The API lacked structured output capabilities at that time.
// - Decision: Integrate a requirement for valid JSON output and emphasize that the model must strictly act as a qualitative data analyst.
Step3:
   JSONRequirement ← "Organize your analysis in a valid JSON format with defined key-value pairs."
   Issue: "Faulty JSON was generated, and the model replied directly to text snippets."
   Decision: "Enforce valid JSON output and mandate that the model only analyzes data."

// Step 4: Adding Few-shot Examples and Reinforcing Role
// ---
// Introduced few-shot examples to ensure the correct JSON structure and consistency.
// - Observation: Without concrete examples, the model produced inconsistent outputs.
// - Issue: The model continued replying directly to comments rather than analyzing the data.
// - Decision: Provide clear few-shot examples to guide the output structure and reinforce the model’s role as a qualitative data analyst.
Step4:
   FewShotExamples ← "Include several JSON examples demonstrating categories, codes, and supporting quotes."
   ReinforceInstruction ← "NEVER reply to the posts; always perform qualitative analysis on the data."
   Issue: "Direct replies to comments and inconsistent output structure."
   Decision: "Embed few-shot examples and stress the qualitative analysis role."

// Step 5: Enhancing Consistency in Code Generation with an Overall Category
// --
// Improved prompt to reduce variability in generated codes.
// - Observation: Early iterations produced highly variable codes lacking a unifying context.
// - Issue: The model did not consistently generate an overall category to capture the 'greater picture'.
// - Decision: Instruct the model to provide an overall category framing the analysis, along with consistent codes and supporting examples.
Step5:
   OverallCategoryInstruction ← "For each analysis segment, provide an overall category that frames the analysis, along with detailed codes."
   Issue: "Variability in codes and missing broader context."
   Decision: "Ensure the output includes both an overall category and consistent codes with supporting examples."

// Step 6: Addressing Potential Hallucinations
// --
// Even though hallucinations were not an issue during initial testing and prompt engineering, there was concern that they might arise across the large dataset.

// - Observation: Potential for hallucinations was anticipated (e.g., coherent details fully or partially made up by the model).
// - Issue: Without a strategy to prevent this, the model might assign codes without a concrete basis or even make up codes.
// - Decision: Add concrete ("examples") to the JSON format that must always include a direct quote from the data as the foundation for each code assignment.
Step6:
    HallucinationPrevention ← "For every code, include an 'example' key containing a direct quote from the data."
    Issue: "Potential hallucinations where codes might be made up or assigned without existing in the data."
    Decision: "Make sure that each code is anchored with a direct quote from the data."
