# Supplementary material for: Identifying Yalom’s group therapeutic factors in anonymous mental health discussions on Reddit: a mixed-methods analysis using large language models, topic modeling and human supervision
Source: Front Psychiatry. 2025 Jun 9;16:1503427. doi: 10.3389/fpsyt.2025.1503427 (PMC12183517; doi:10.3389/fpsyt.2025.1503427)
Supplement: Supplementary file 1 [file DataSheet1.zip › Appendix G_updated_ii.pdf]

Appendix G: Conceptual Workflow of a Multi-LLM Framework to further automate Thematic Analysis

May 26, 2025

Overview

This document illustrates how multiple Large Language Models (LLMs) and specialized modules can be orchestrated to carry out automated qualitative research process—from data collection to final thematic analysis. In this modular approach, one LLM handles initial coding, while a separate module (or LLM) manages and updates the global codebook.

1 Data Acquisition and Preprocessing

Data Extraction

Let $\mathcal{P}$ be a set of large and anonymous qualitative data sources, suitable for research:

$$D_0 = \text{extractData}(\mathcal{P}, \text{filters}),$$

where filters may specify date ranges, topic filters, or other domain constraints. The function extractData collects raw text via appropriate APIs.

Data Cleaning

A cleaning function clean removes deleted or duplicate posts and normalizes text (e.g., removing hyperlinks, anonymizing usernames):

$$D = \text{clean}(D_0).$$

Further NLP-specific preprocessing is optional, given robust language capabilities in modern LLMs.

2 Dataset Partitioning

Let D denote the complete cleaned dataset, partitioned into n sections to allow for batch-wise or incremental processing:

$$D = \bigcup_{i=1}^{n} S_i.$$

3 Section Coding with LLM

For each section S_i, an **initial coding function** f_1 (implemented by a efficient and capable LLM, e.g., GPT-4o, Claude Sonnet 3.5/3.7, Qwen3, DeepSeek-V3, or similar) produces a set of codes:

$$C_i = f_1(S_i).$$

Here, f_1 identifies one or more short labels (codes) per comment or meaning unit within S_i.

4 Code Unification via Dedicated Module

Let u be a **unifier module** (potentially another LLM) that merges newly generated codes C_i into a global codebook B while avoiding duplicates. For each code $c \in C_i$, u performs the following:

$$B \leftarrow u(c, B, \tau) = \begin{cases} B \cup \{c\}, & \text{if } \forall b \in B,\ \mathrm{sim}(c, b) < \tau, \\ B, & \text{otherwise (if } \exists\, b \in B : \mathrm{sim}(c, b) \geq \tau), \end{cases}$$

where $\mathrm{sim}(c, b)$ is a similarity measure (e.g., cosine similarity) between embeddings of c and b, and τ is a threshold (e.g., 0.85).

5 Iterative Code Tracking and Clustering

After every k sections are processed (i.e., when $i \mod k = 0$), apply a **clustering function** g (implemented by a reasoning LLM or another clustering algorithm) to the aggregated codebook B:

$$\{T_1, T_2, \ldots, T_m\} = g(B).$$

This step groups codes within B into semantically coherent sets (T_j), forming proto-topics or subthemes.

6 Thematic Analysis

Finally, a **thematic analysis function** h (implemented by another reasoning LLM) refines these proto-topics into overarching themes:

$$\text{Themes} = h(T_1, T_2, \ldots, T_m),$$

where h interprets and consolidates the clusters to address the study's theoretical or research objectives (e.g., mapping them onto Yalom's group therapy framework).

7 Iterative Refinement

Define a function validate that uses another reasoning LLM or human domain experts to check the quality of the derived themes. If the themes fail specific criteria (e.g., logical consistency, adequate coverage, lack of alignment with domain knowledge), the pipeline can adjust the threshold τ or re-run clustering:

$$\text{if validate(Themes)} = \text{False} : \quad \begin{aligned} &\tau \leftarrow \tau - \delta; \\ &\{T_1, T_2, \ldots, T_m\} \leftarrow g(B); \\ &\text{Themes} \leftarrow h(T_1, \ldots, T_m). \end{aligned}$$

Repeat until validate(Themes) is true or until a maximum number of refinement iterations is reached.
